# Supplementary figures and images for: MCM5 UFMylation regulates replication origin firing and fork progression
Source: EMBO J. 2025 Sep 12;44(21):6019–50. doi: 10.1038/s44318-025-00562-6 (PMC12583452; doi:10.1038/s44318-025-00562-6)

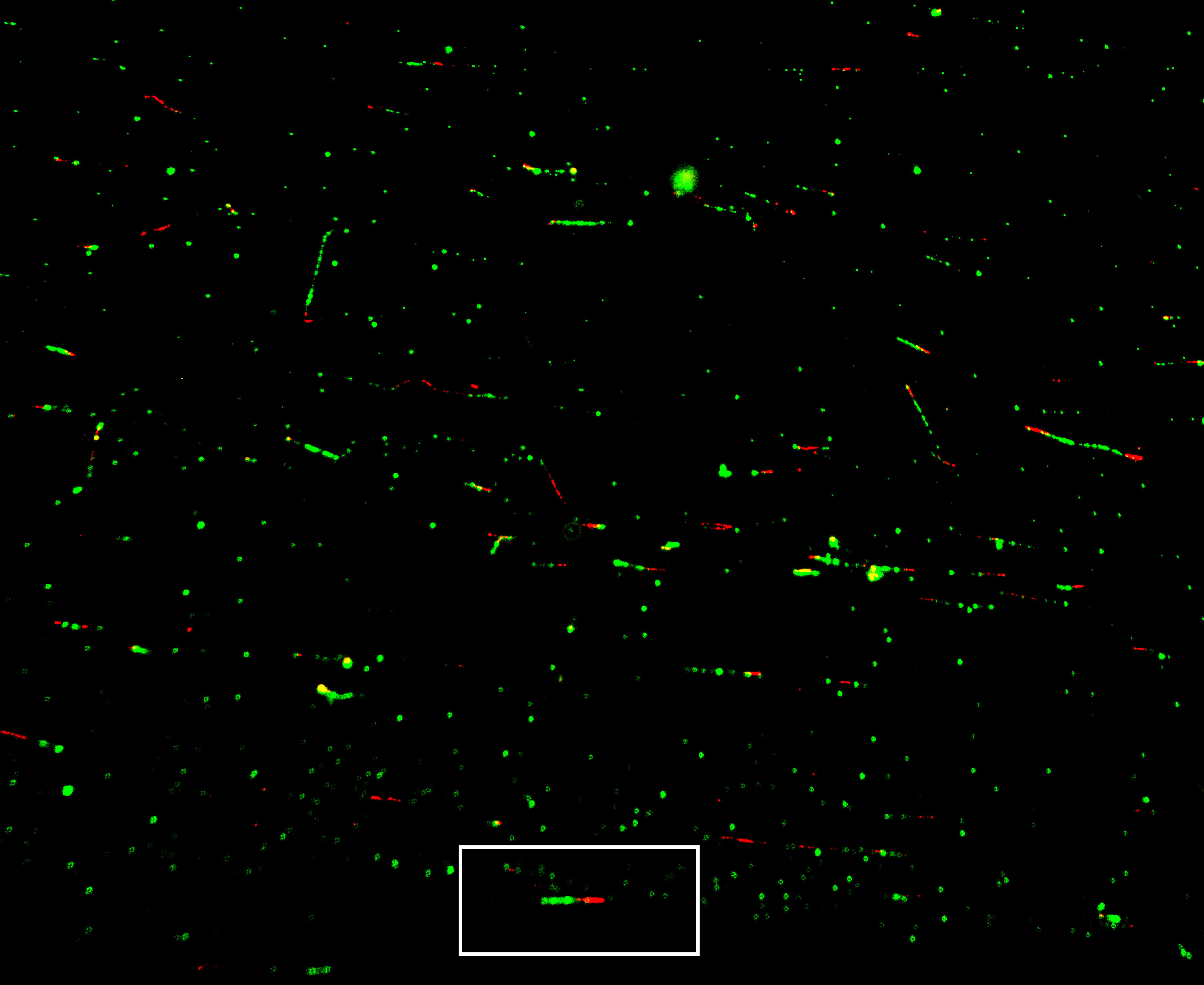

Supplement: Supplementary file 3 — Source data Fig. 1 [file 44318_2025_562_MOESM3_ESM.zip › Figure 1/1F/Fiber DKM 2-93.tif]

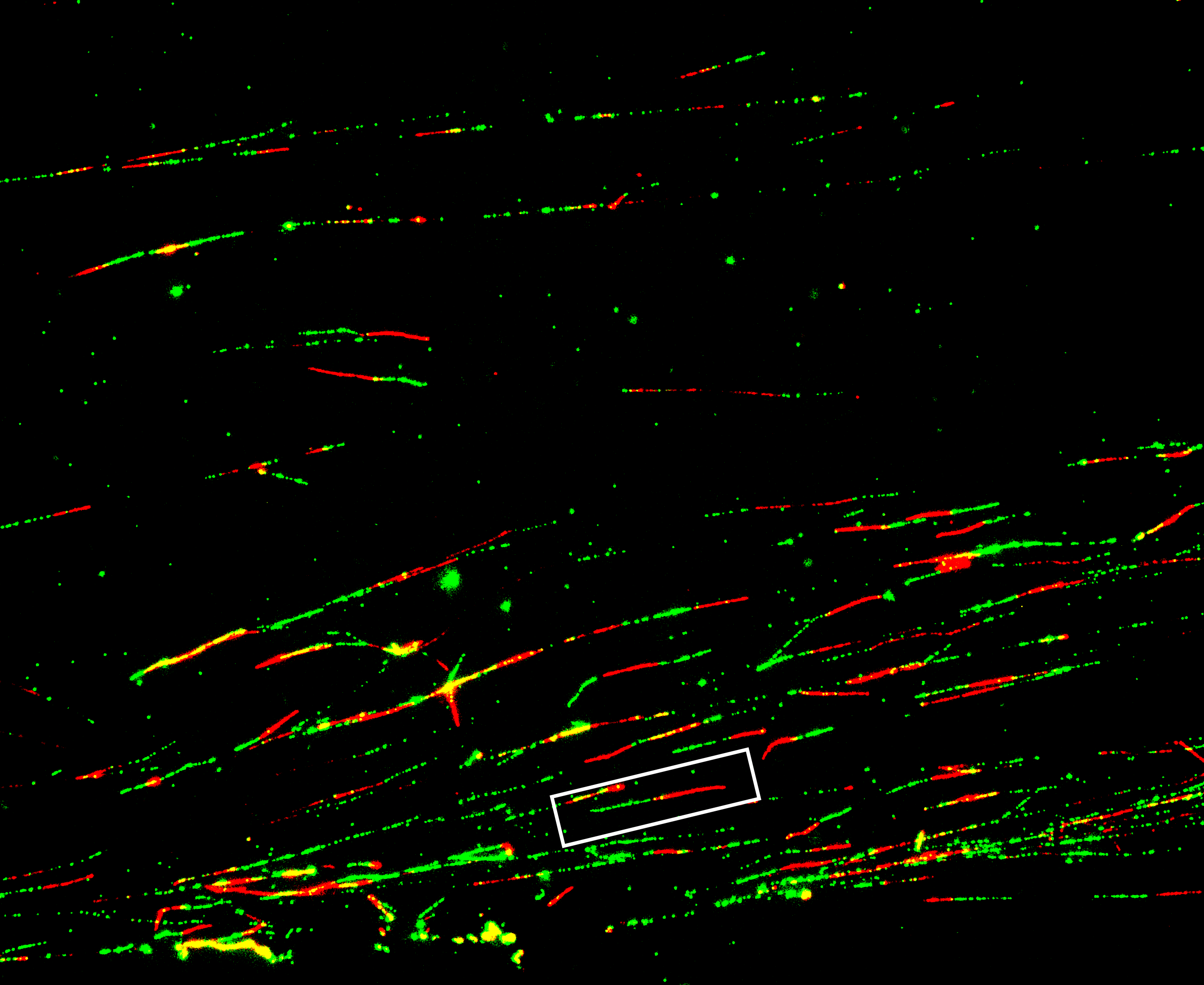

Supplement: Supplementary file 3 — Source data Fig. 1 [file 44318_2025_562_MOESM3_ESM.zip › Figure 1/1F/Fiber DMSO.tif]

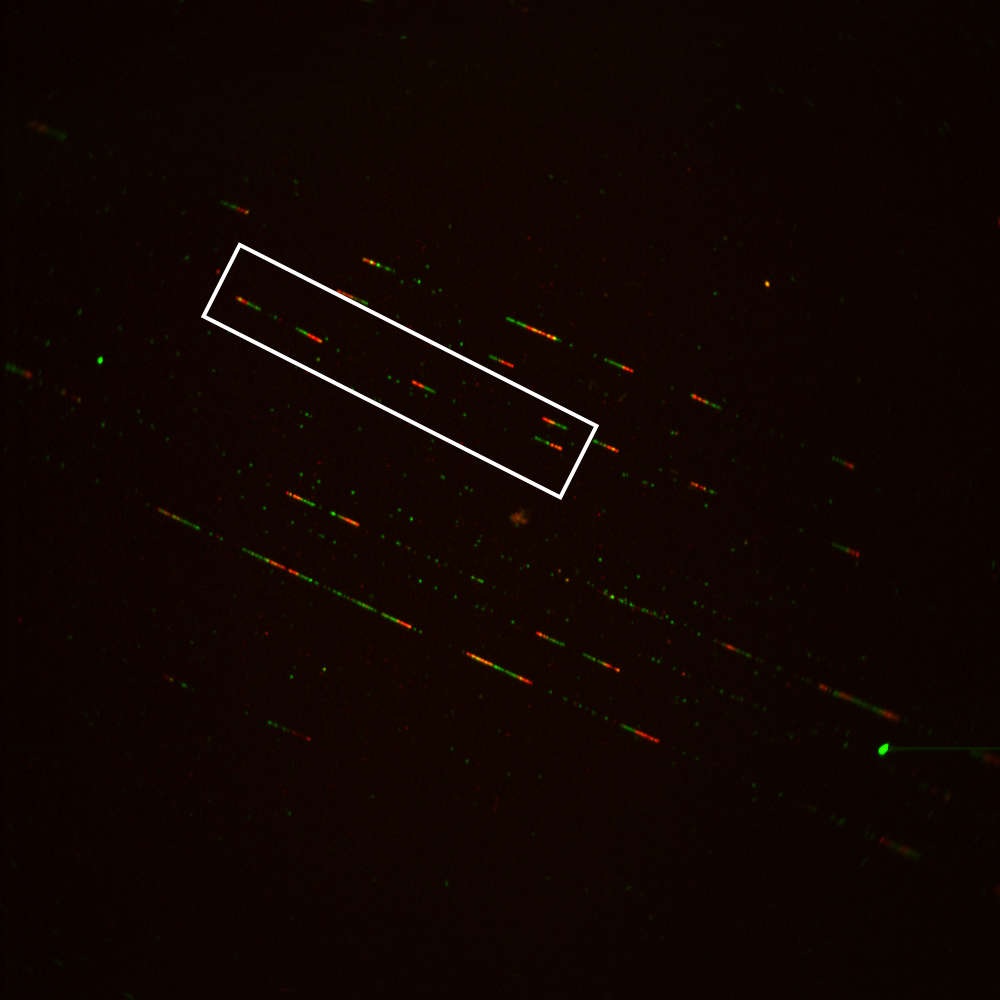

Supplement: Supplementary file 3 — Source data Fig. 1 [file 44318_2025_562_MOESM3_ESM.zip › Figure 1/1D/Fiber siE3.tif]

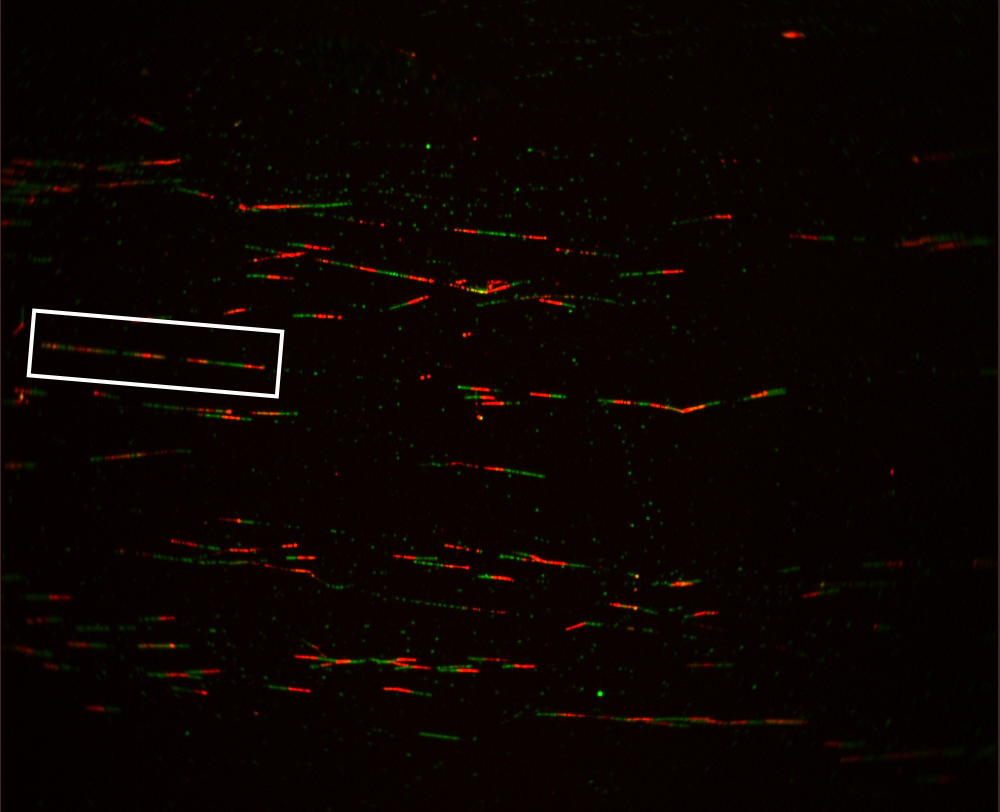

Supplement: Supplementary file 3 — Source data Fig. 1 [file 44318_2025_562_MOESM3_ESM.zip › Figure 1/1D/Fiber siiNC.tif]

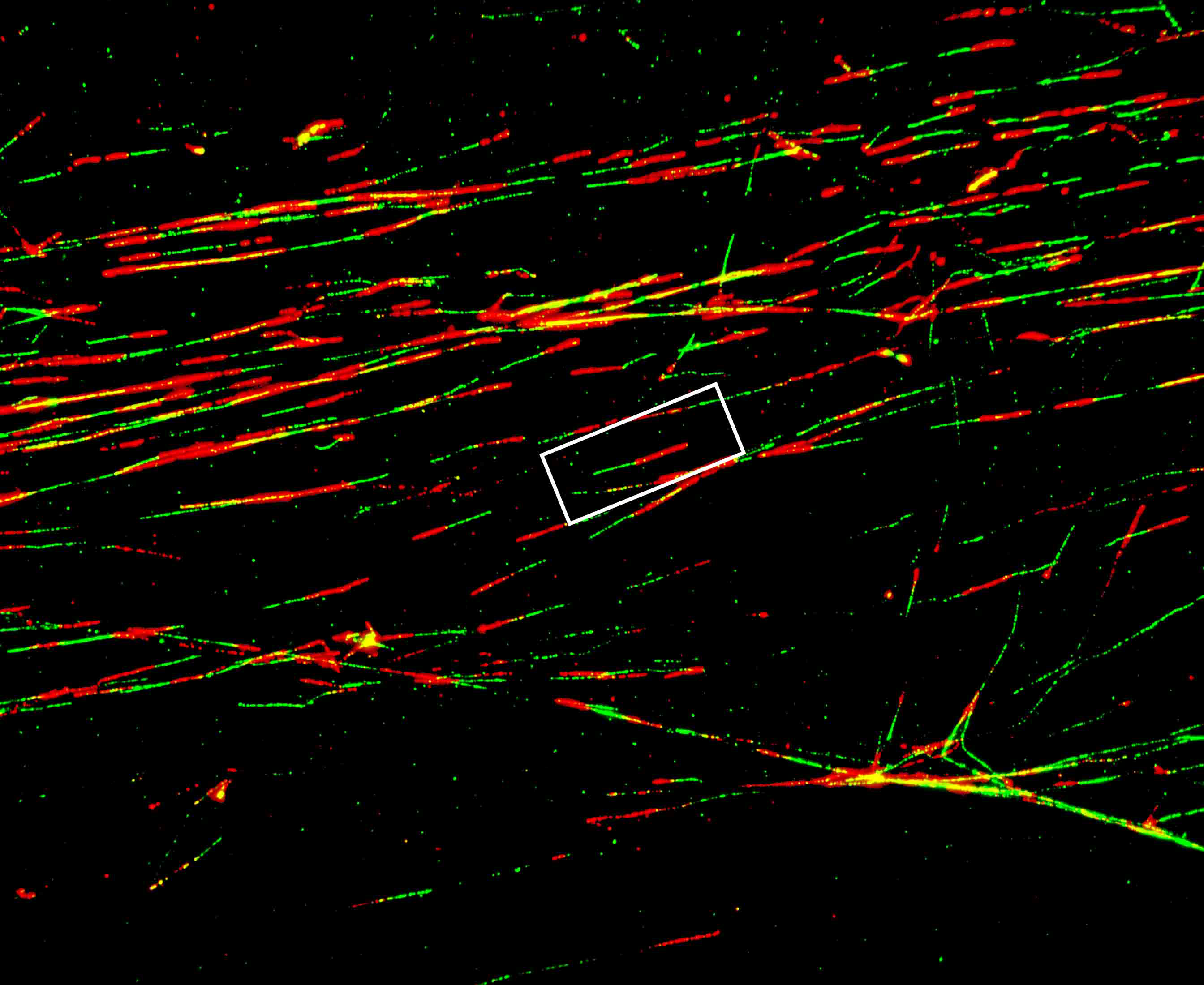

Supplement: Supplementary file 3 — Source data Fig. 1 [file 44318_2025_562_MOESM3_ESM.zip › Figure 1/1E/1E Left/Fiber siE3.tif]

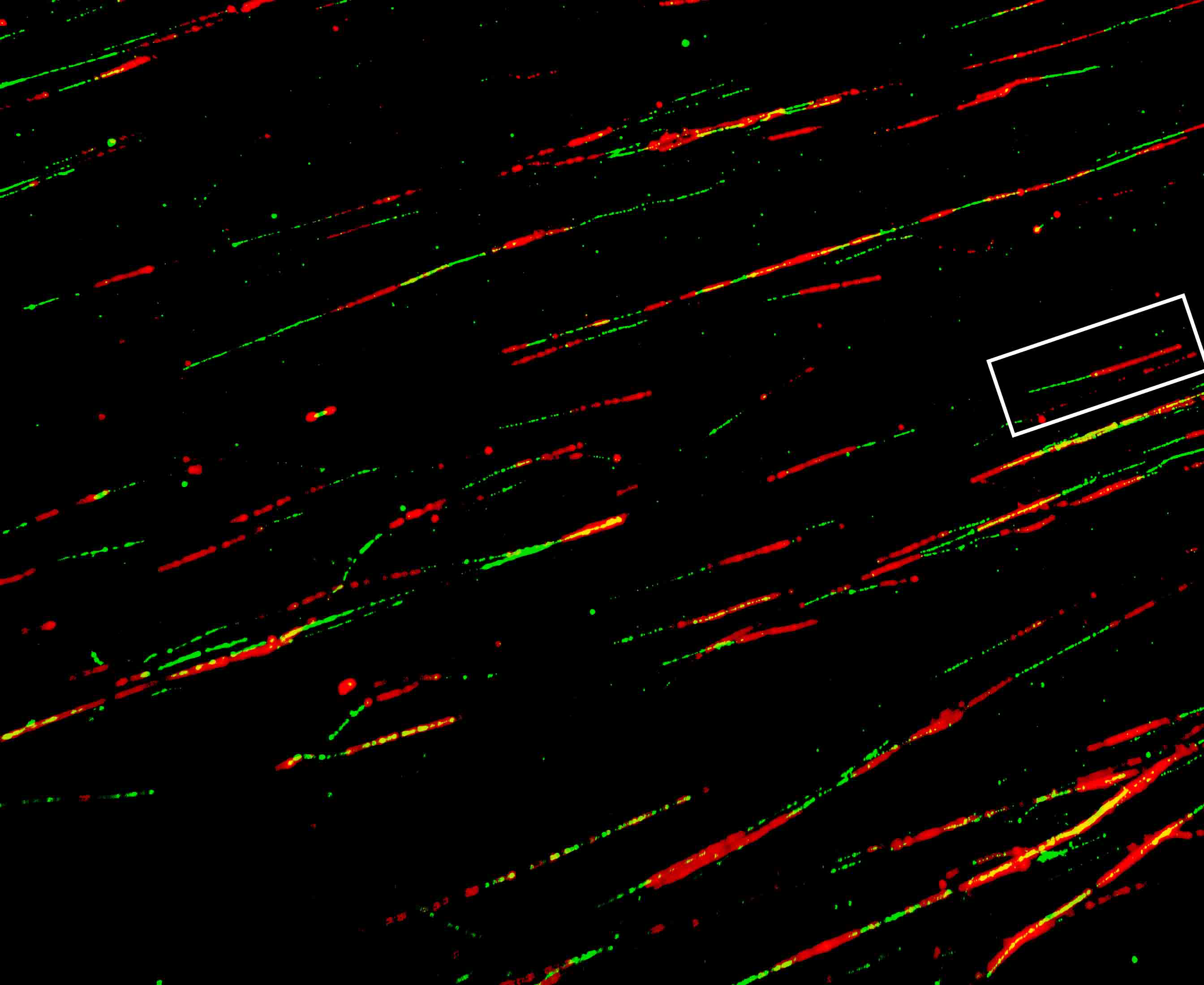

Supplement: Supplementary file 3 — Source data Fig. 1 [file 44318_2025_562_MOESM3_ESM.zip › Figure 1/1E/1E Left/Fiber siE3+E3.tif]

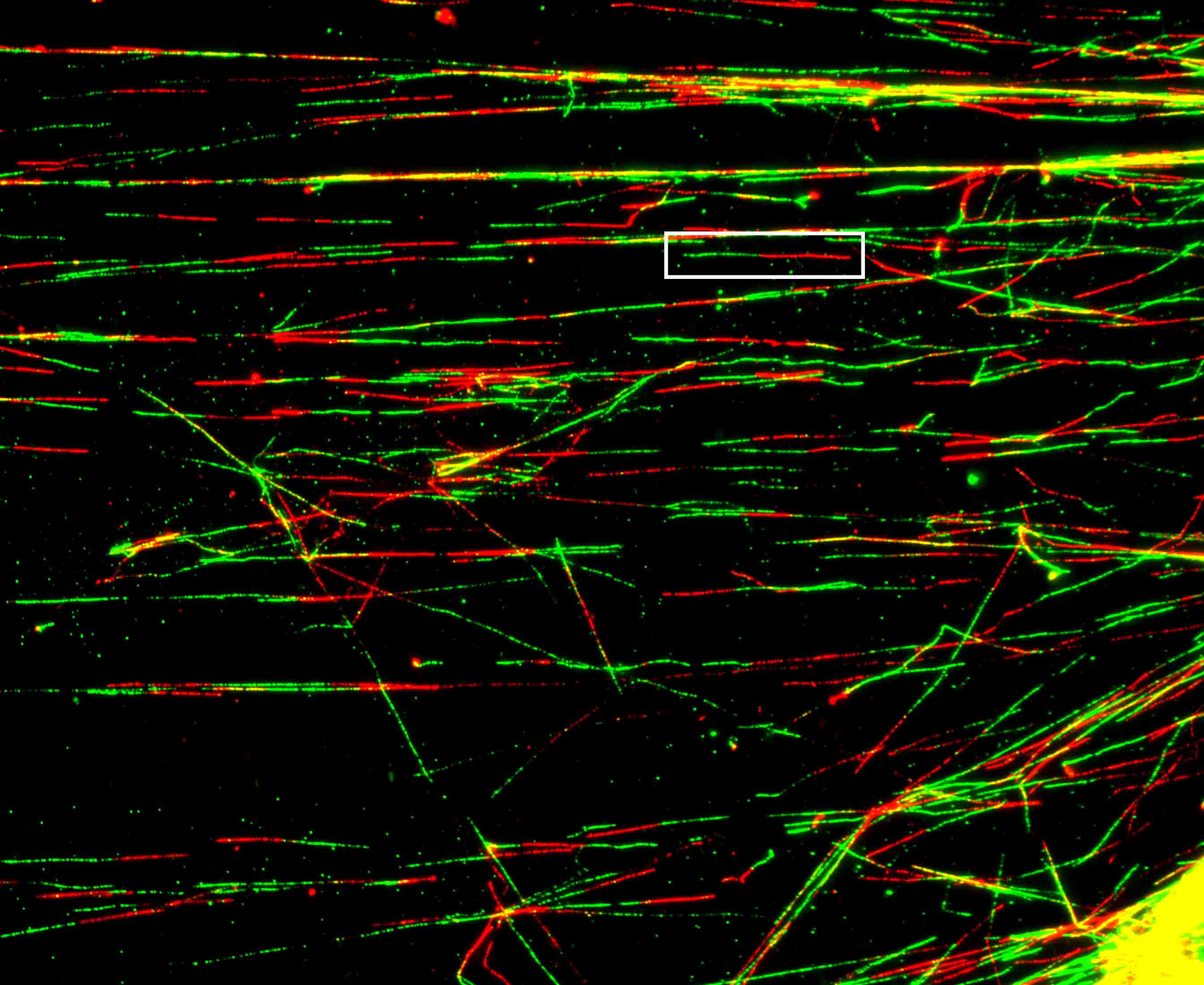

Supplement: Supplementary file 3 — Source data Fig. 1 [file 44318_2025_562_MOESM3_ESM.zip › Figure 1/1E/1E Left/Fiber siNC.tif]

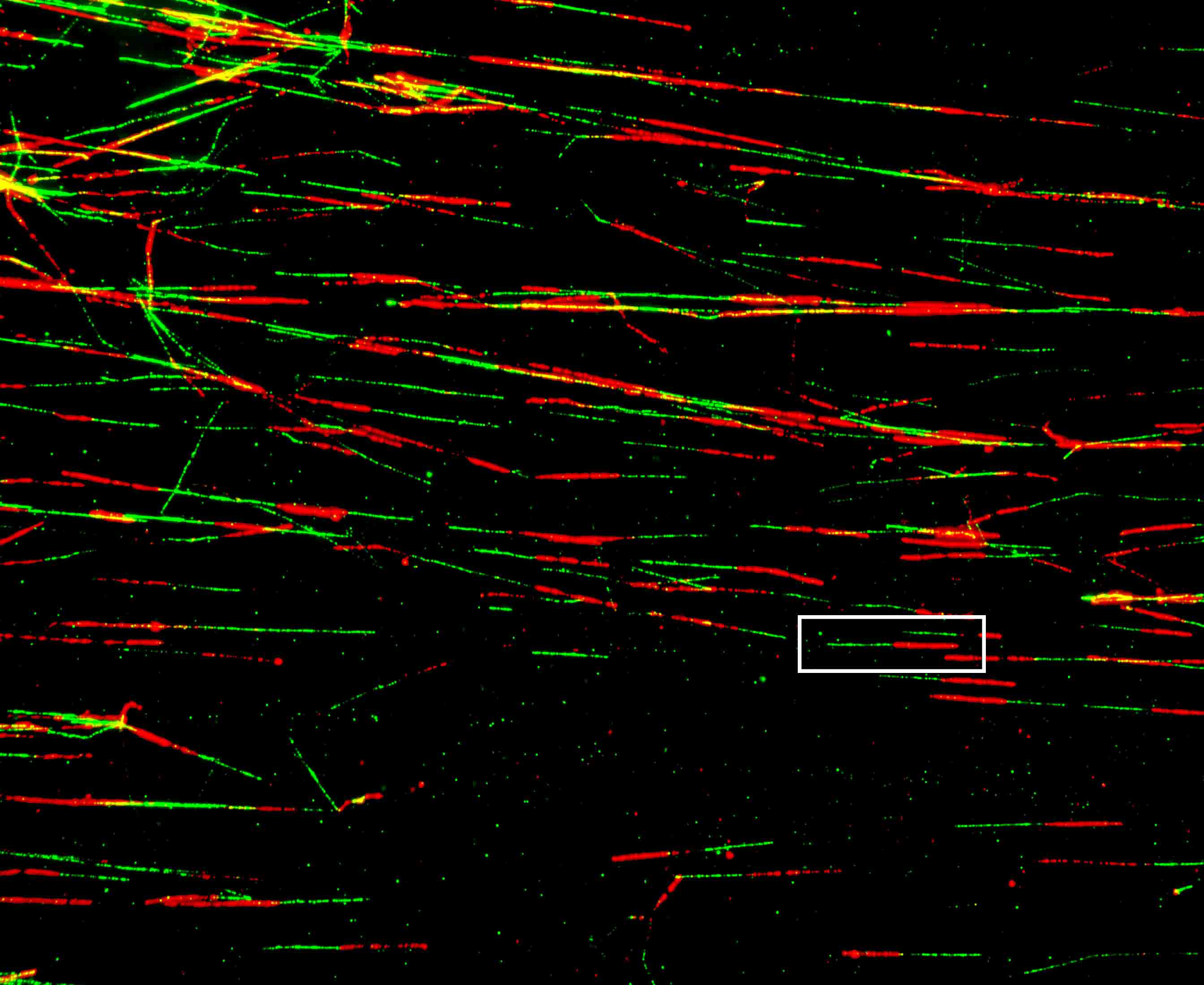

Supplement: Supplementary file 3 — Source data Fig. 1 [file 44318_2025_562_MOESM3_ESM.zip › Figure 1/1E/1E Left/Fiber siE3+VEC.tif]

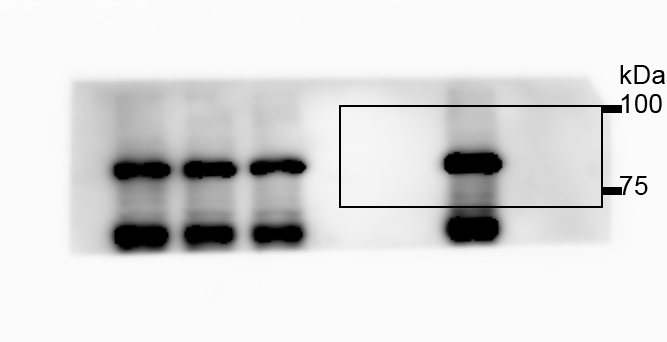

Supplement: Supplementary file 4 — Source data Fig. 2 [file 44318_2025_562_MOESM4_ESM.zip › Figure 2/2A/Western Captured UFL1.tif]

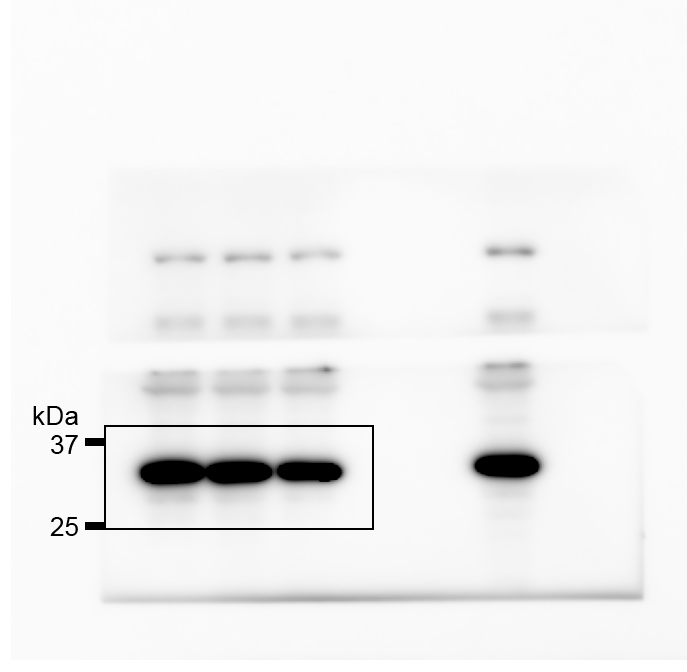

Supplement: Supplementary file 4 — Source data Fig. 2 [file 44318_2025_562_MOESM4_ESM.zip › Figure 2/2A/Western INPUT PCNA.tif]

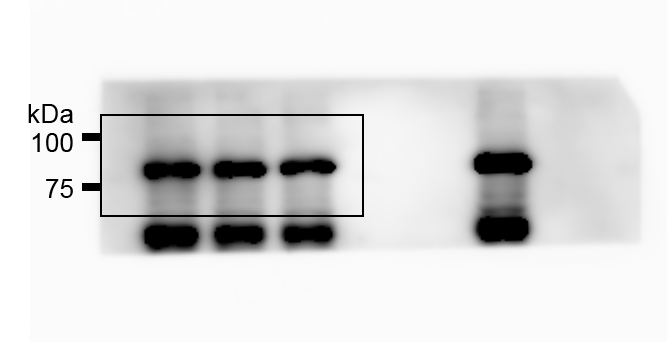

Supplement: Supplementary file 4 — Source data Fig. 2 [file 44318_2025_562_MOESM4_ESM.zip › Figure 2/2A/Western INPUT UFL1.tif]

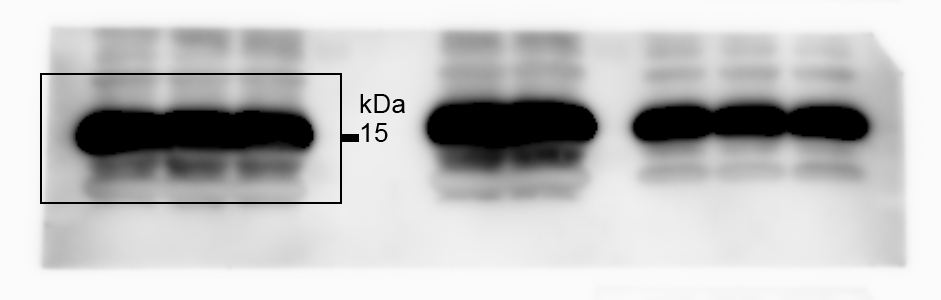

Supplement: Supplementary file 4 — Source data Fig. 2 [file 44318_2025_562_MOESM4_ESM.zip › Figure 2/2A/Western INPUT H3.tif]

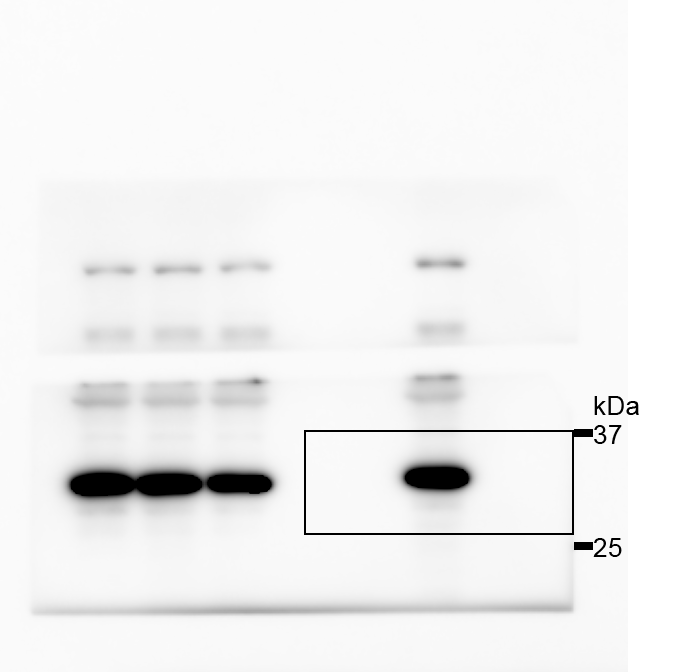

Supplement: Supplementary file 4 — Source data Fig. 2 [file 44318_2025_562_MOESM4_ESM.zip › Figure 2/2A/Western Captured PCNA.tif]

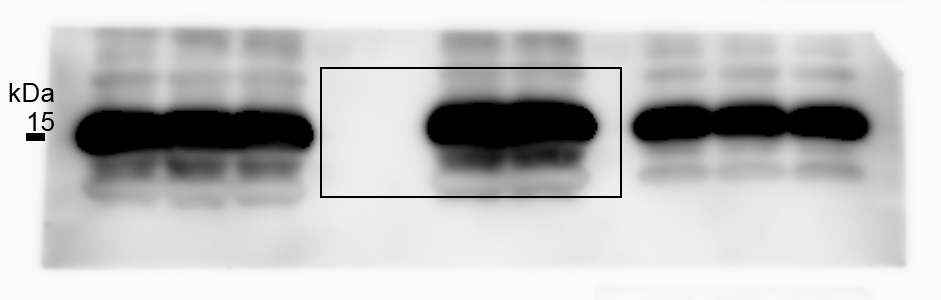

Supplement: Supplementary file 4 — Source data Fig. 2 [file 44318_2025_562_MOESM4_ESM.zip › Figure 2/2A/Western Captured H3.tif]

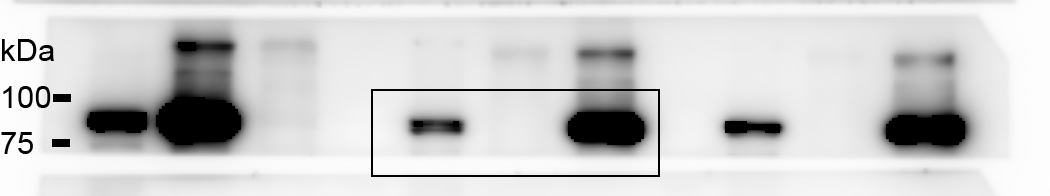

Supplement: Supplementary file 5 — Source data Fig. 3 [file 44318_2025_562_MOESM5_ESM.zip › Figure 3/3C/Western UFL1.tif]

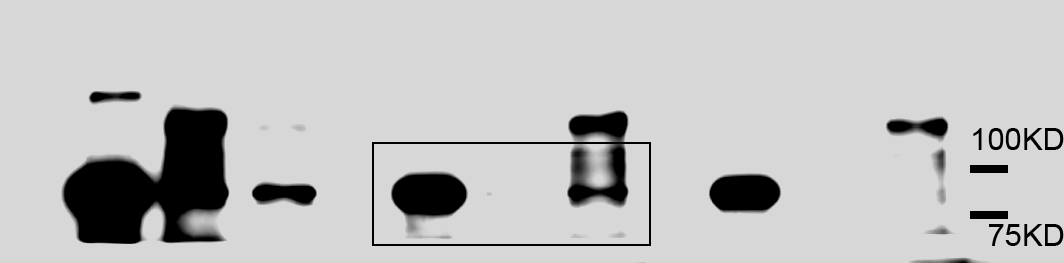

Supplement: Supplementary file 5 — Source data Fig. 3 [file 44318_2025_562_MOESM5_ESM.zip › Figure 3/3C/Western MCM5.tif]

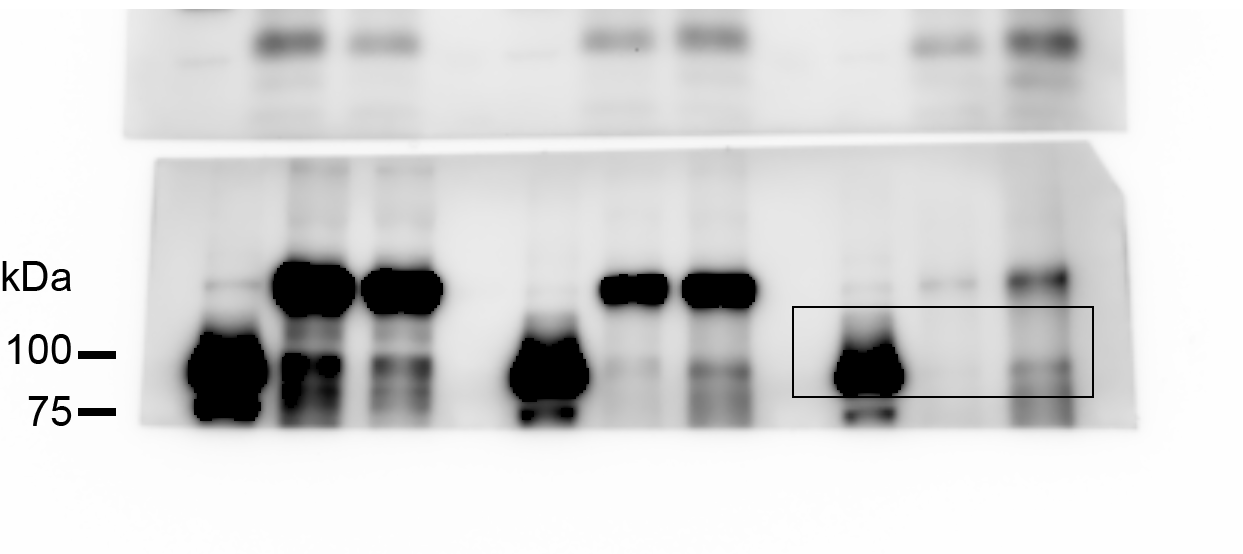

Supplement: Supplementary file 5 — Source data Fig. 3 [file 44318_2025_562_MOESM5_ESM.zip › Figure 3/3C/Western MCM4.tif]

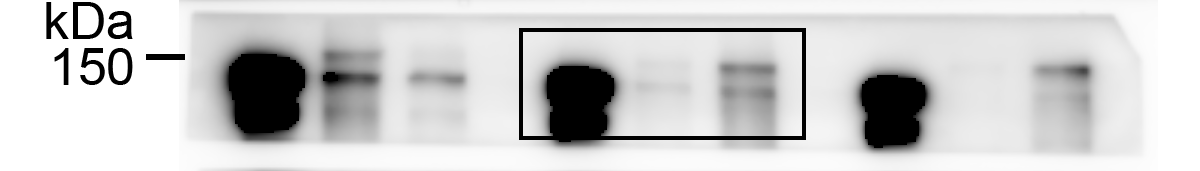

Supplement: Supplementary file 5 — Source data Fig. 3 [file 44318_2025_562_MOESM5_ESM.zip › Figure 3/3C/Western MCM2.tif]

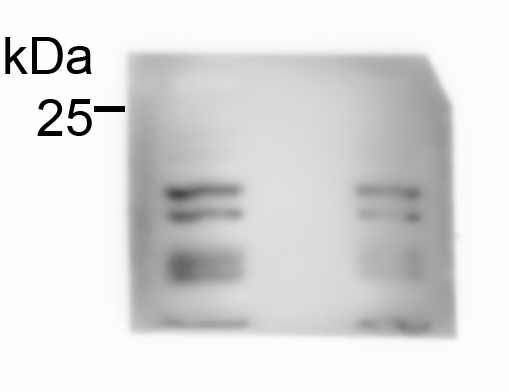

Supplement: Supplementary file 5 — Source data Fig. 3 [file 44318_2025_562_MOESM5_ESM.zip › Figure 3/3C/Western CDC45.tif]

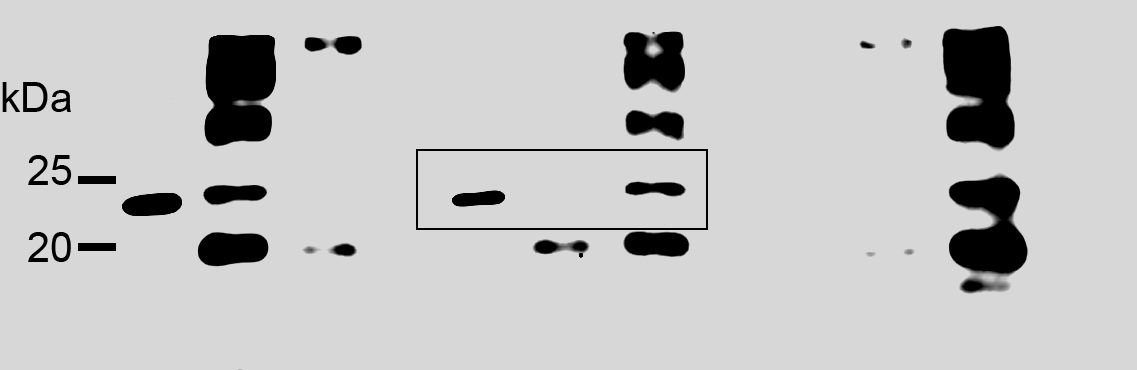

Supplement: Supplementary file 5 — Source data Fig. 3 [file 44318_2025_562_MOESM5_ESM.zip › Figure 3/3C/Western GINS3.tif]

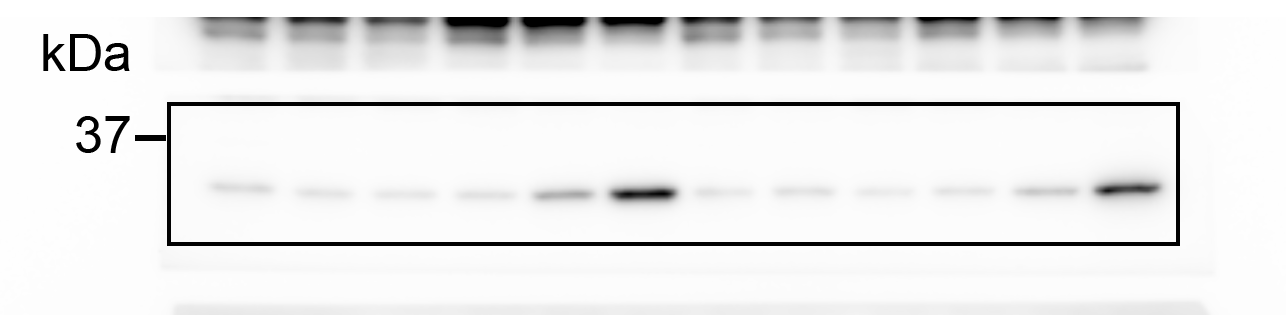

Supplement: Supplementary file 5 — Source data Fig. 3 [file 44318_2025_562_MOESM5_ESM.zip › Figure 3/3H/Western PCNA.tif]

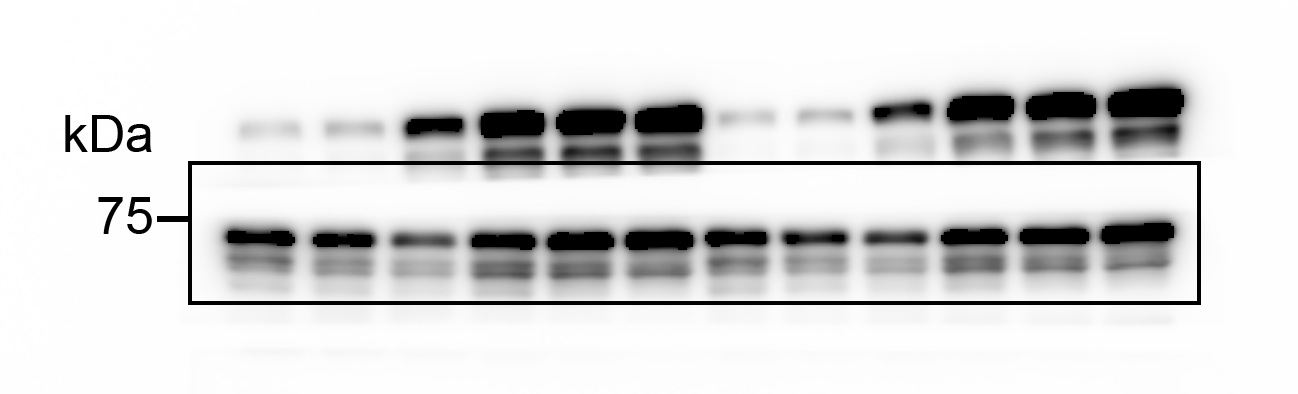

Supplement: Supplementary file 5 — Source data Fig. 3 [file 44318_2025_562_MOESM5_ESM.zip › Figure 3/3H/Western ORC2.tif]

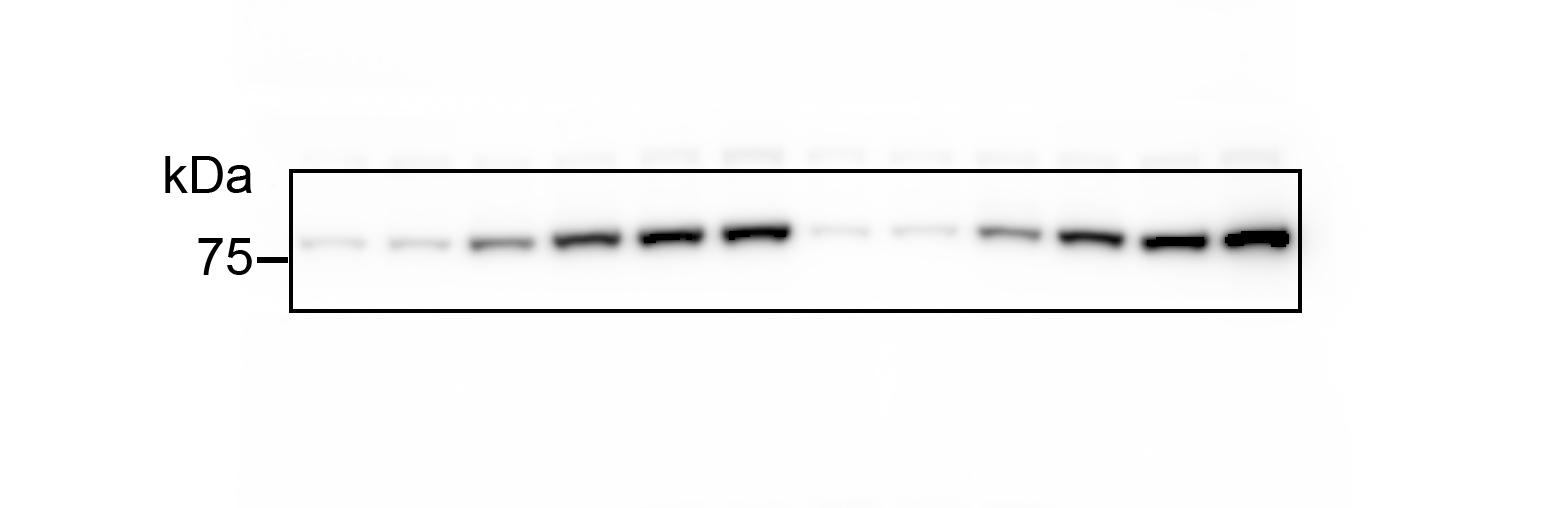

Supplement: Supplementary file 5 — Source data Fig. 3 [file 44318_2025_562_MOESM5_ESM.zip › Figure 3/3H/Western MCM5.tif]

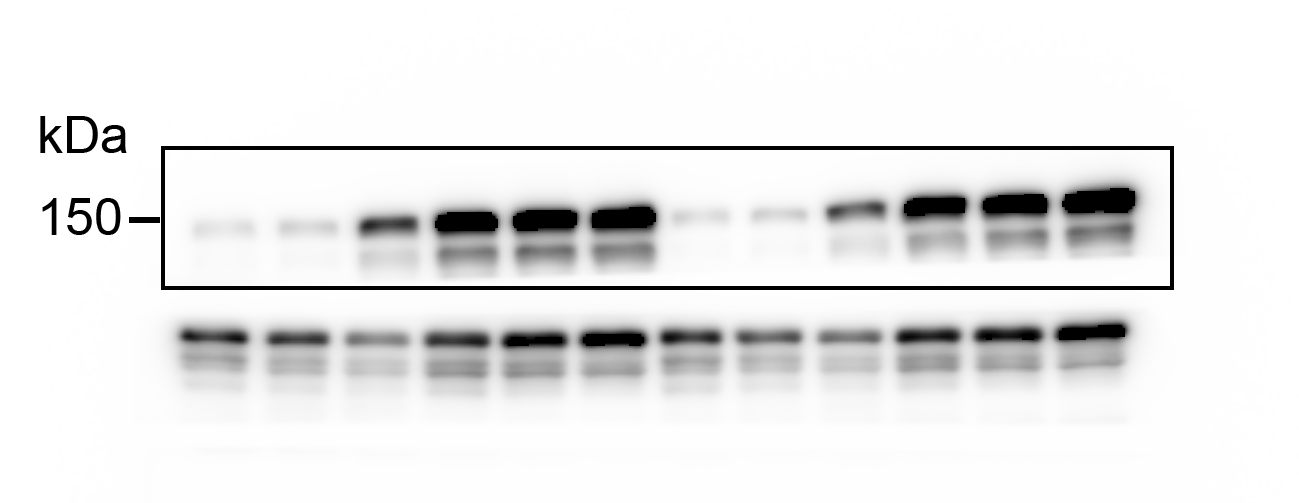

Supplement: Supplementary file 5 — Source data Fig. 3 [file 44318_2025_562_MOESM5_ESM.zip › Figure 3/3H/Western MCM2.tif]

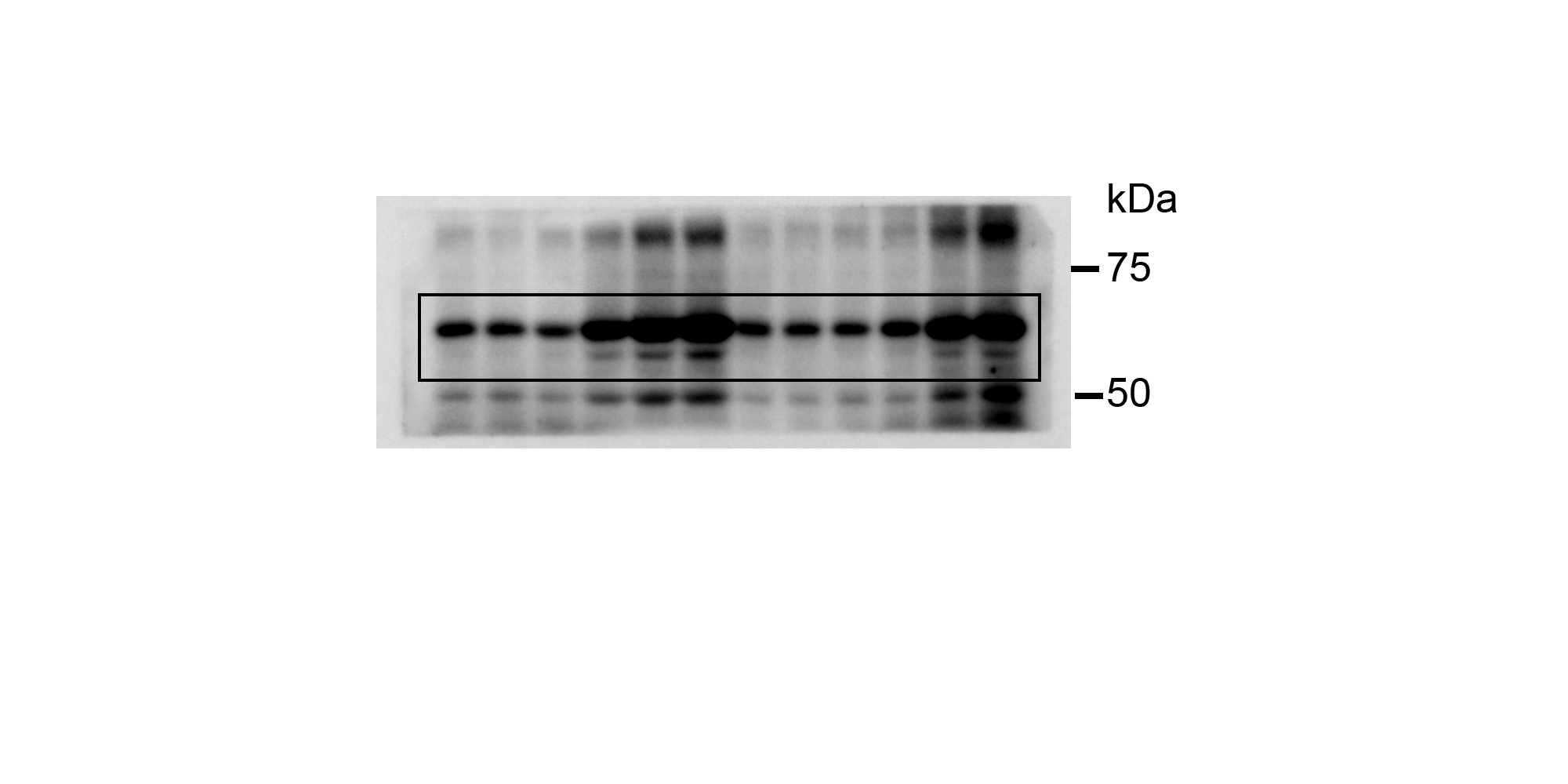

Supplement: Supplementary file 5 — Source data Fig. 3 [file 44318_2025_562_MOESM5_ESM.zip › Figure 3/3H/Western CDC45.tif]

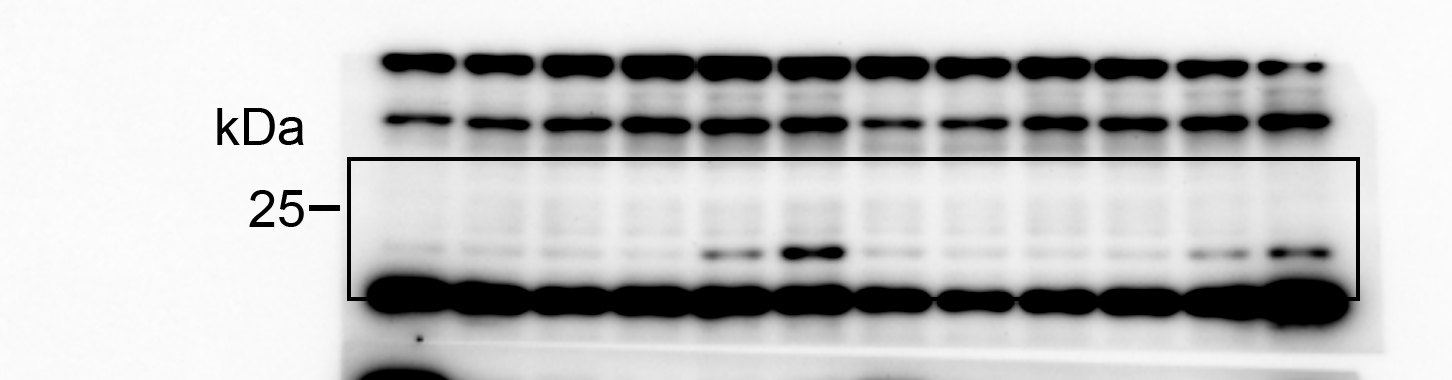

Supplement: Supplementary file 5 — Source data Fig. 3 [file 44318_2025_562_MOESM5_ESM.zip › Figure 3/3H/Western GINS3.tif]

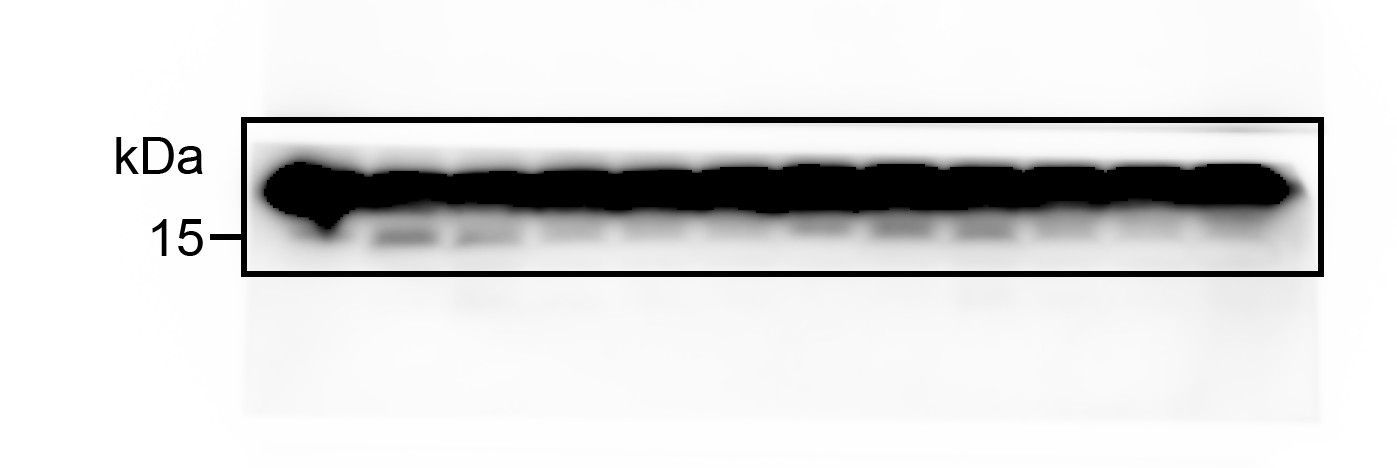

Supplement: Supplementary file 5 — Source data Fig. 3 [file 44318_2025_562_MOESM5_ESM.zip › Figure 3/3H/Western H3.tif]

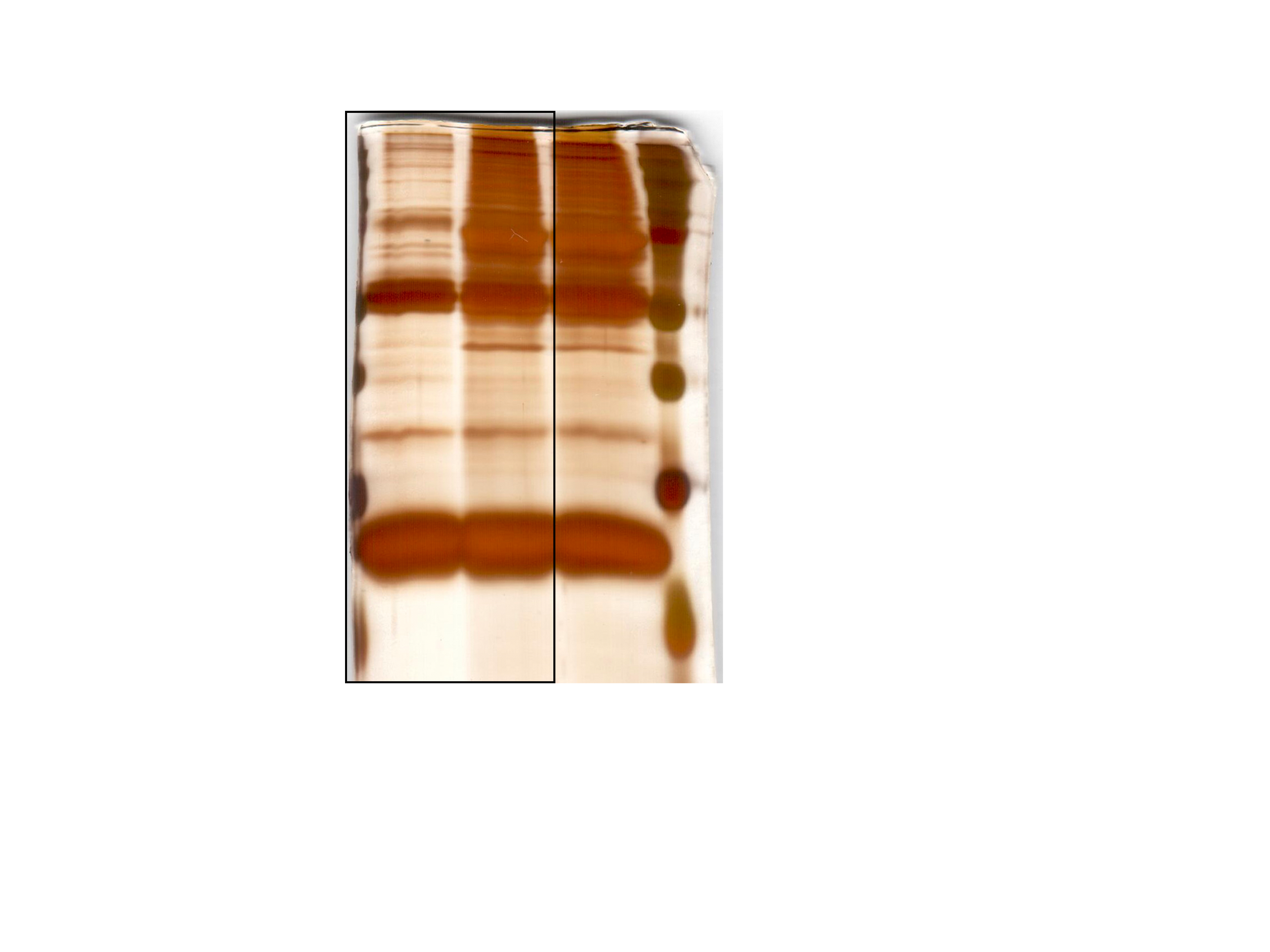

Supplement: Supplementary file 5 — Source data Fig. 3 [file 44318_2025_562_MOESM5_ESM.zip › Figure 3/3A/Western Gel.tif]

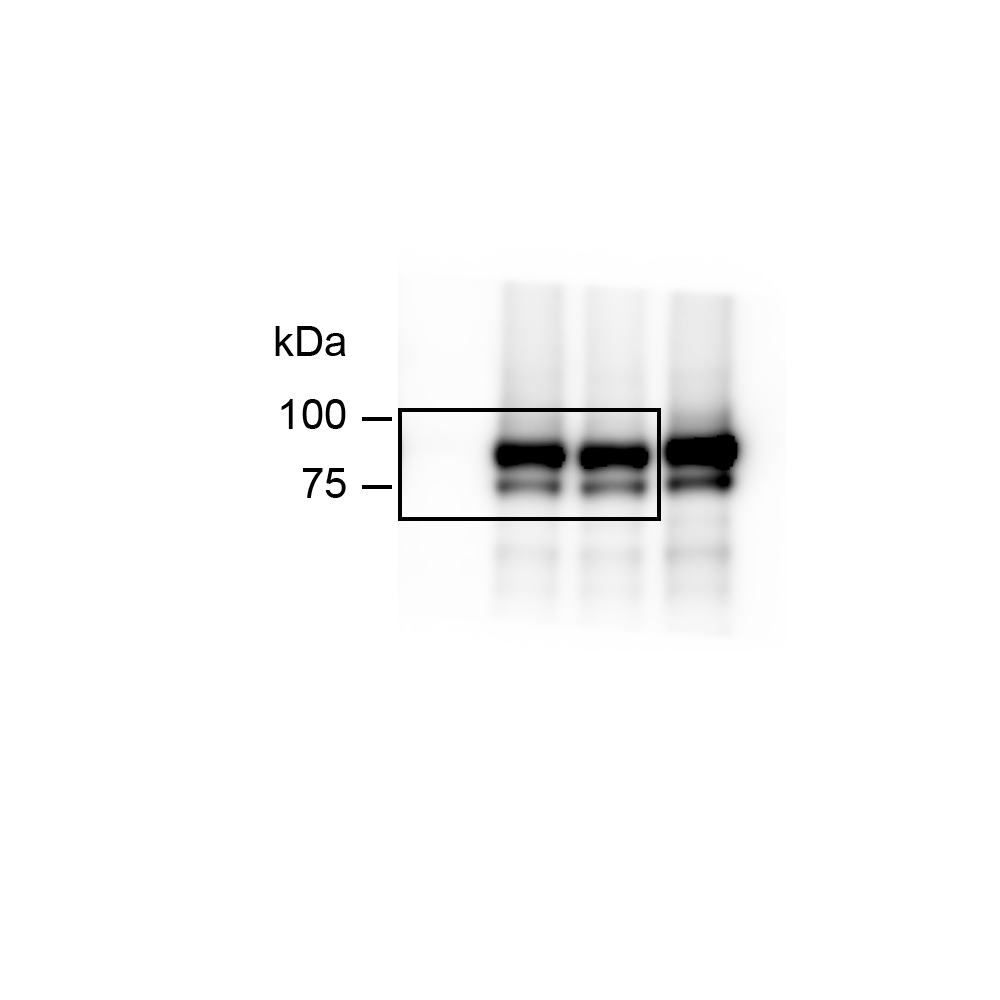

Supplement: Supplementary file 6 — Source data Fig. 4 [file 44318_2025_562_MOESM6_ESM.zip › Figure 4/4E/Western FLAG.tif]

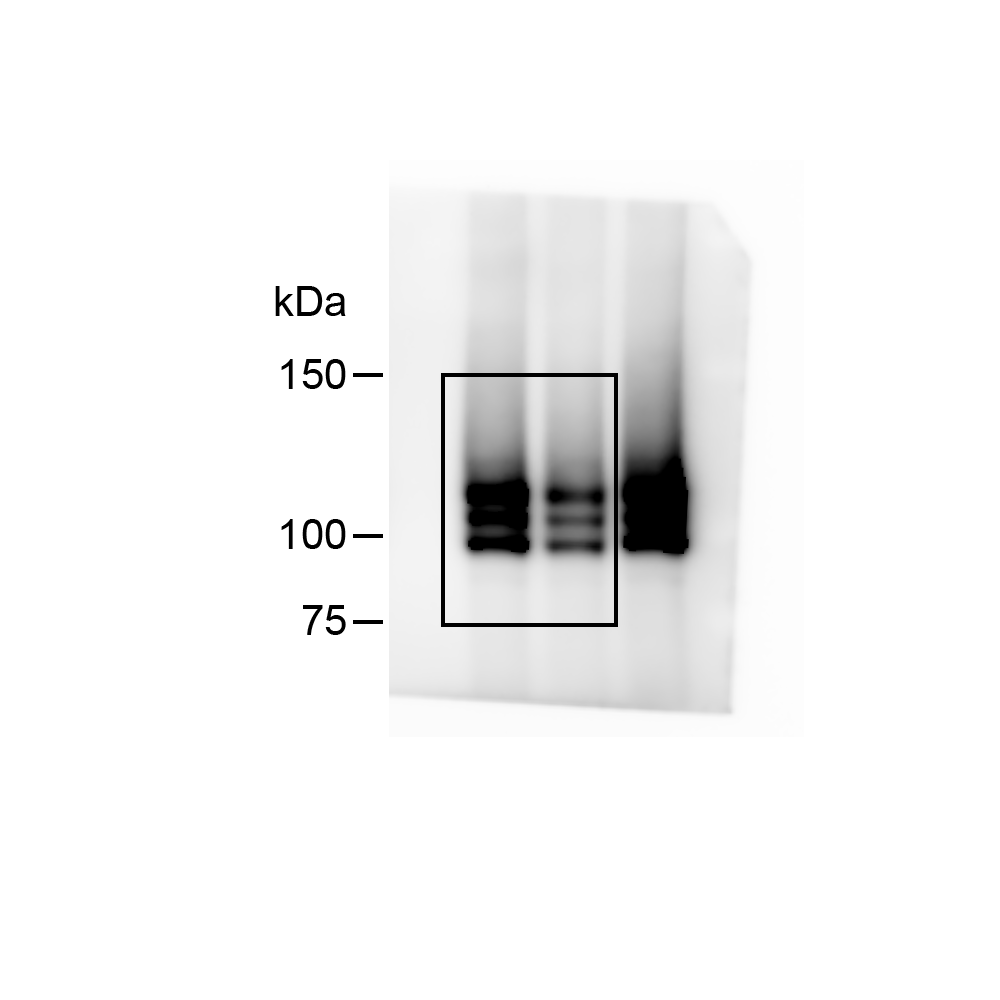

Supplement: Supplementary file 6 — Source data Fig. 4 [file 44318_2025_562_MOESM6_ESM.zip › Figure 4/4E/Western HA.tif]

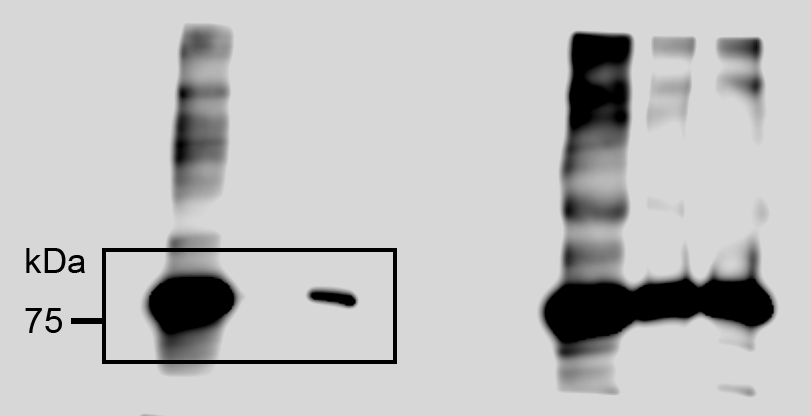

Supplement: Supplementary file 6 — Source data Fig. 4 [file 44318_2025_562_MOESM6_ESM.zip › Figure 4/4B/Western UFL1.tif]

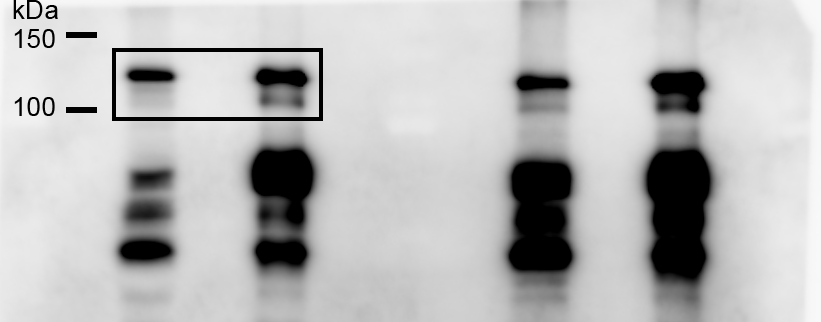

Supplement: Supplementary file 6 — Source data Fig. 4 [file 44318_2025_562_MOESM6_ESM.zip › Figure 4/4B/Western GST.tif]

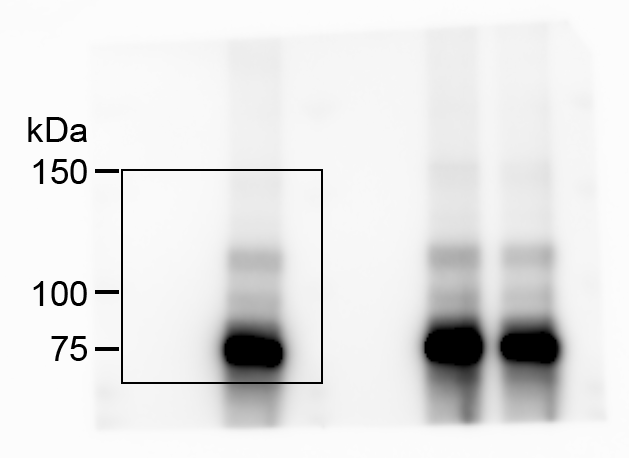

Supplement: Supplementary file 6 — Source data Fig. 4 [file 44318_2025_562_MOESM6_ESM.zip › Figure 4/4C/Western UFM1.tif]

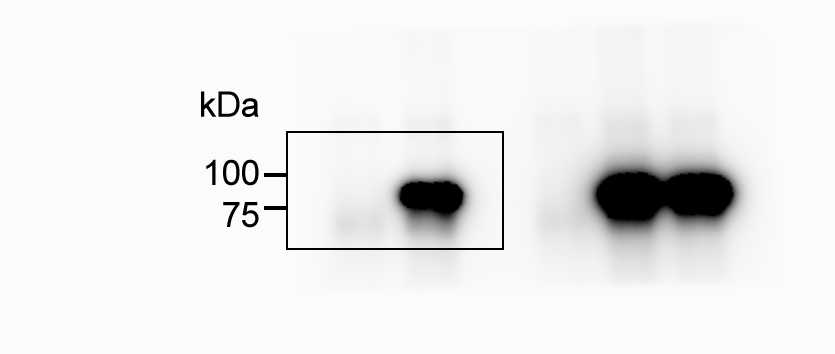

Supplement: Supplementary file 6 — Source data Fig. 4 [file 44318_2025_562_MOESM6_ESM.zip › Figure 4/4C/Western MCM5.tif]

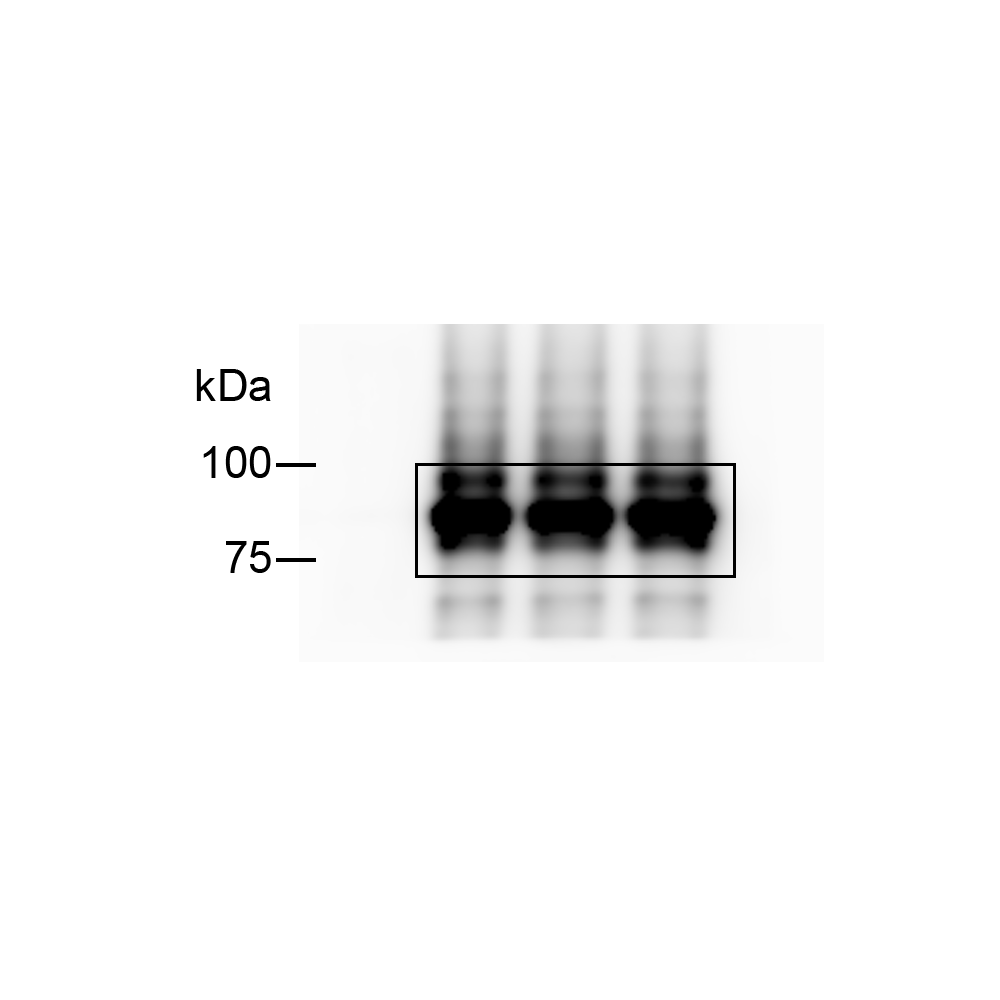

Supplement: Supplementary file 6 — Source data Fig. 4 [file 44318_2025_562_MOESM6_ESM.zip › Figure 4/4D/Western IP FLAG.tif]

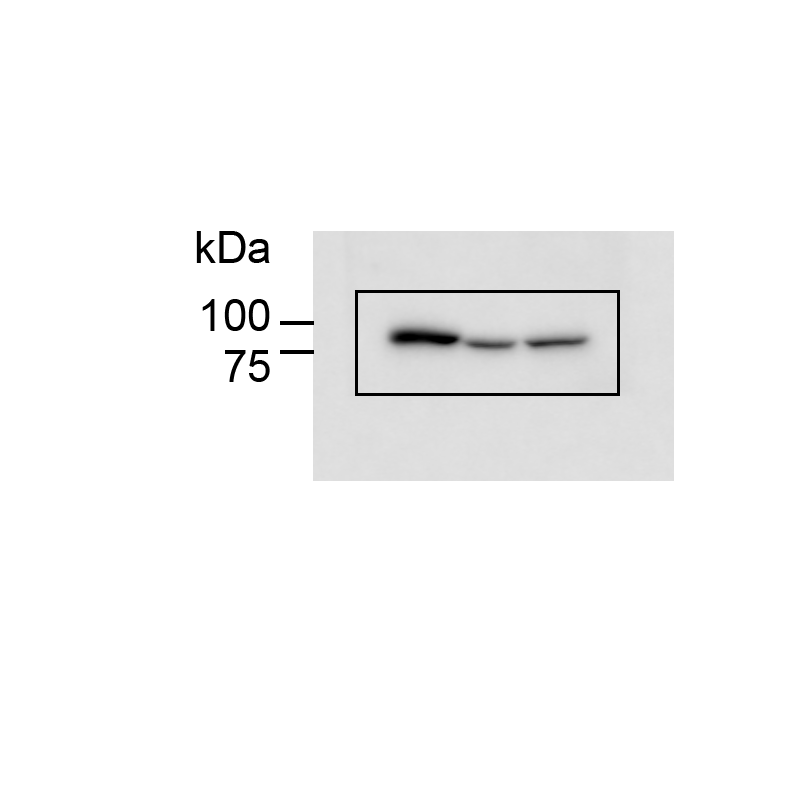

Supplement: Supplementary file 6 — Source data Fig. 4 [file 44318_2025_562_MOESM6_ESM.zip › Figure 4/4D/Western INPUT UFL1.tif]

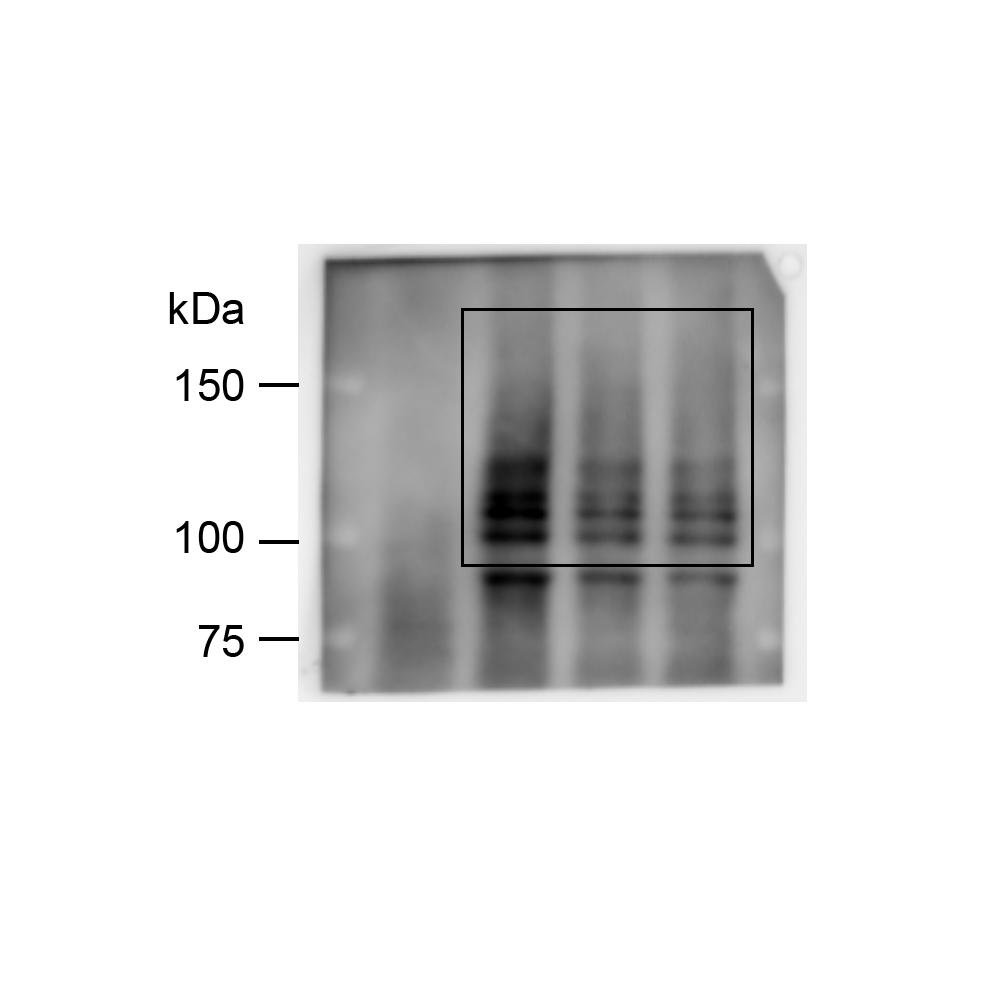

Supplement: Supplementary file 6 — Source data Fig. 4 [file 44318_2025_562_MOESM6_ESM.zip › Figure 4/4D/Western IP HA.tif]

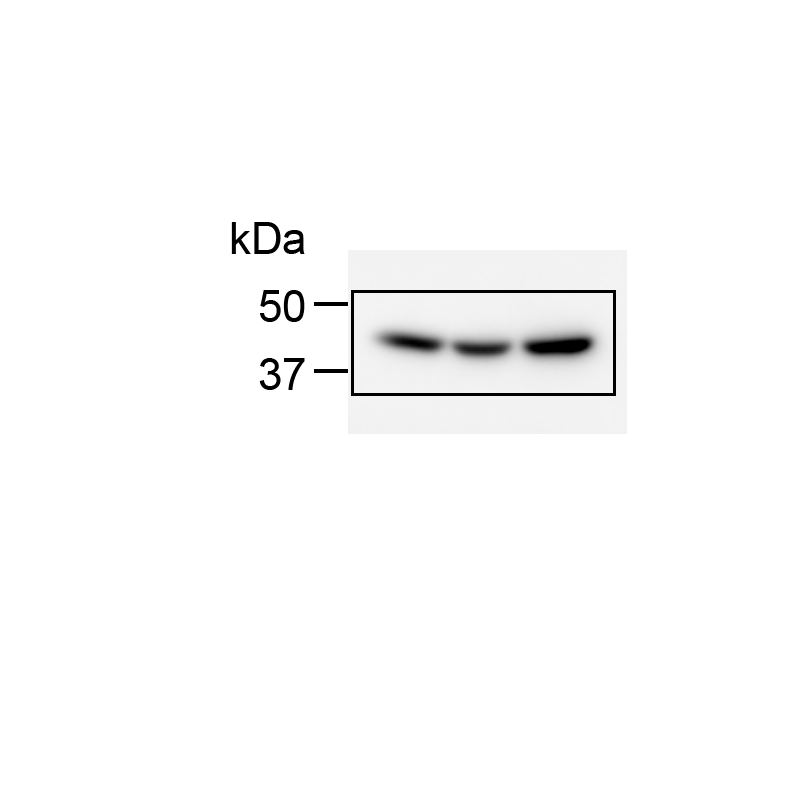

Supplement: Supplementary file 6 — Source data Fig. 4 [file 44318_2025_562_MOESM6_ESM.zip › Figure 4/4D/Western INPUT Actin.tif]

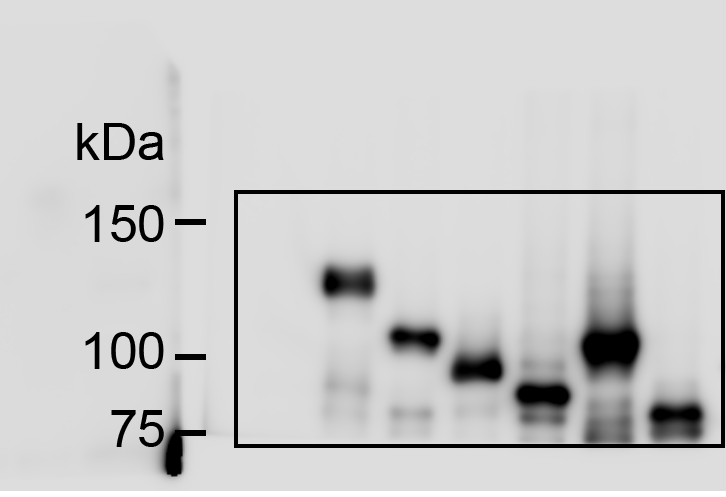

Supplement: Supplementary file 6 — Source data Fig. 4 [file 44318_2025_562_MOESM6_ESM.zip › Figure 4/4A/Western FLAG.tif]

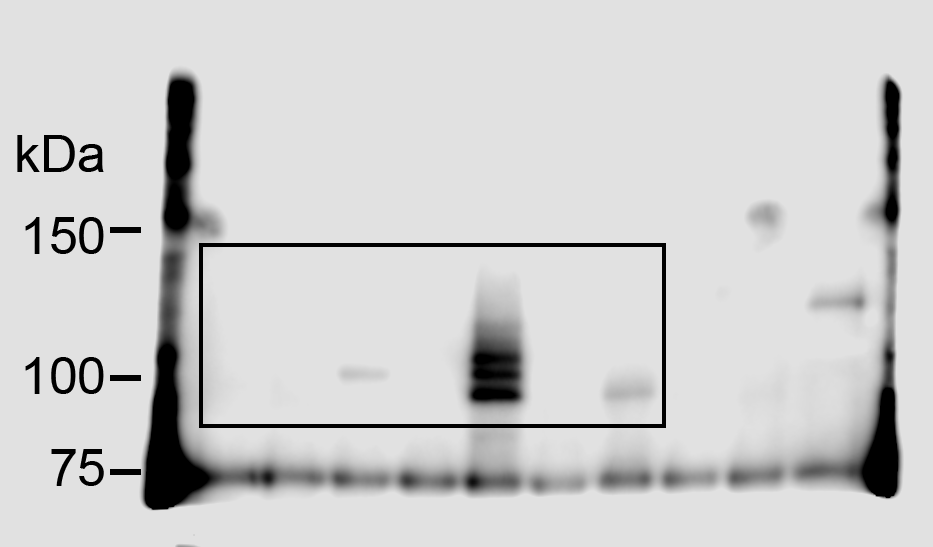

Supplement: Supplementary file 6 — Source data Fig. 4 [file 44318_2025_562_MOESM6_ESM.zip › Figure 4/4A/Western HA.tif]

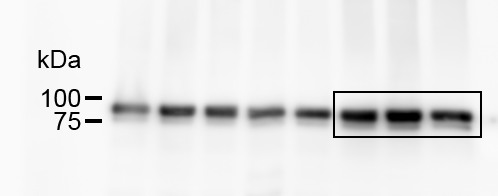

Supplement: Supplementary file 6 — Source data Fig. 4 [file 44318_2025_562_MOESM6_ESM.zip › Figure 4/4F/Western IP FLAG.tif]

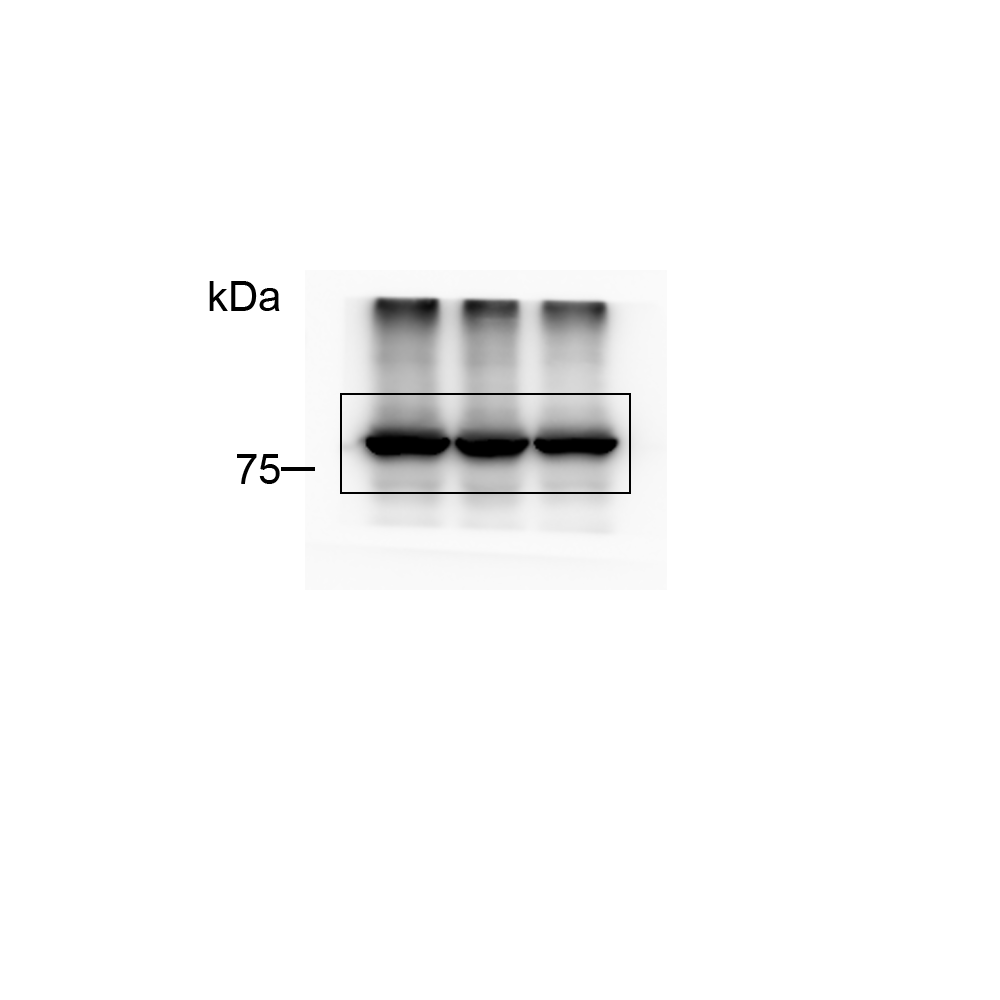

Supplement: Supplementary file 6 — Source data Fig. 4 [file 44318_2025_562_MOESM6_ESM.zip › Figure 4/4F/Western INPUT FLAG.tif]

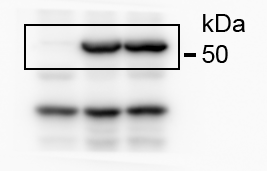

Supplement: Supplementary file 6 — Source data Fig. 4 [file 44318_2025_562_MOESM6_ESM.zip › Figure 4/4F/Western INPUT MYC.tif]

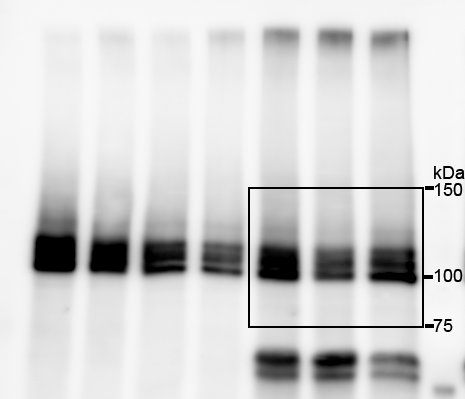

Supplement: Supplementary file 6 — Source data Fig. 4 [file 44318_2025_562_MOESM6_ESM.zip › Figure 4/4F/Western IP HA.tif]

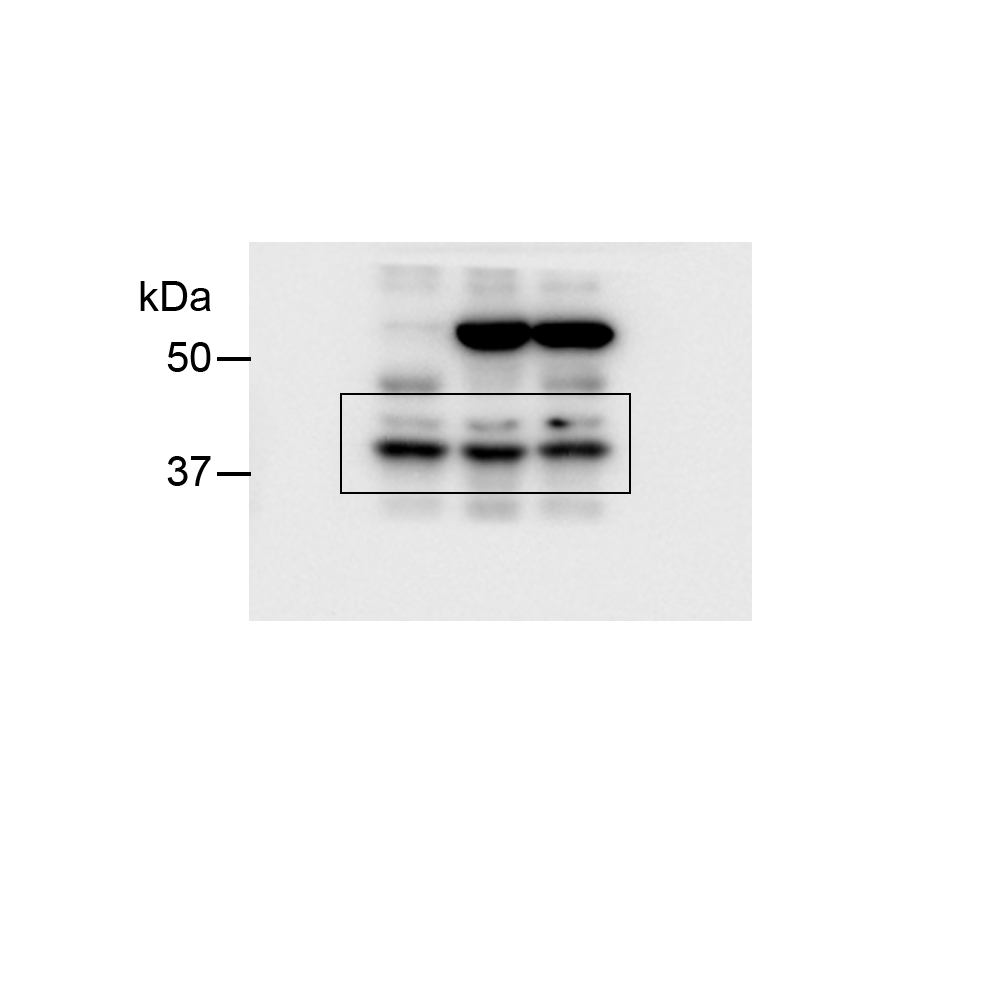

Supplement: Supplementary file 6 — Source data Fig. 4 [file 44318_2025_562_MOESM6_ESM.zip › Figure 4/4F/Western INPUT Actin.tif]

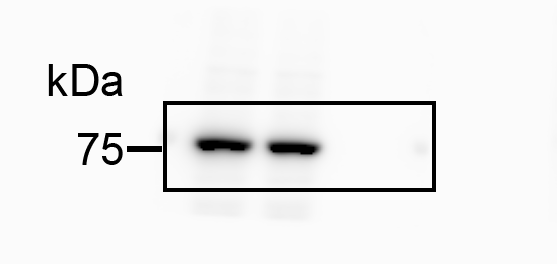

Supplement: Supplementary file 7 — Source data Fig. 5 [file 44318_2025_562_MOESM7_ESM.zip › Figure 5/5A/Western MCM7.tif]

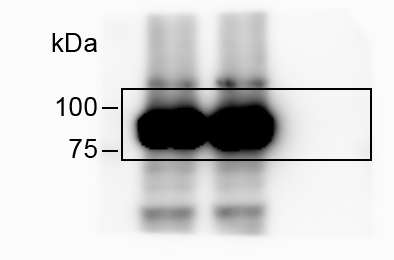

Supplement: Supplementary file 7 — Source data Fig. 5 [file 44318_2025_562_MOESM7_ESM.zip › Figure 5/5A/Western MCM5.tif]

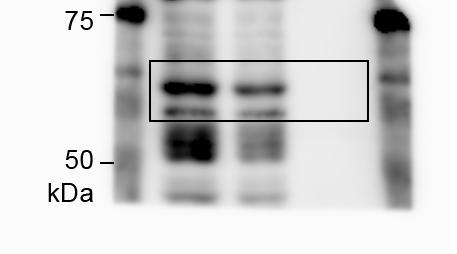

Supplement: Supplementary file 7 — Source data Fig. 5 [file 44318_2025_562_MOESM7_ESM.zip › Figure 5/5A/Western CDC45.tif]

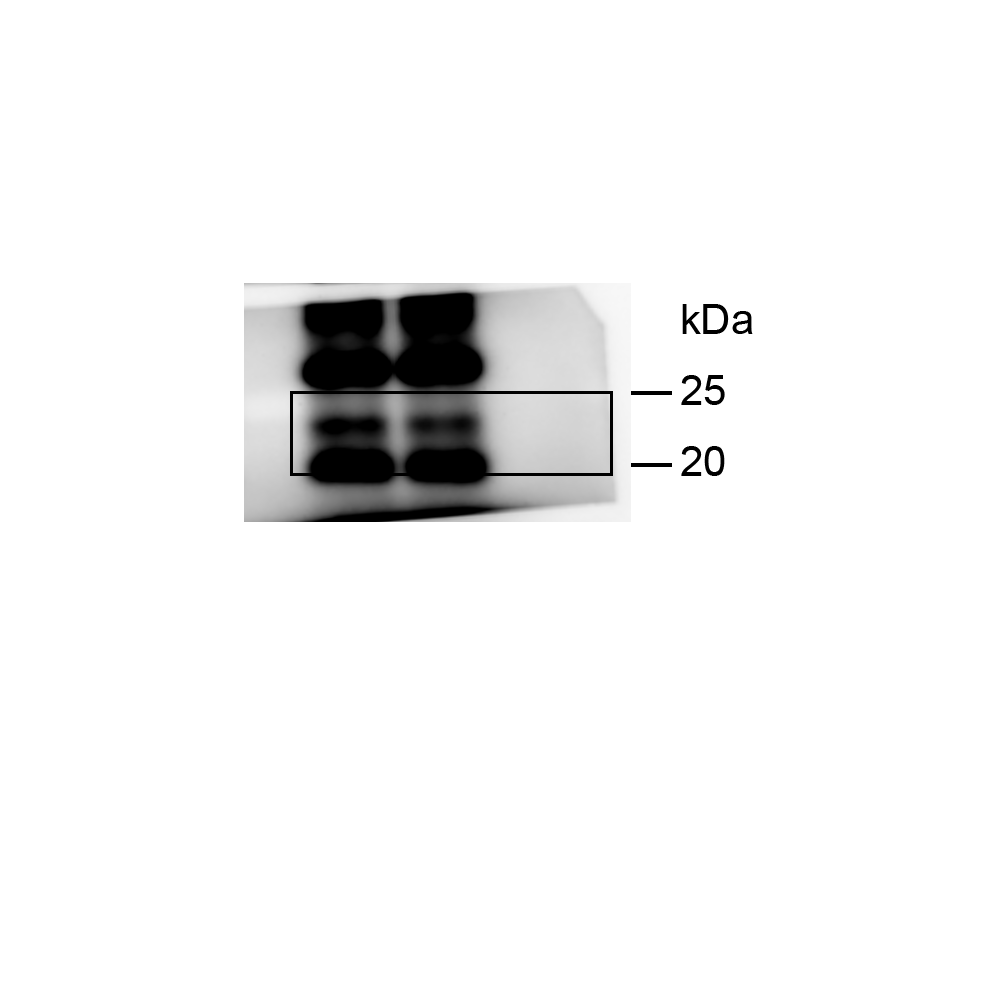

Supplement: Supplementary file 7 — Source data Fig. 5 [file 44318_2025_562_MOESM7_ESM.zip › Figure 5/5A/Western GINS3.tif]

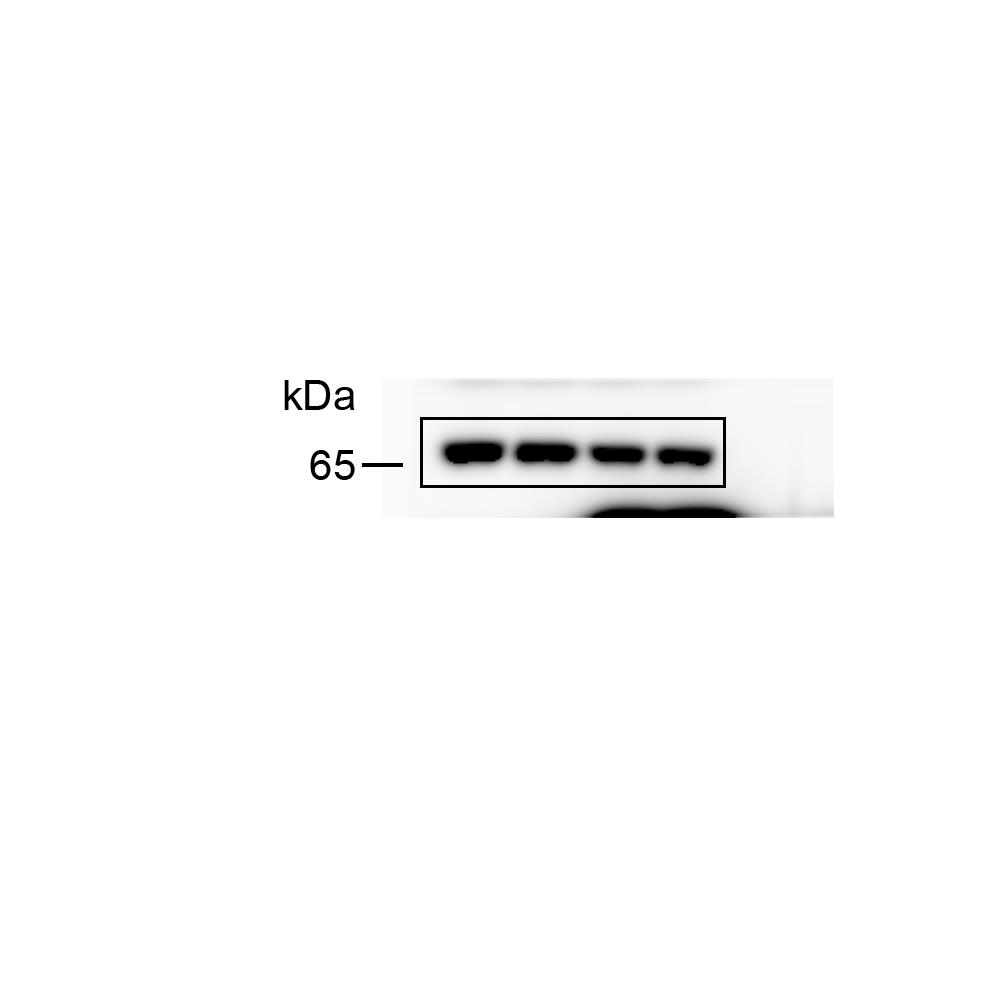

Supplement: Supplementary file 7 — Source data Fig. 5 [file 44318_2025_562_MOESM7_ESM.zip › Figure 5/5B/Western INPUT CDC45.tif]

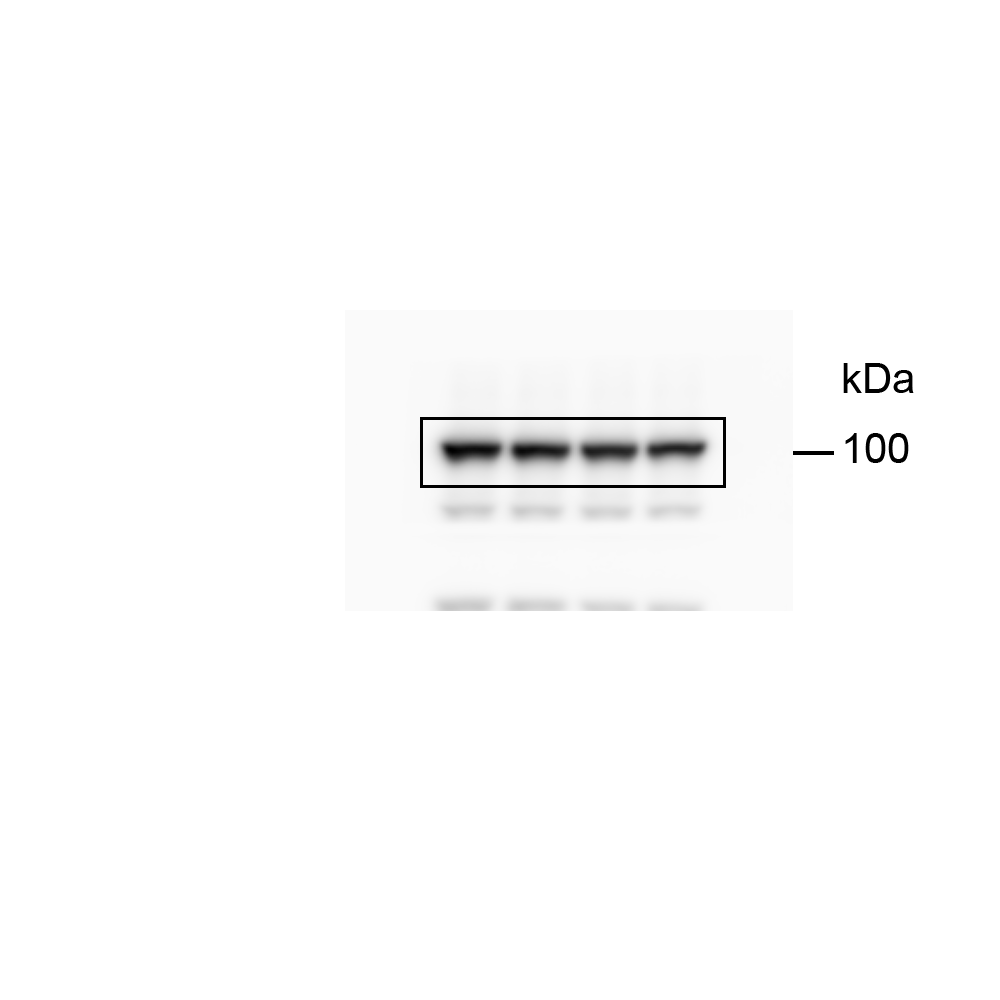

Supplement: Supplementary file 7 — Source data Fig. 5 [file 44318_2025_562_MOESM7_ESM.zip › Figure 5/5B/Western INPUT MCM3.tif]

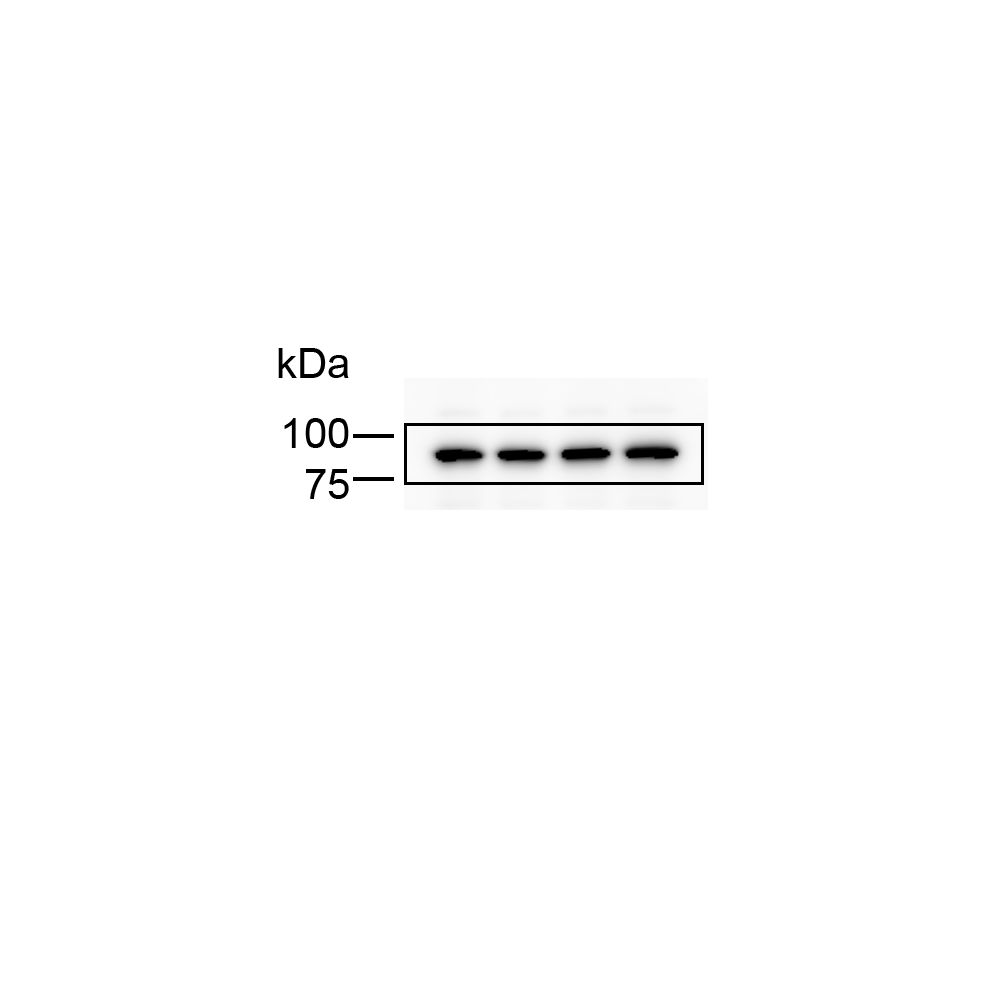

Supplement: Supplementary file 7 — Source data Fig. 5 [file 44318_2025_562_MOESM7_ESM.zip › Figure 5/5B/Western INPUT MCM5.tif]

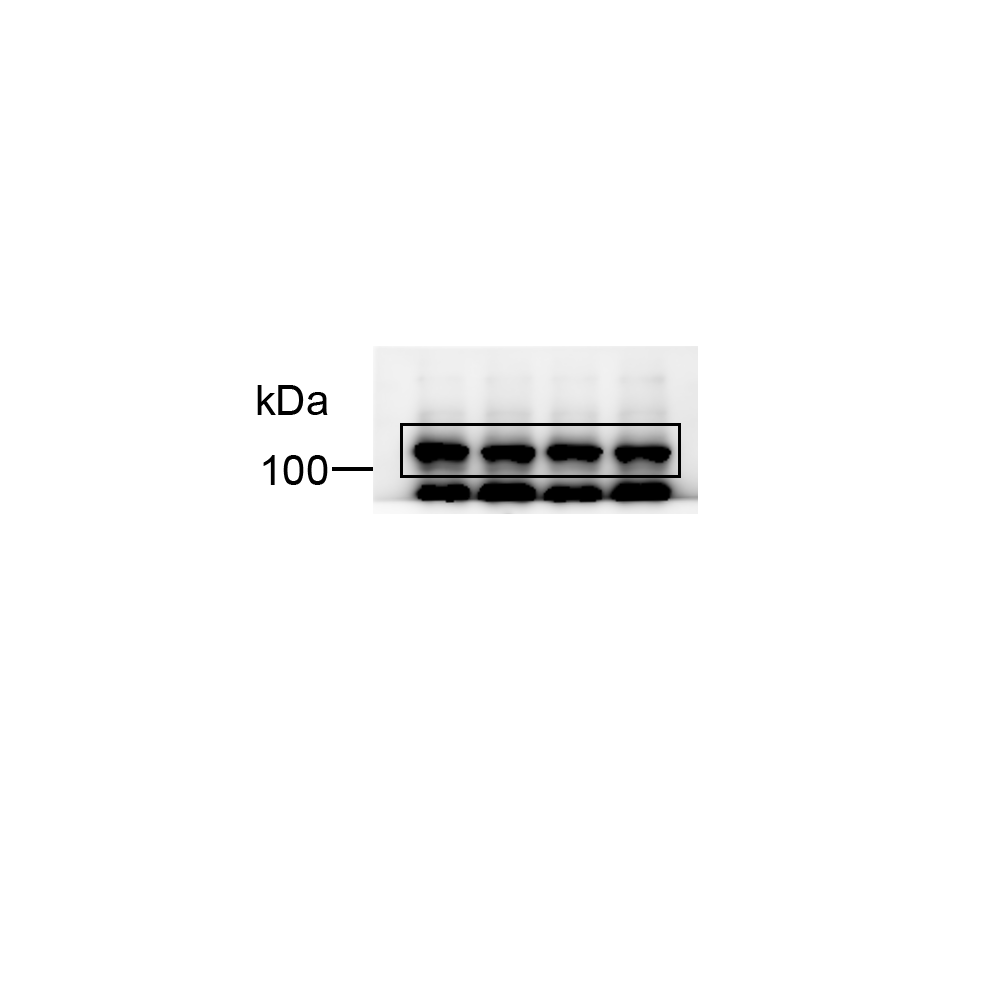

Supplement: Supplementary file 7 — Source data Fig. 5 [file 44318_2025_562_MOESM7_ESM.zip › Figure 5/5B/Western INPUT MCM10.tif]

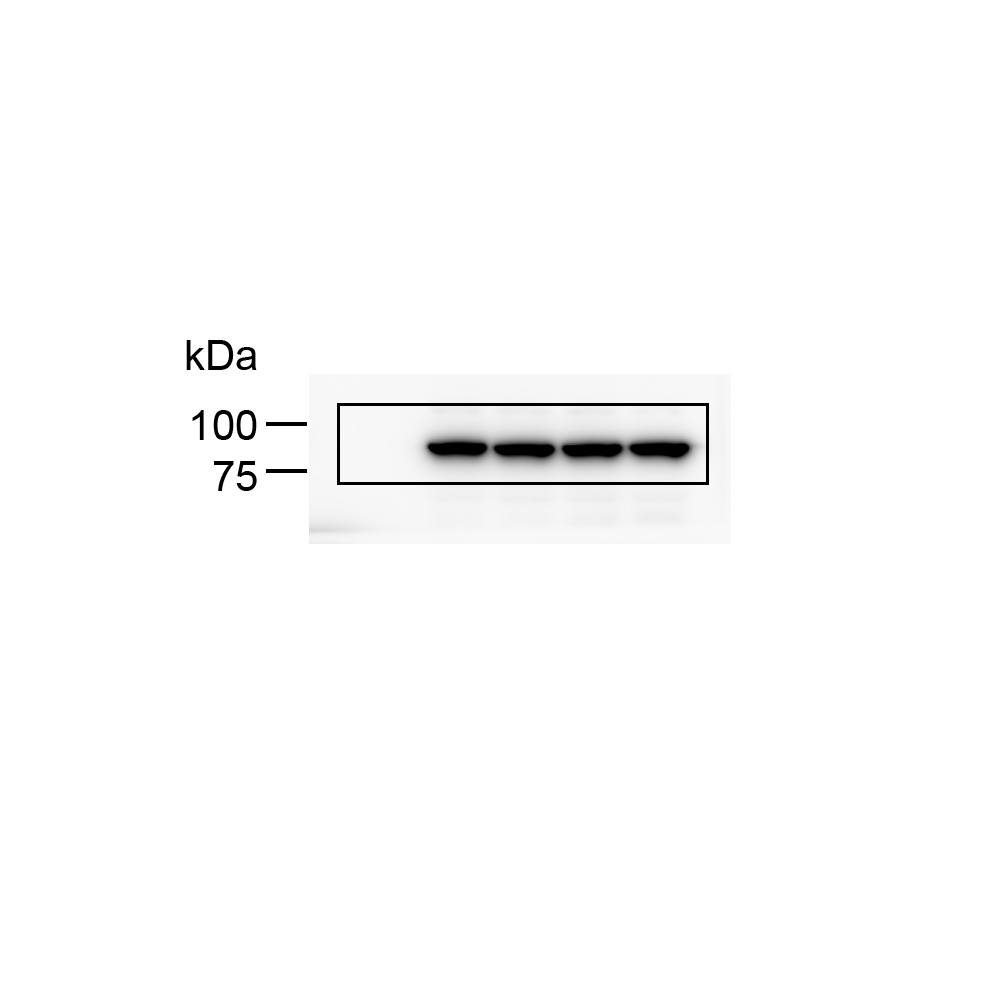

Supplement: Supplementary file 7 — Source data Fig. 5 [file 44318_2025_562_MOESM7_ESM.zip › Figure 5/5B/Western IP MCM5.tif]

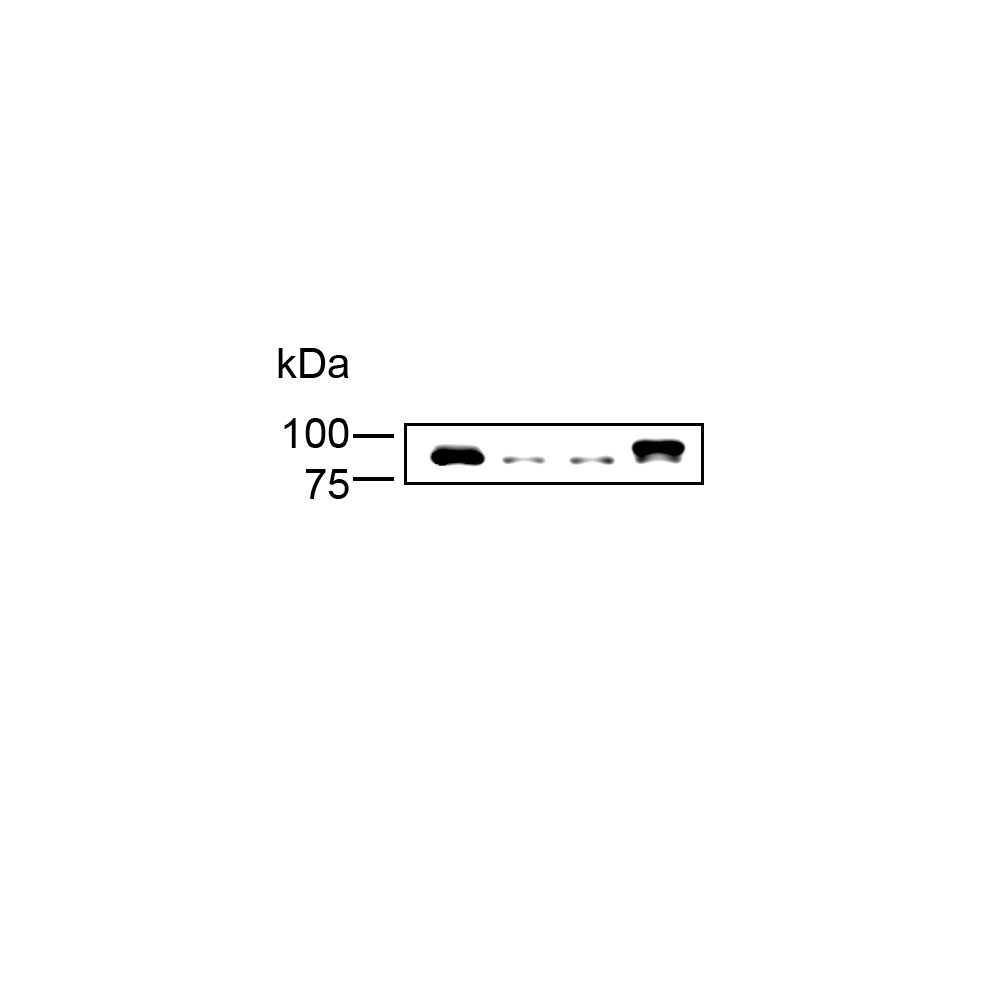

Supplement: Supplementary file 7 — Source data Fig. 5 [file 44318_2025_562_MOESM7_ESM.zip › Figure 5/5B/Western INPUT UFL1.tif]

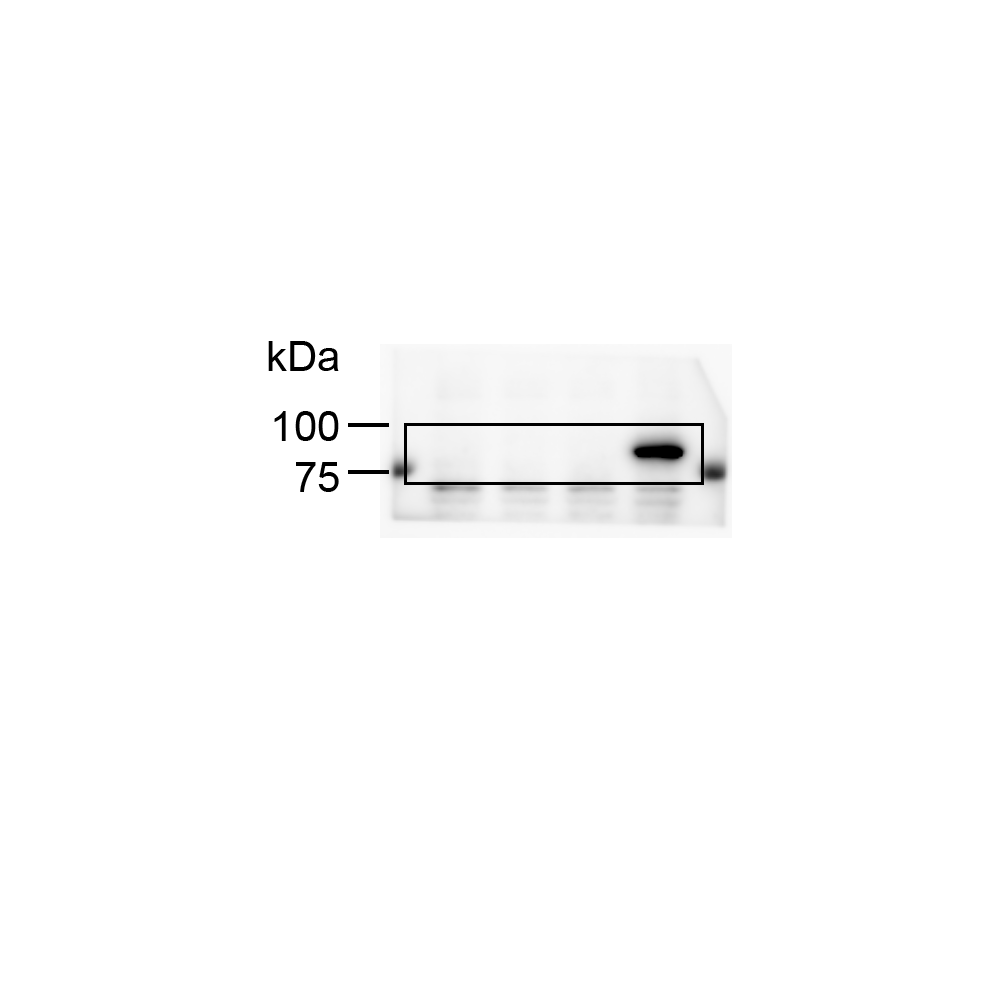

Supplement: Supplementary file 7 — Source data Fig. 5 [file 44318_2025_562_MOESM7_ESM.zip › Figure 5/5B/Western INPUT HA.tif]

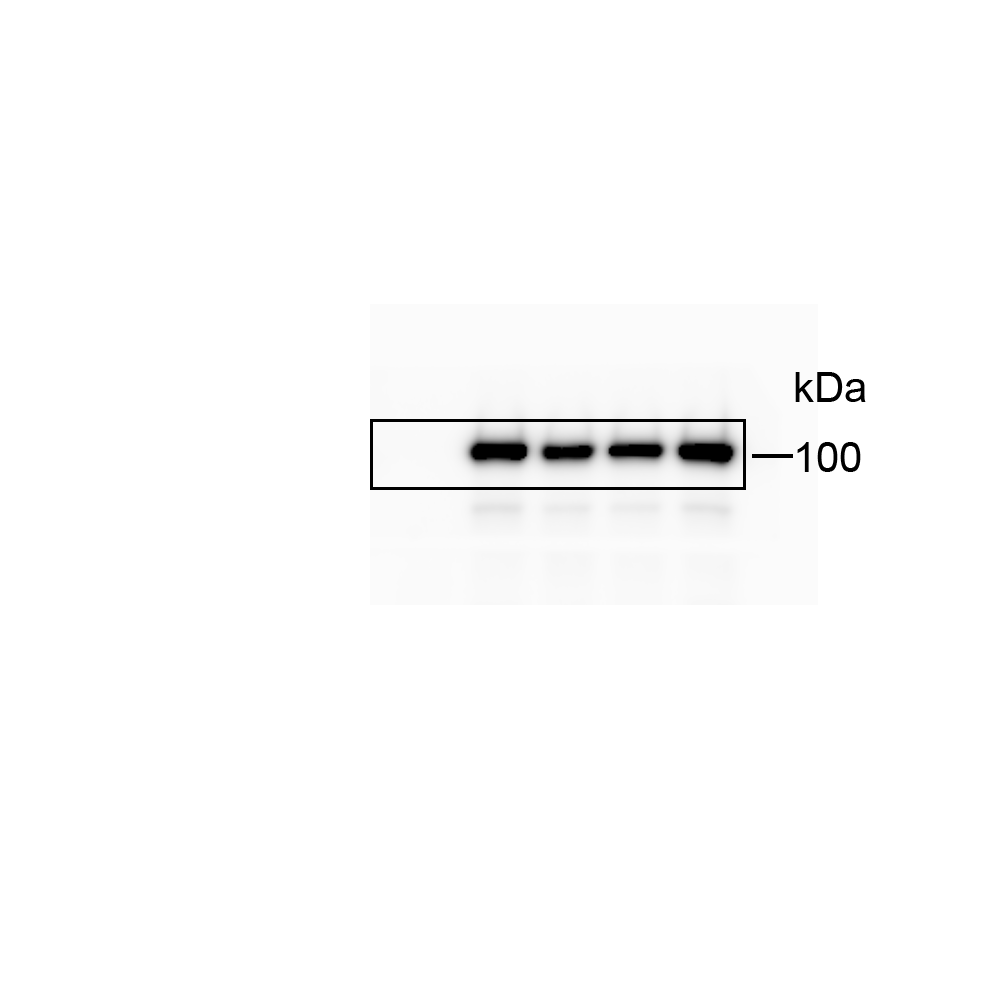

Supplement: Supplementary file 7 — Source data Fig. 5 [file 44318_2025_562_MOESM7_ESM.zip › Figure 5/5B/Western IP MCM3.tif]

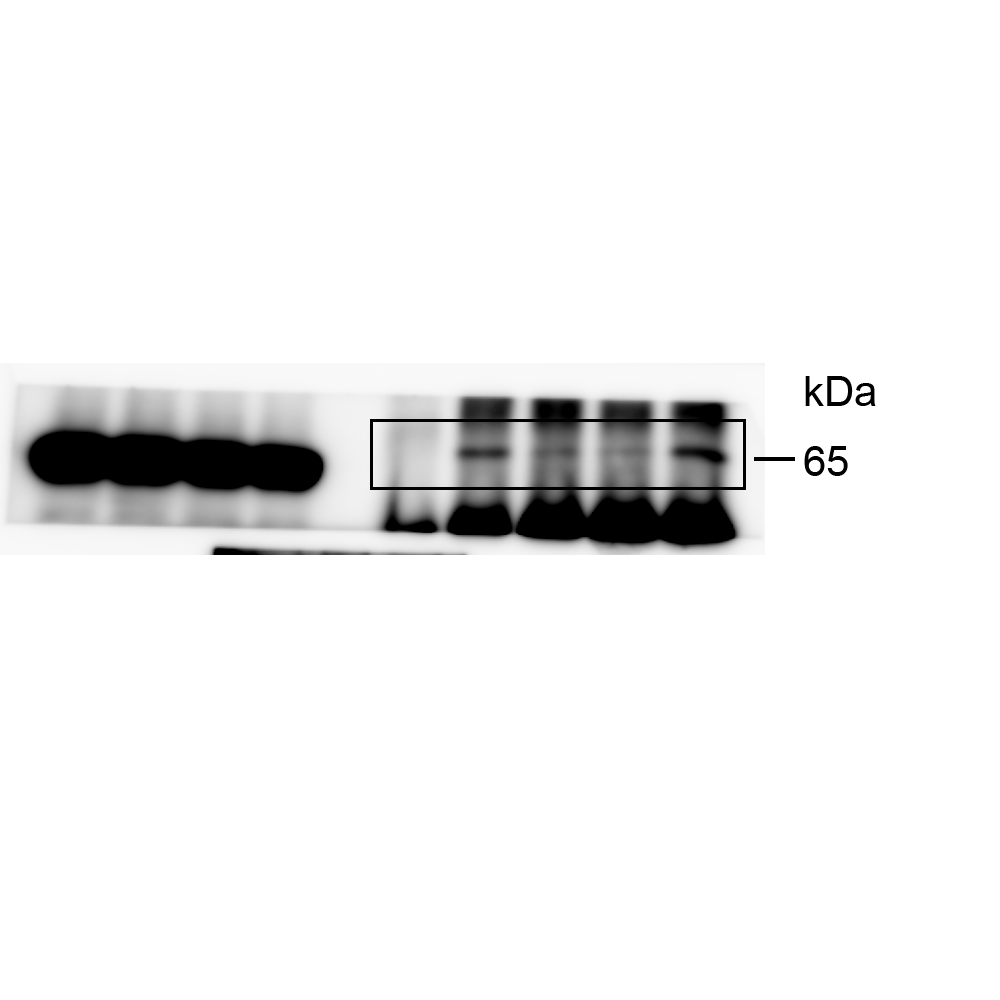

Supplement: Supplementary file 7 — Source data Fig. 5 [file 44318_2025_562_MOESM7_ESM.zip › Figure 5/5B/Western IP CDC45.tif]

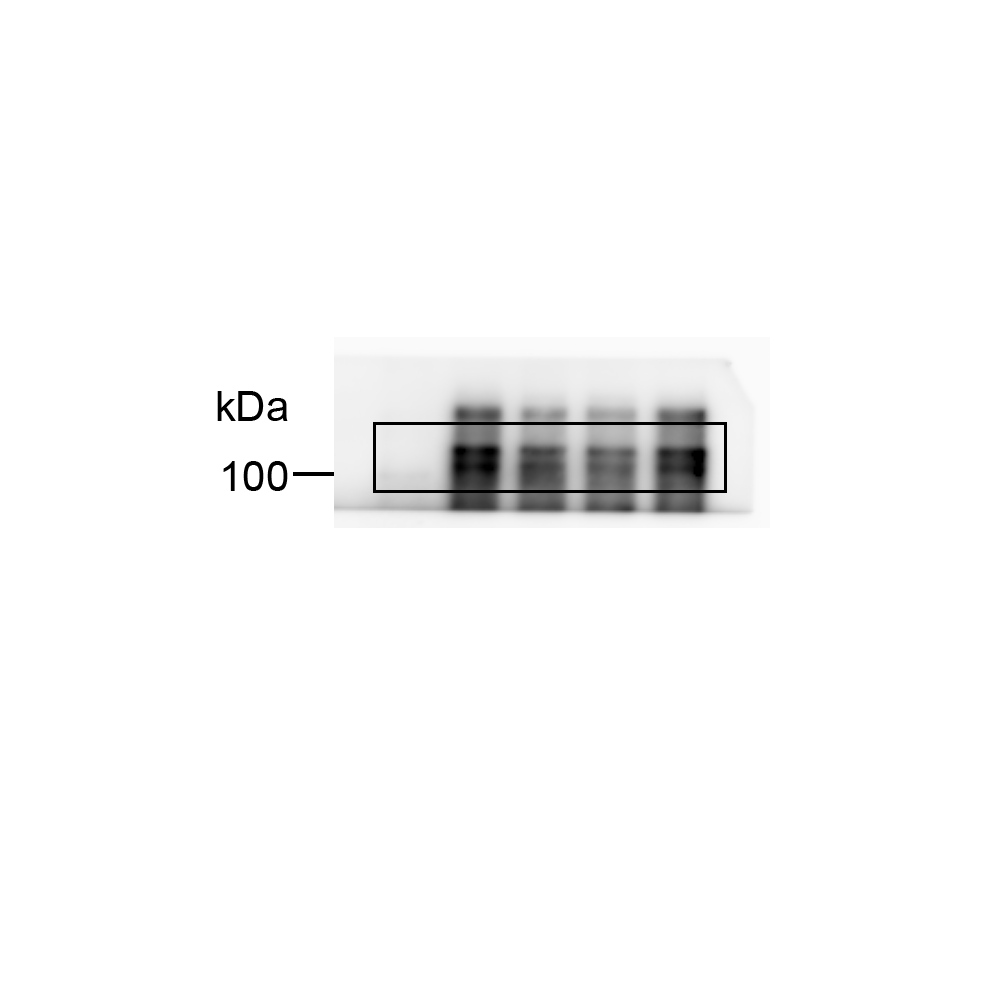

Supplement: Supplementary file 7 — Source data Fig. 5 [file 44318_2025_562_MOESM7_ESM.zip › Figure 5/5B/Western IP MCM10.tif]

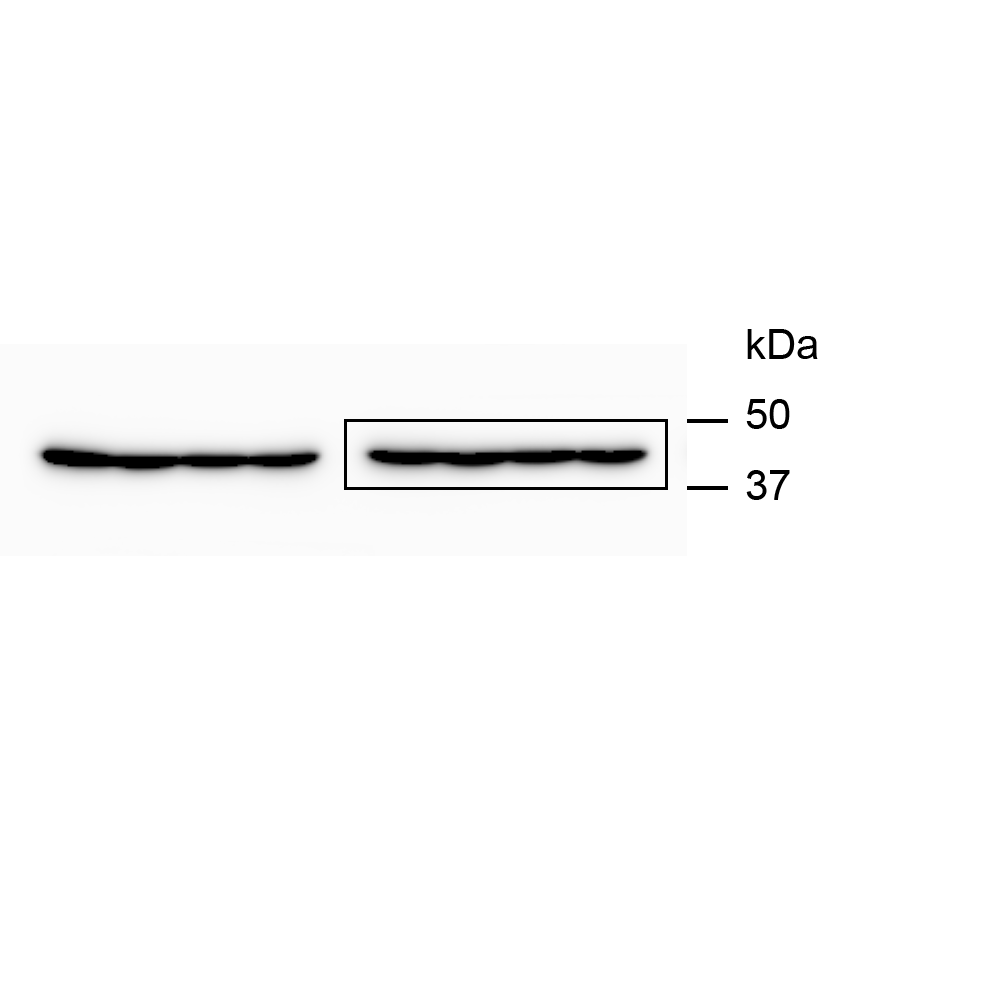

Supplement: Supplementary file 7 — Source data Fig. 5 [file 44318_2025_562_MOESM7_ESM.zip › Figure 5/5B/Western INPUT Actin.tif]

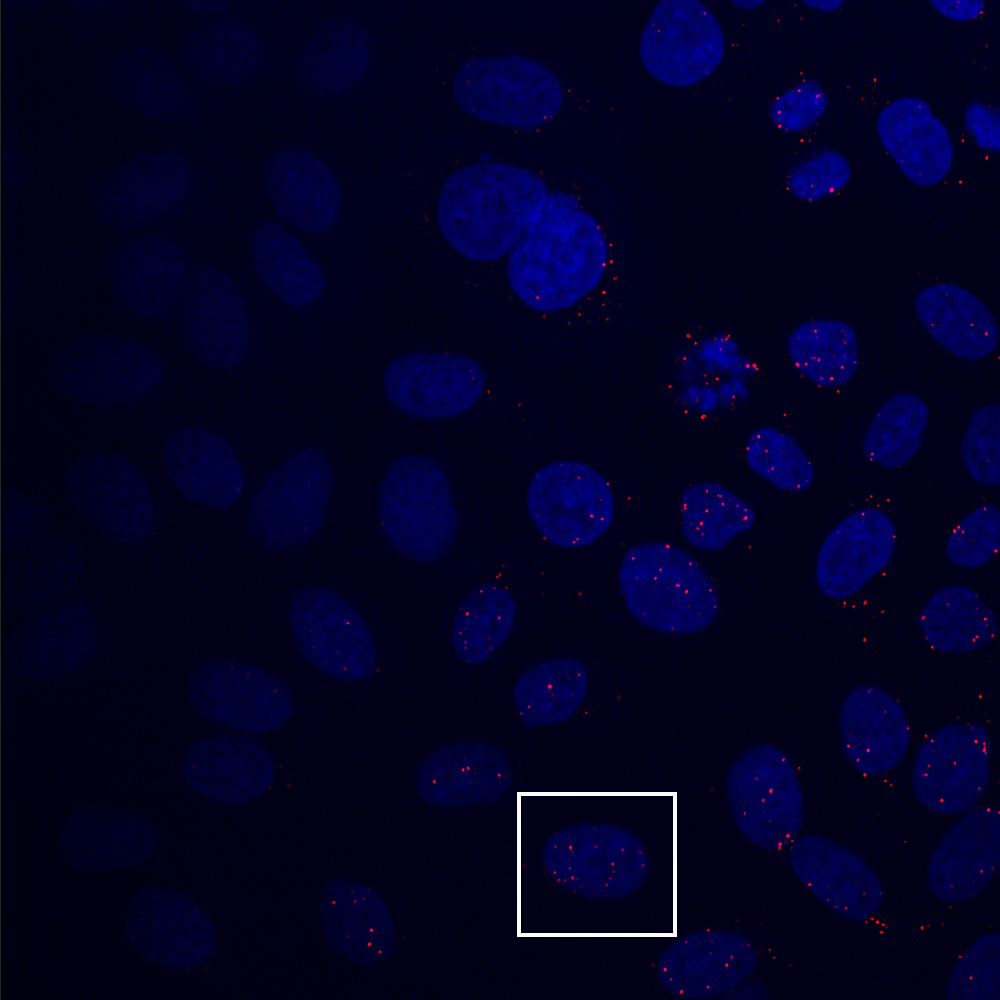

Supplement: Supplementary file 7 — Source data Fig. 5 [file 44318_2025_562_MOESM7_ESM.zip › Figure 5/5C/MCM5 CDC45 PLA/PLA immunostaining DMSO.tif]

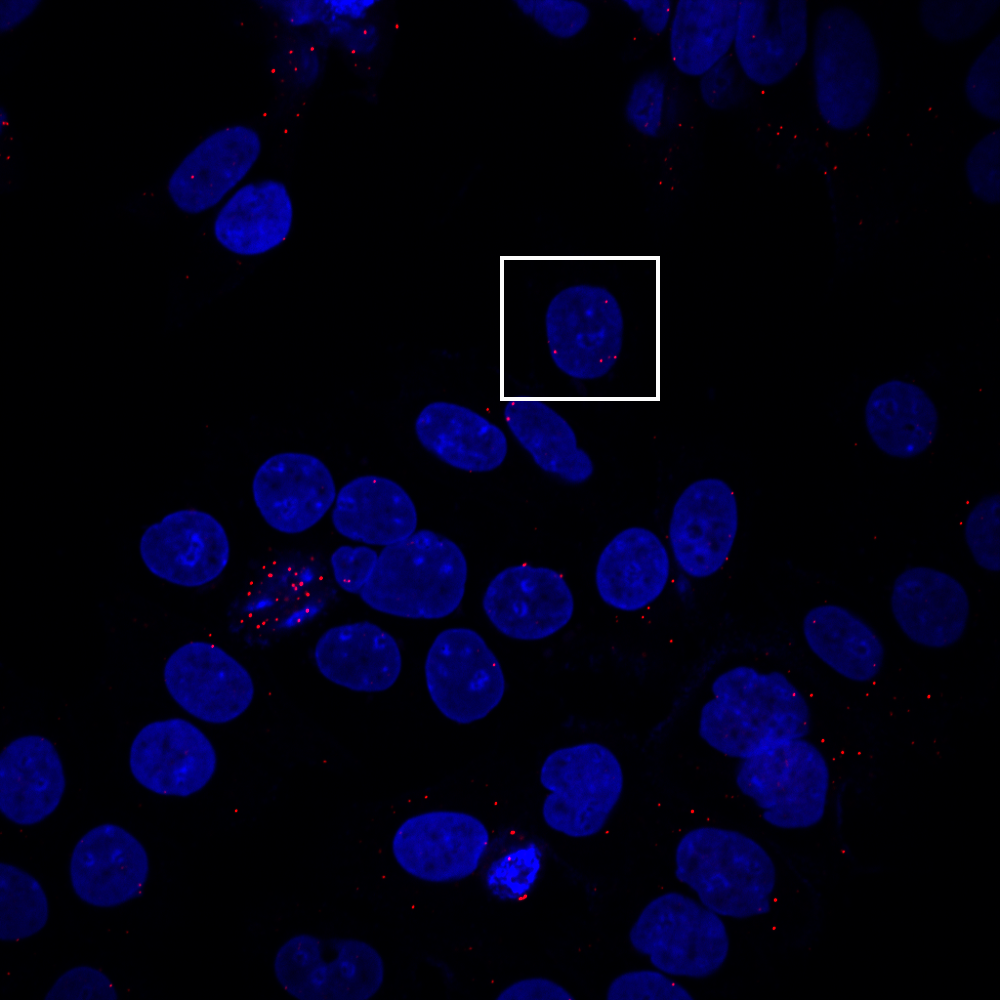

Supplement: Supplementary file 7 — Source data Fig. 5 [file 44318_2025_562_MOESM7_ESM.zip › Figure 5/5C/MCM5 CDC45 PLA/PLA immunostaining DKM 2-93.tif]

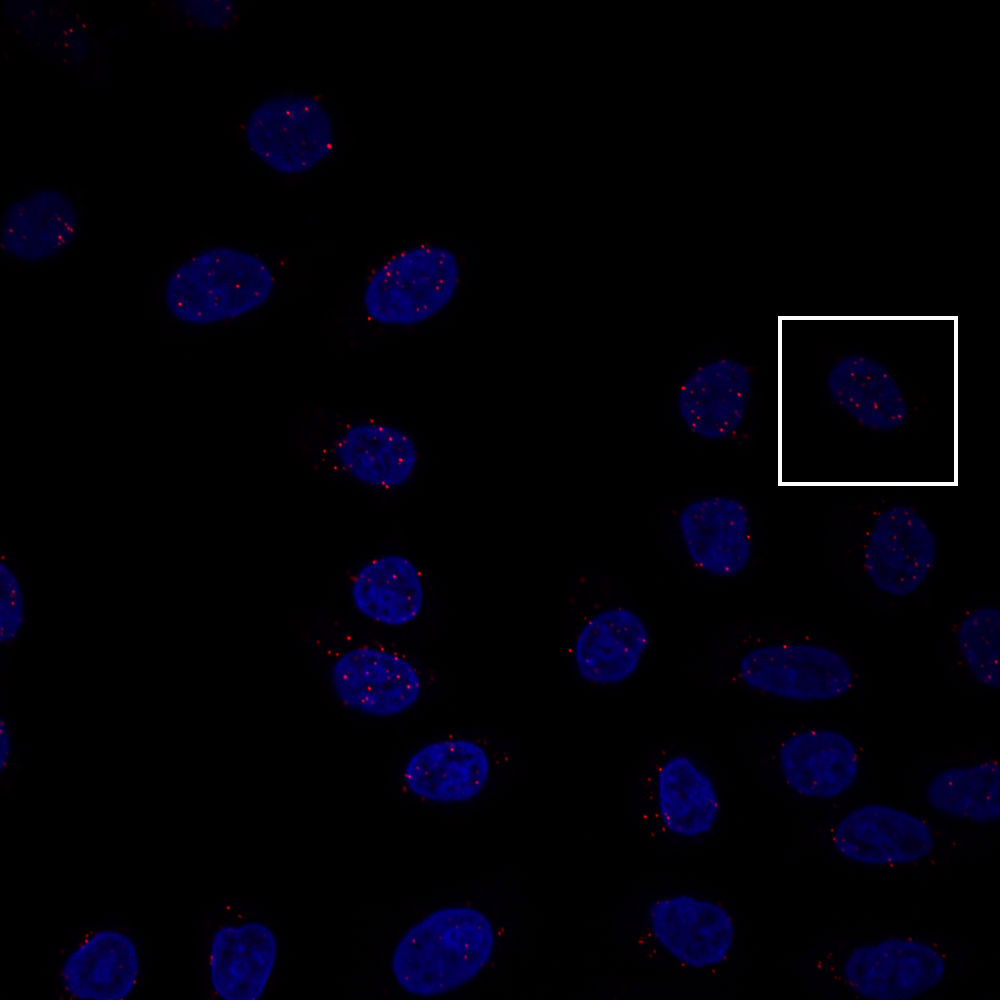

Supplement: Supplementary file 7 — Source data Fig. 5 [file 44318_2025_562_MOESM7_ESM.zip › Figure 5/5C/MCM5 GINS3 PLA/PLA immunostaining DMSO.tif]

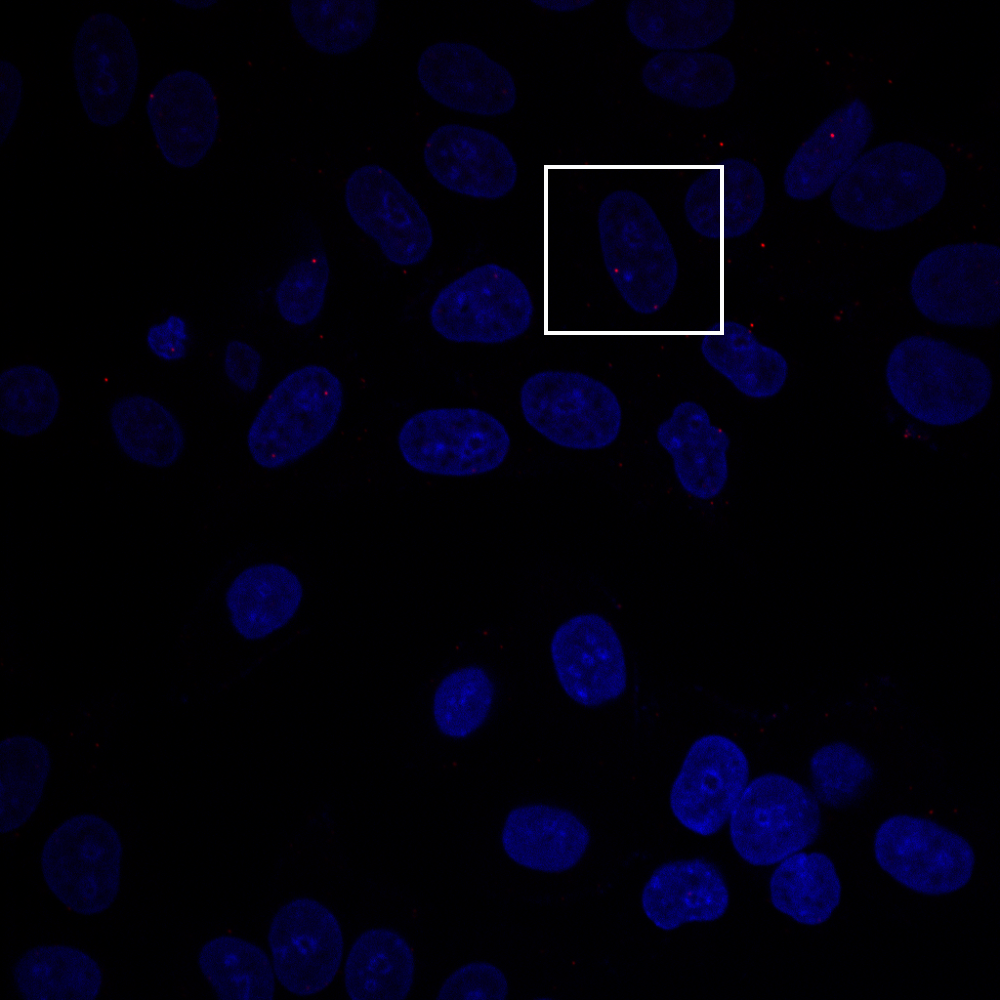

Supplement: Supplementary file 7 — Source data Fig. 5 [file 44318_2025_562_MOESM7_ESM.zip › Figure 5/5C/MCM5 GINS3 PLA/PLA immunostaining DKM 2-93.tif]

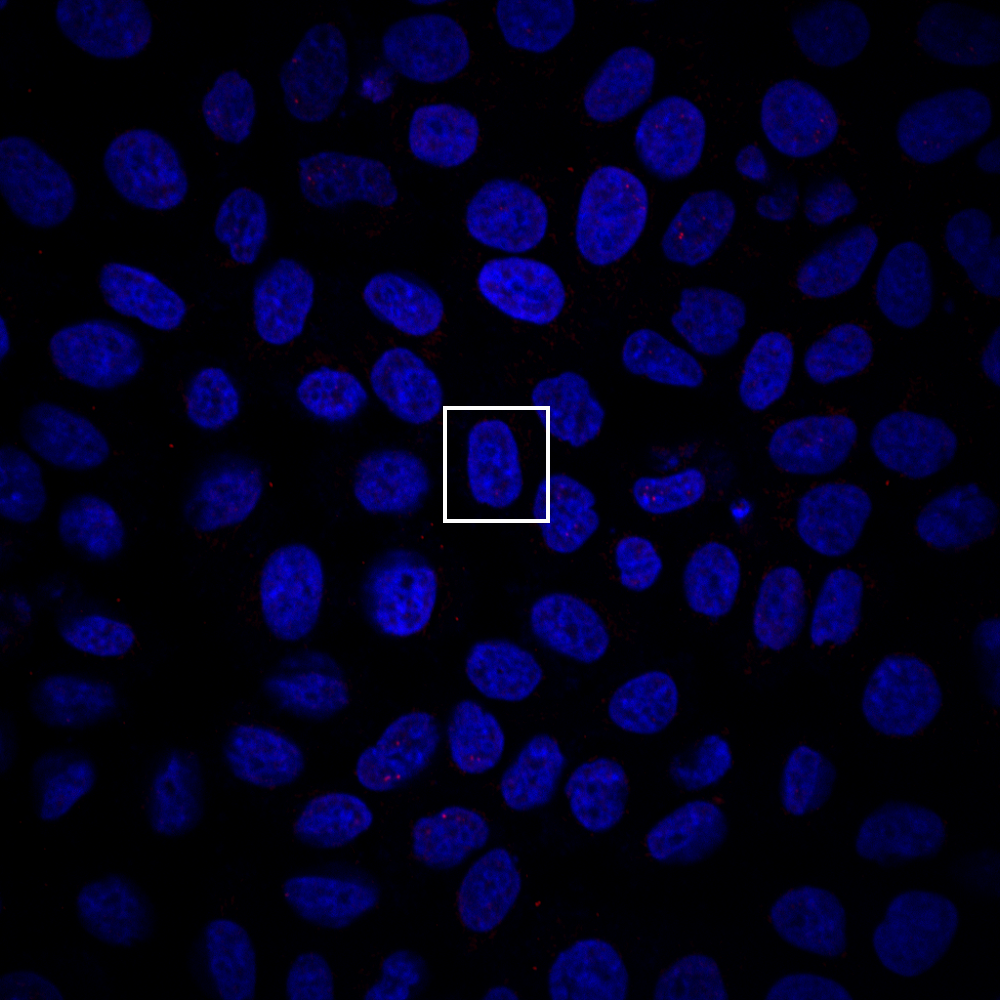

Supplement: Supplementary file 7 — Source data Fig. 5 [file 44318_2025_562_MOESM7_ESM.zip › Figure 5/5E/MCM5 CDC45 PLA/PLA immunostaining siUFL1.tif]

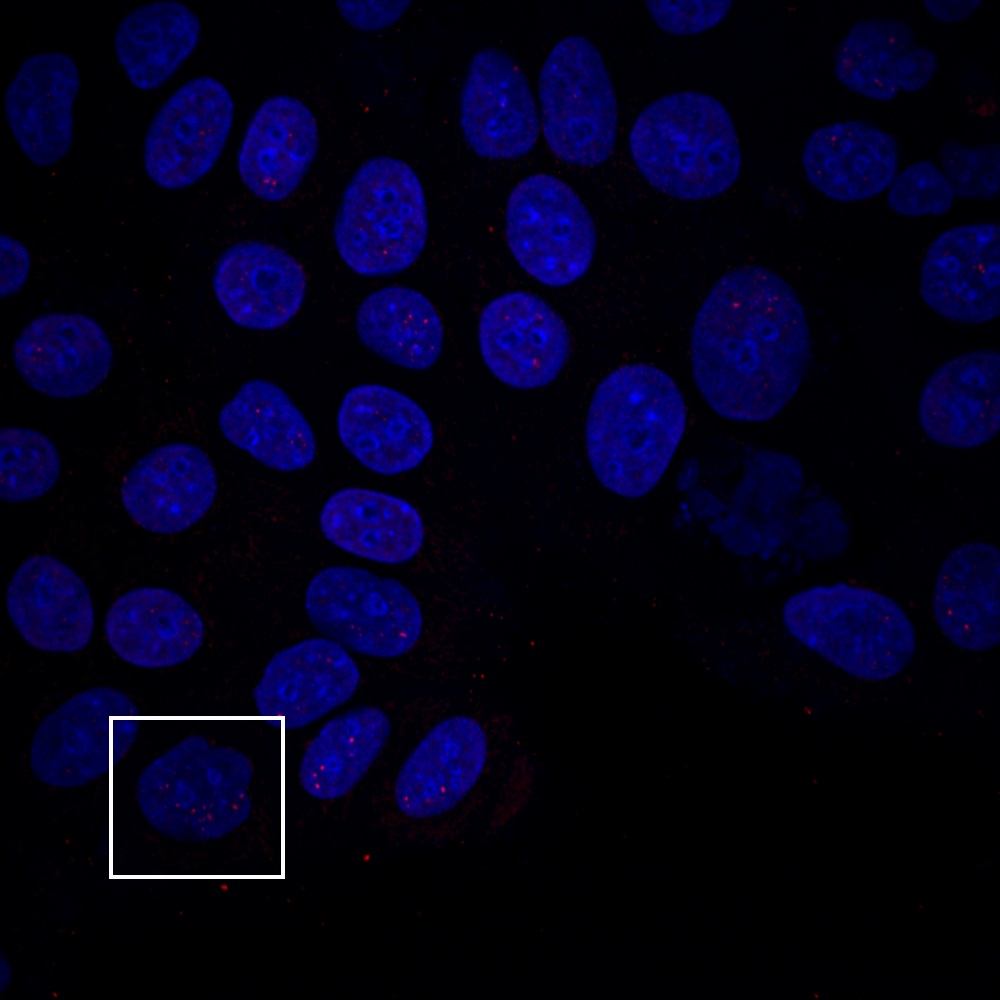

Supplement: Supplementary file 7 — Source data Fig. 5 [file 44318_2025_562_MOESM7_ESM.zip › Figure 5/5E/MCM5 CDC45 PLA/PLA immunostaining siNC.tif]

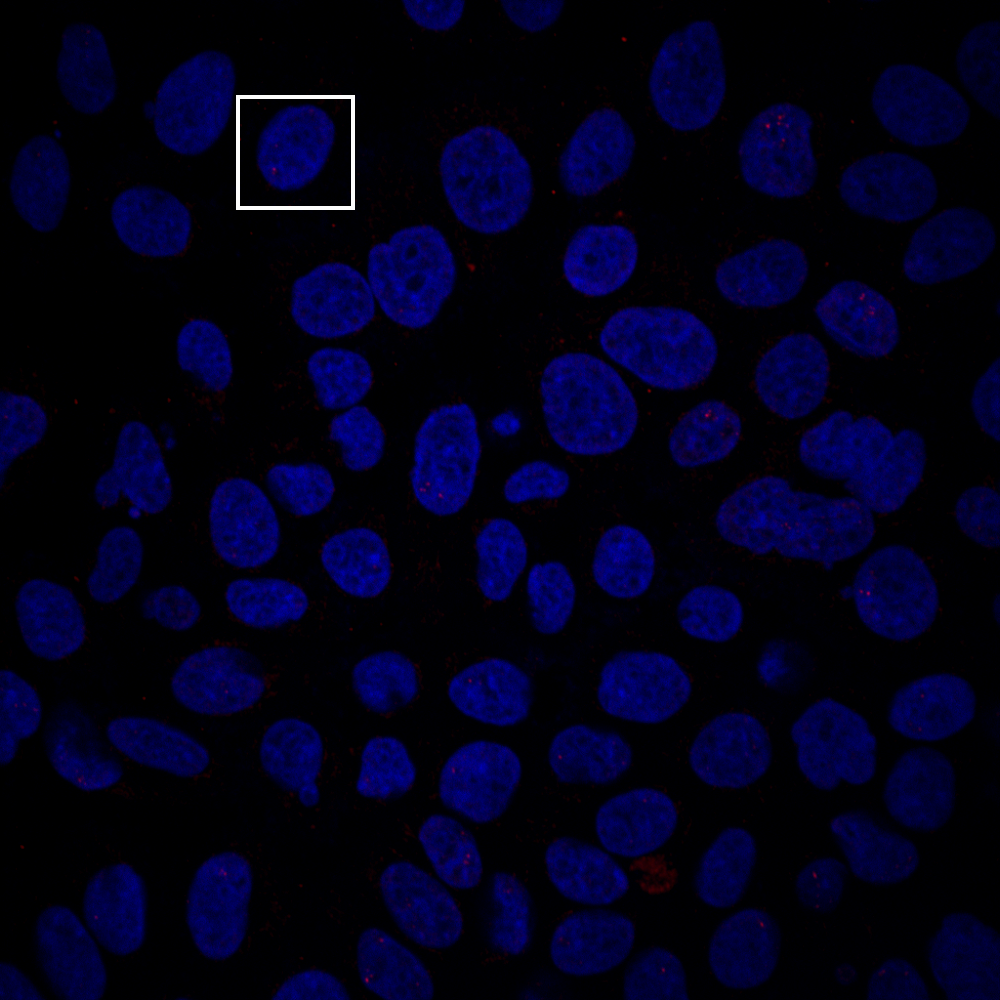

Supplement: Supplementary file 7 — Source data Fig. 5 [file 44318_2025_562_MOESM7_ESM.zip › Figure 5/5E/MCM5 GINS3 PLA/PLA immunostaining siUFL1.tif]

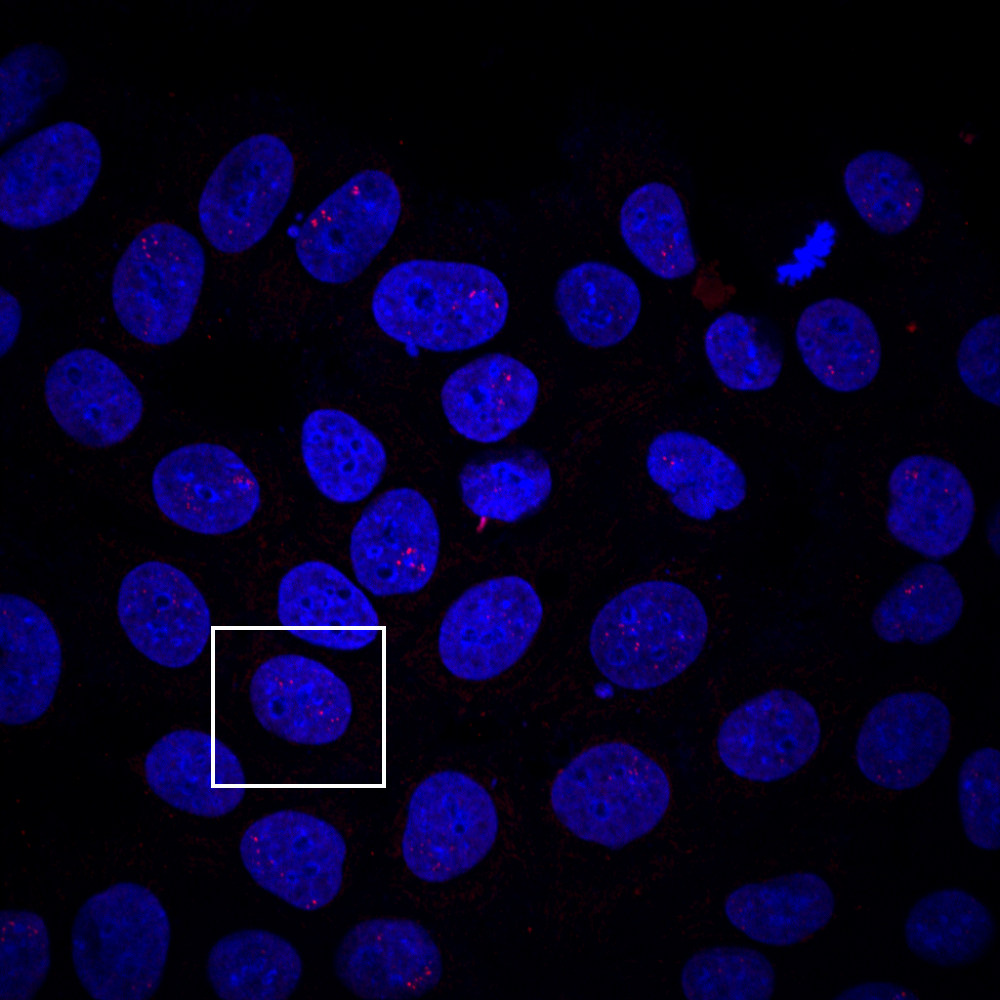

Supplement: Supplementary file 7 — Source data Fig. 5 [file 44318_2025_562_MOESM7_ESM.zip › Figure 5/5E/MCM5 GINS3 PLA/PLA immunostaining siNC.tif]

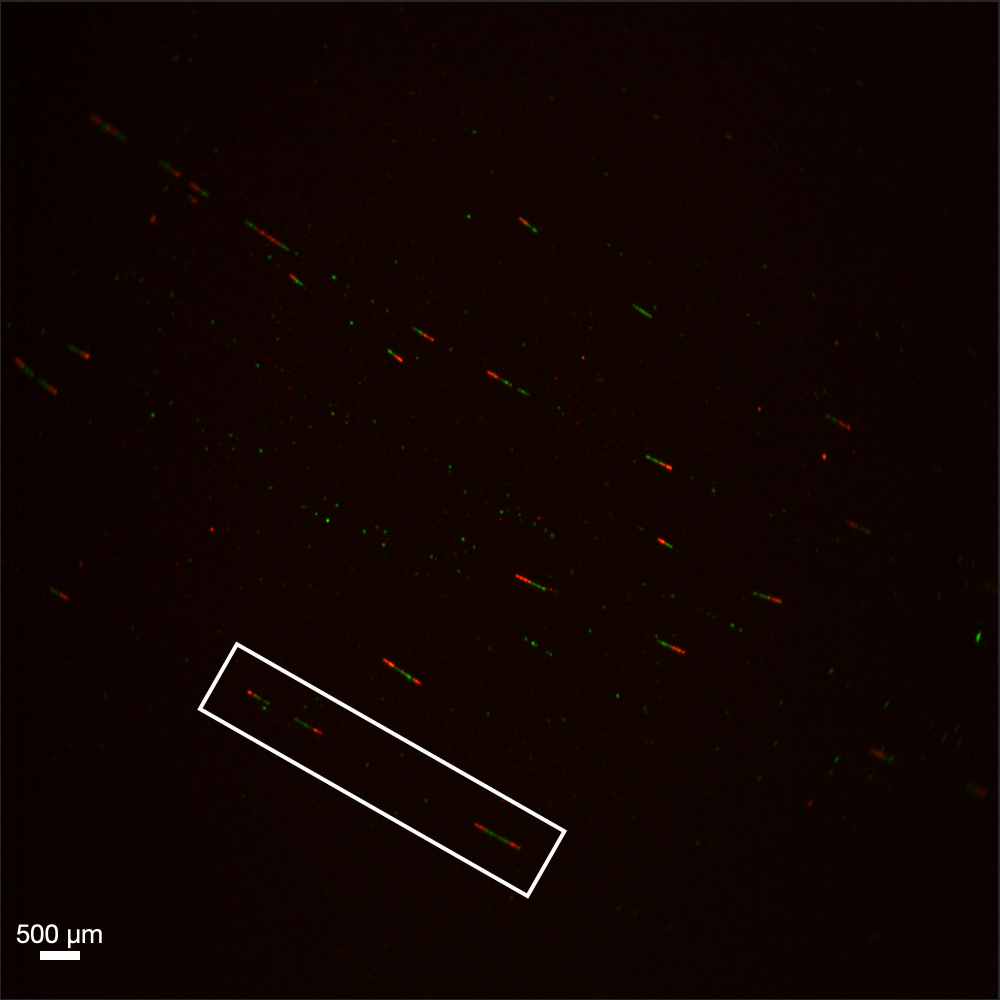

Supplement: Supplementary file 8 — Source data Fig. 6 [file 44318_2025_562_MOESM8_ESM.zip › Figure 6/6A/Fiber siMCM5.tif]

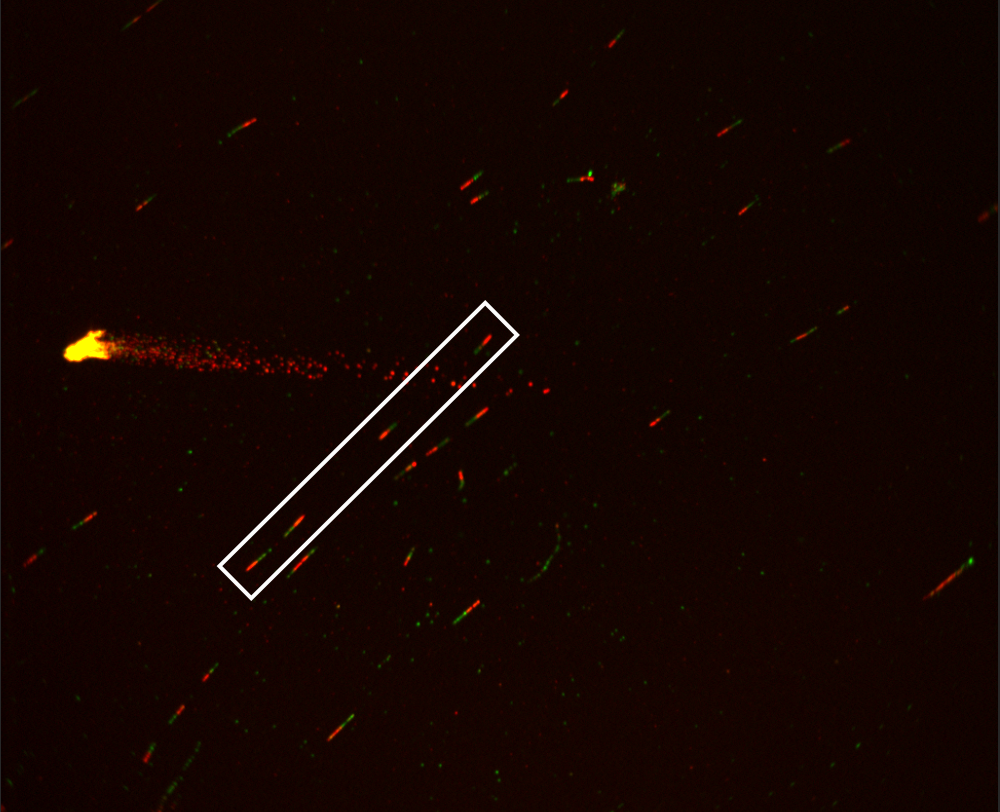

Supplement: Supplementary file 8 — Source data Fig. 6 [file 44318_2025_562_MOESM8_ESM.zip › Figure 6/6A/Fiber siMCM5+KR+DKM 2-93.tif]

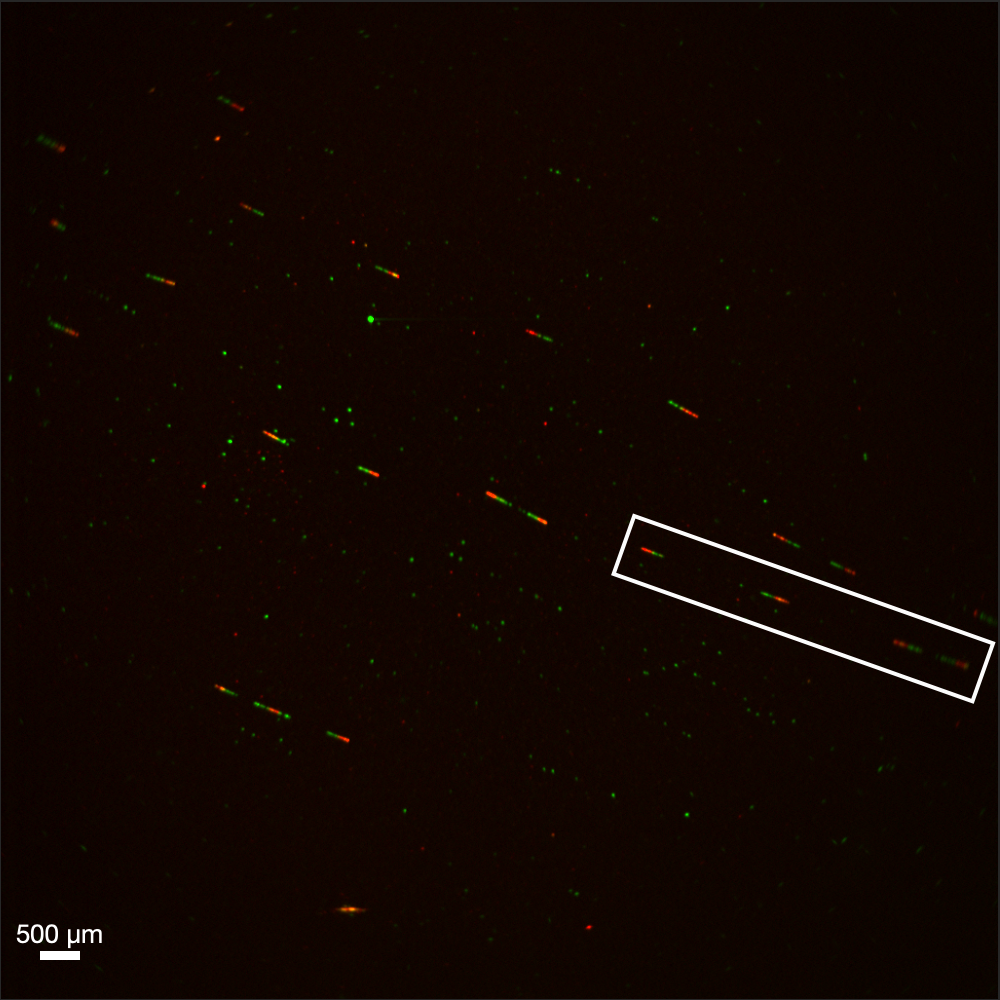

Supplement: Supplementary file 8 — Source data Fig. 6 [file 44318_2025_562_MOESM8_ESM.zip › Figure 6/6A/Fiber siMCM5+KR.tif]

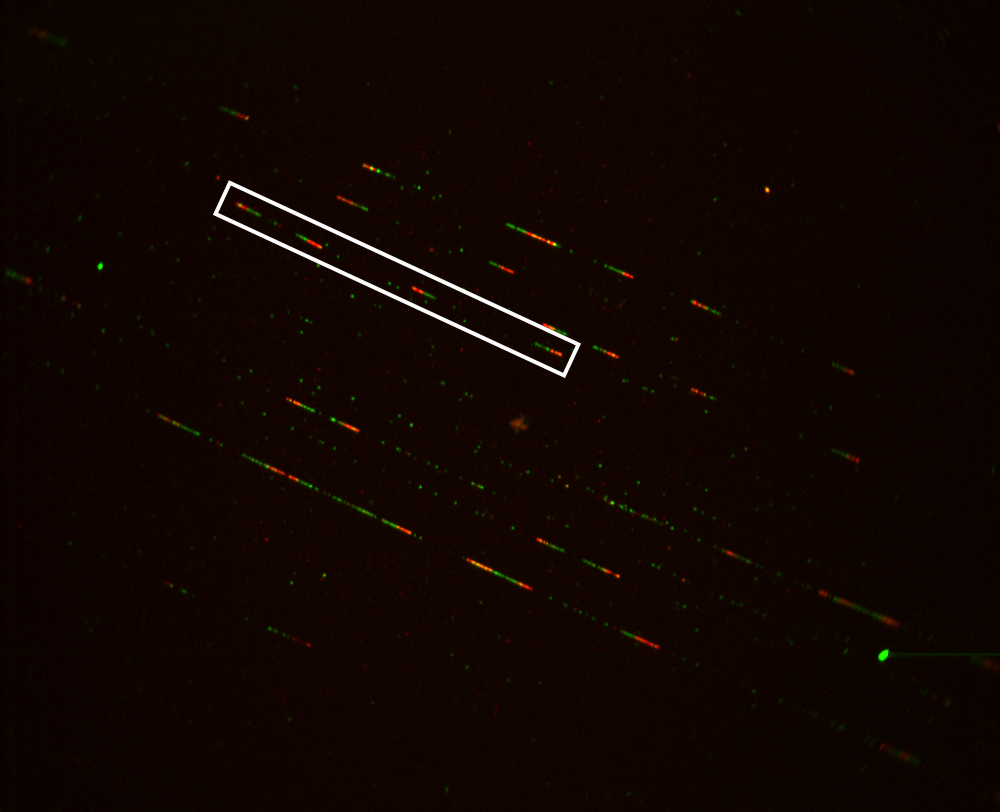

Supplement: Supplementary file 8 — Source data Fig. 6 [file 44318_2025_562_MOESM8_ESM.zip › Figure 6/6A/Fiber siMCM5+WT+DKM 2-93.tif]

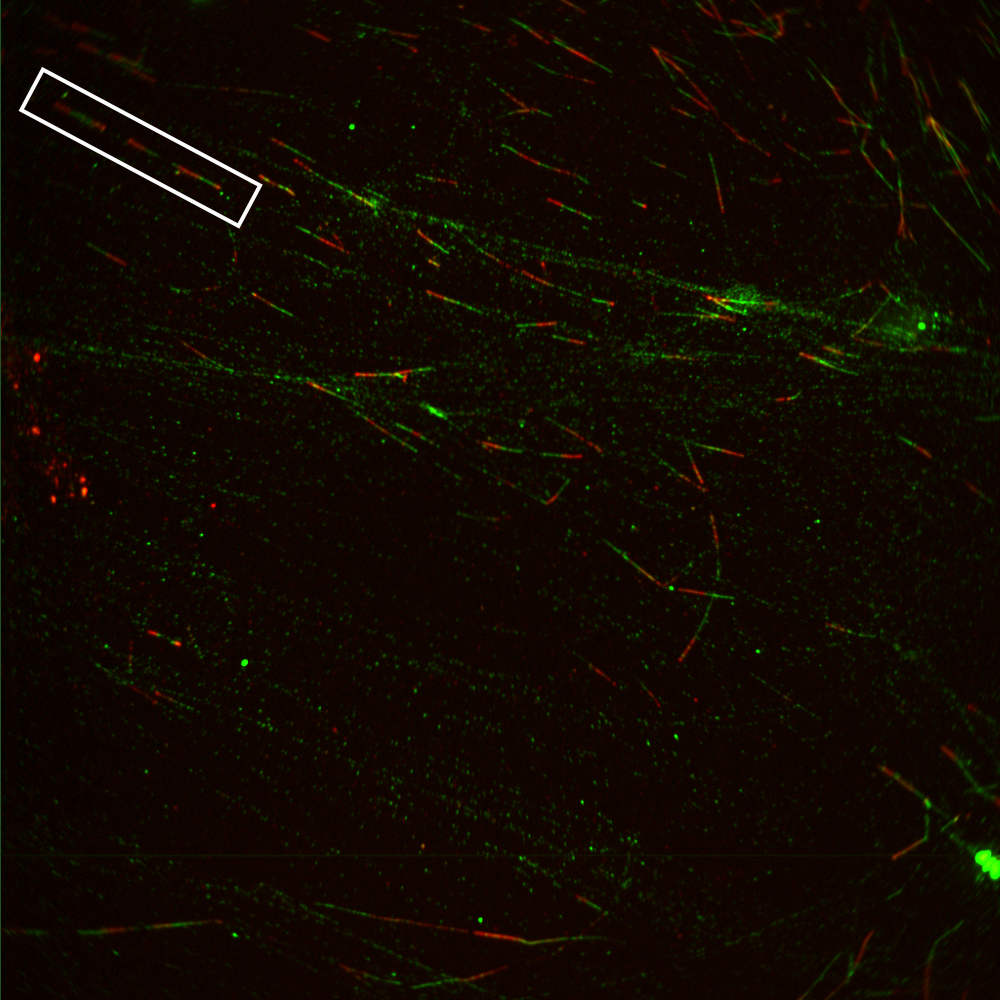

Supplement: Supplementary file 8 — Source data Fig. 6 [file 44318_2025_562_MOESM8_ESM.zip › Figure 6/6A/Fiber siNC.tif]

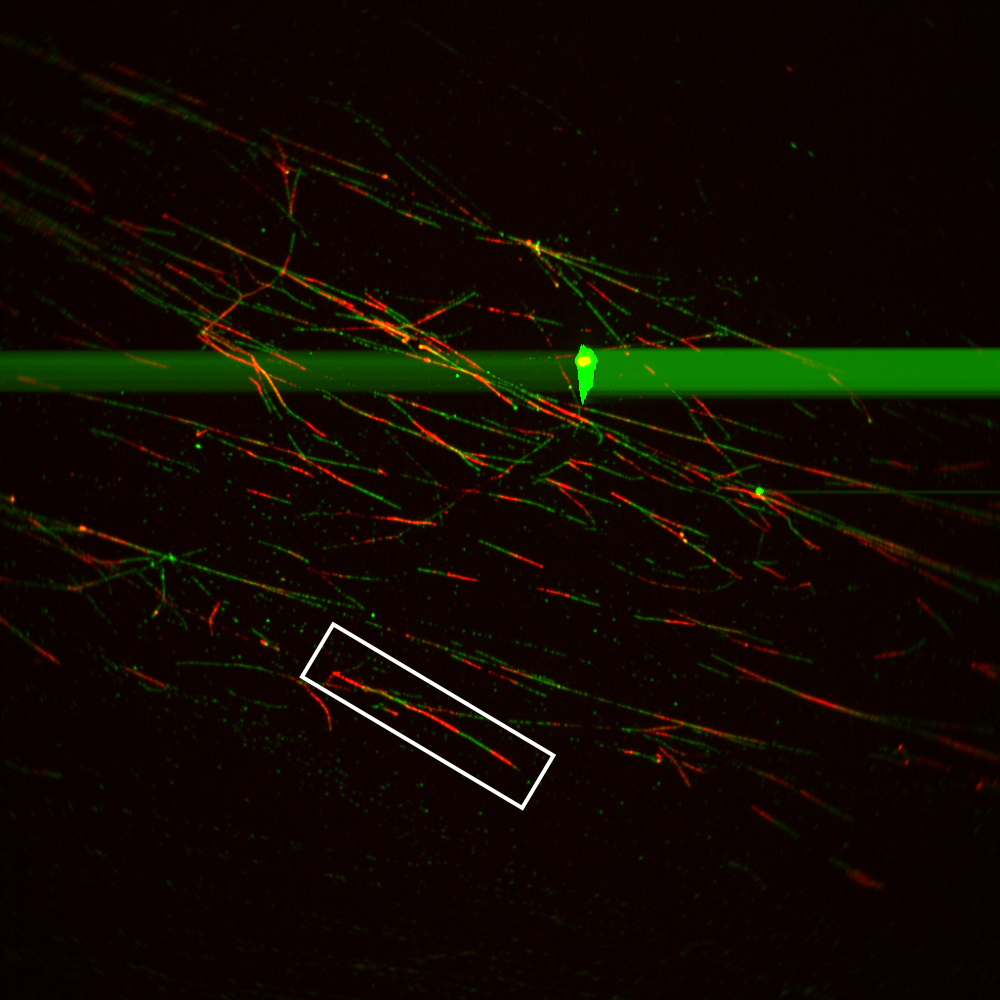

Supplement: Supplementary file 8 — Source data Fig. 6 [file 44318_2025_562_MOESM8_ESM.zip › Figure 6/6A/Fiber siMCM5+WT.tif]

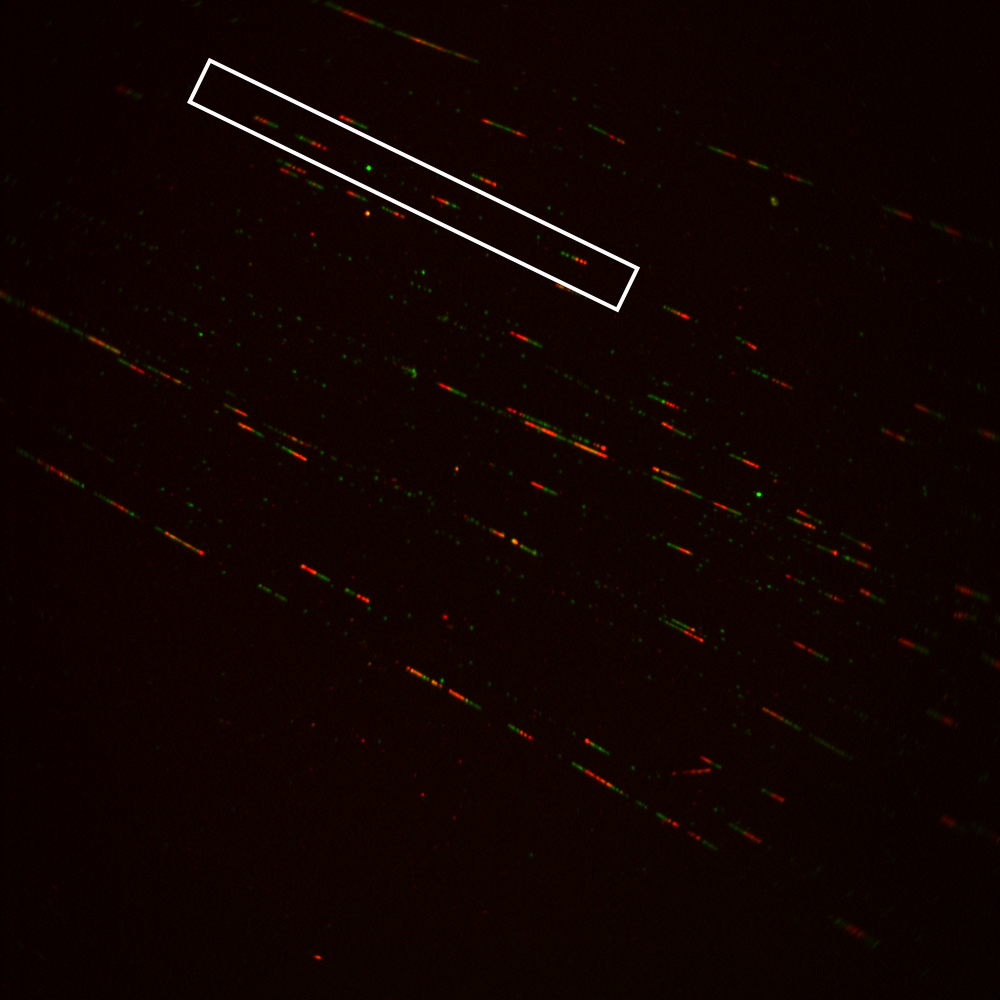

Supplement: Supplementary file 8 — Source data Fig. 6 [file 44318_2025_562_MOESM8_ESM.zip › Figure 6/6A/Fiber siMCM5+DKM 2-93.tif]

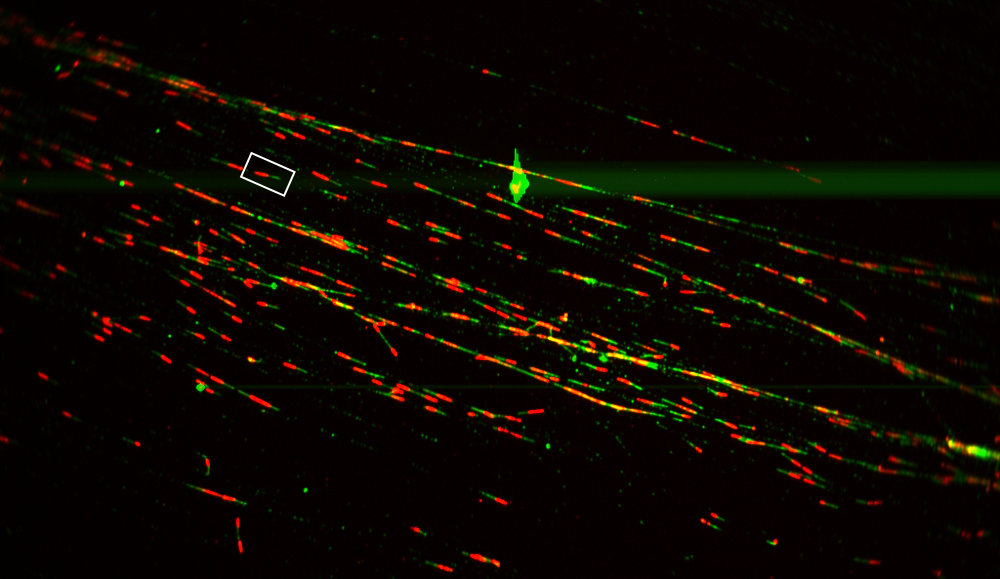

Supplement: Supplementary file 8 — Source data Fig. 6 [file 44318_2025_562_MOESM8_ESM.zip › Figure 6/6C/Fiber siMCM5+WT+DKM.tif]

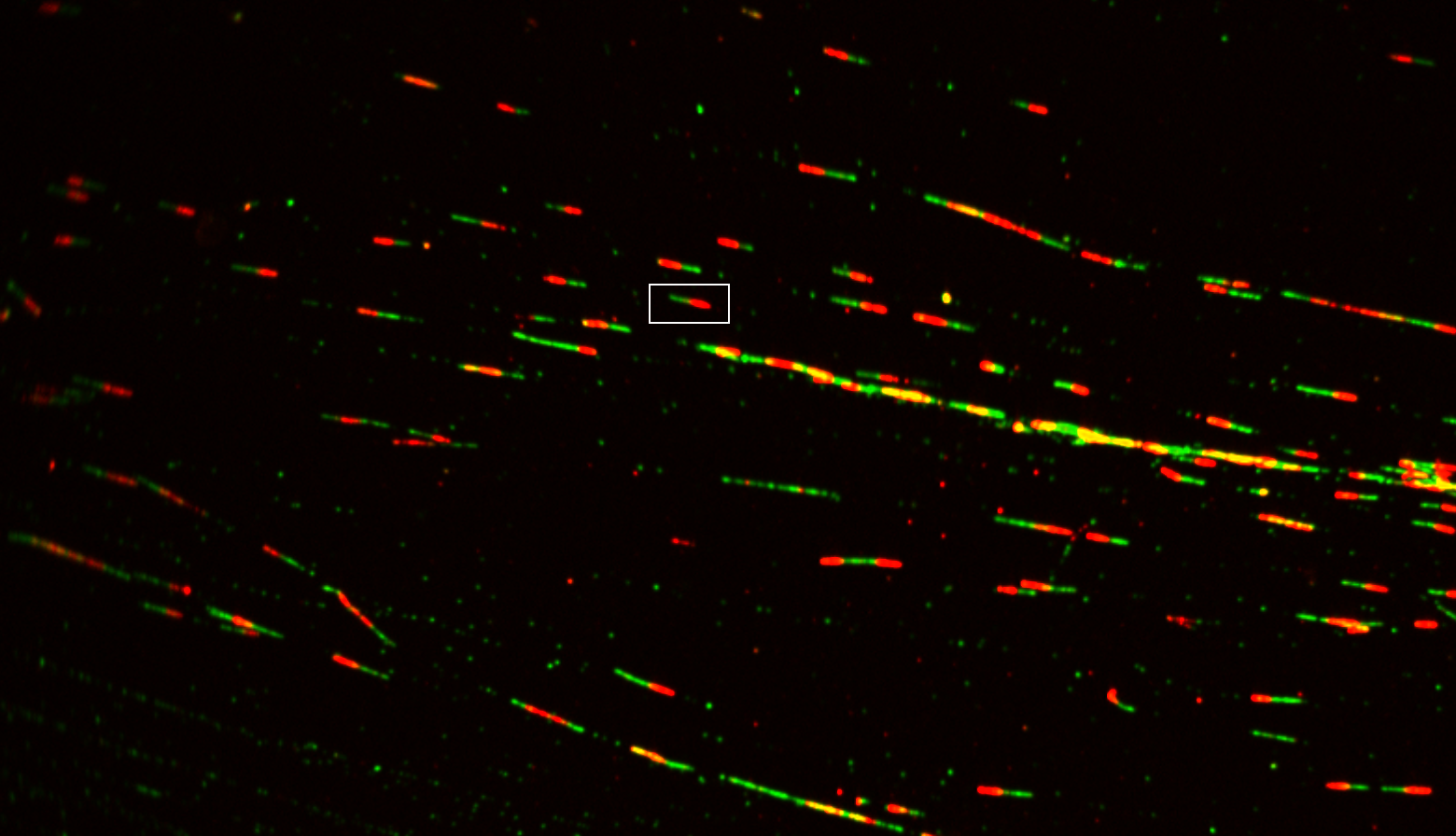

Supplement: Supplementary file 8 — Source data Fig. 6 [file 44318_2025_562_MOESM8_ESM.zip › Figure 6/6C/Fiber siMCM5.tif]

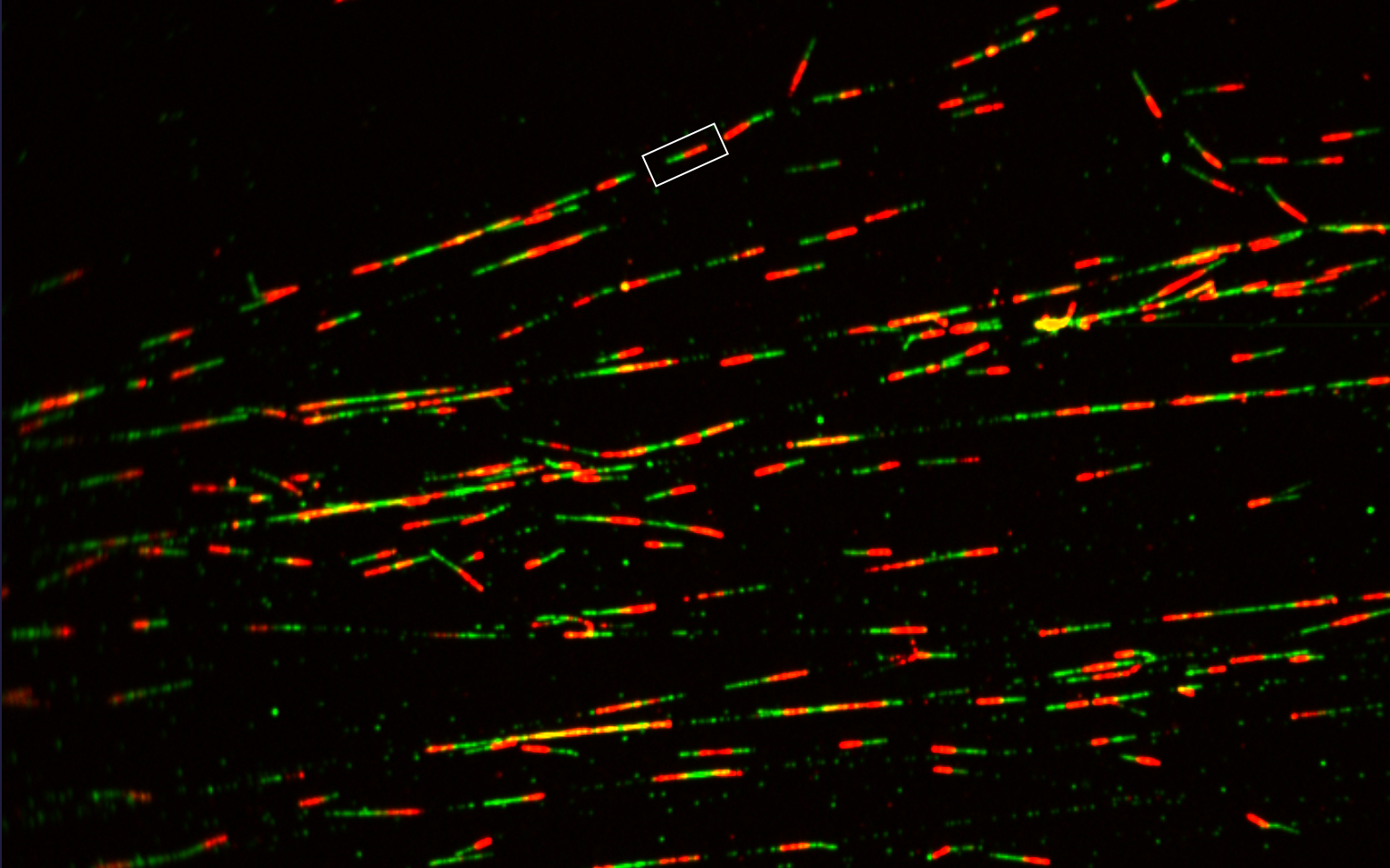

Supplement: Supplementary file 8 — Source data Fig. 6 [file 44318_2025_562_MOESM8_ESM.zip › Figure 6/6C/Fiber siMCM5+KR.tif]

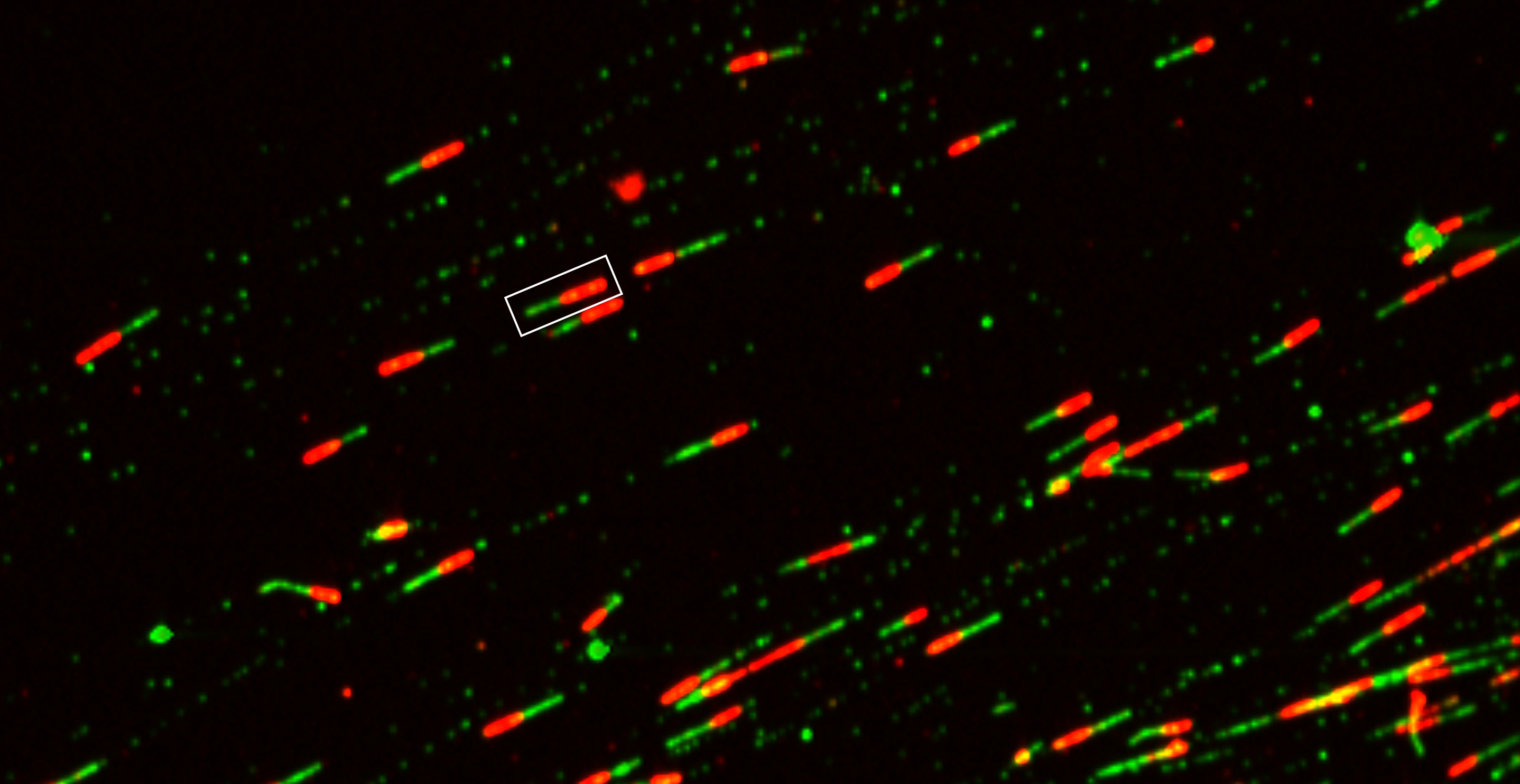

Supplement: Supplementary file 8 — Source data Fig. 6 [file 44318_2025_562_MOESM8_ESM.zip › Figure 6/6C/Fiber siMCM5+KR+DKM.tif]

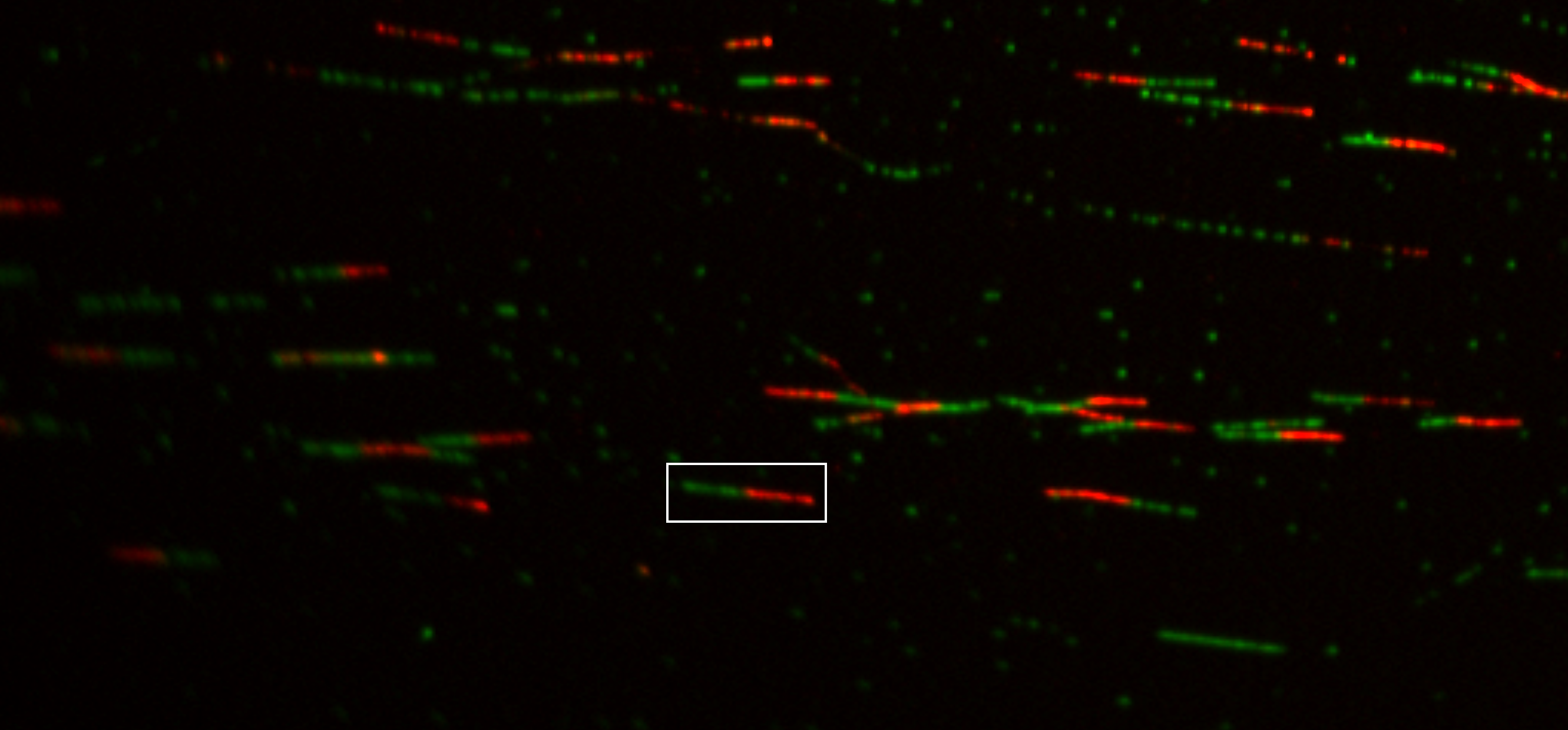

Supplement: Supplementary file 8 — Source data Fig. 6 [file 44318_2025_562_MOESM8_ESM.zip › Figure 6/6C/Fiber siNC.tif]

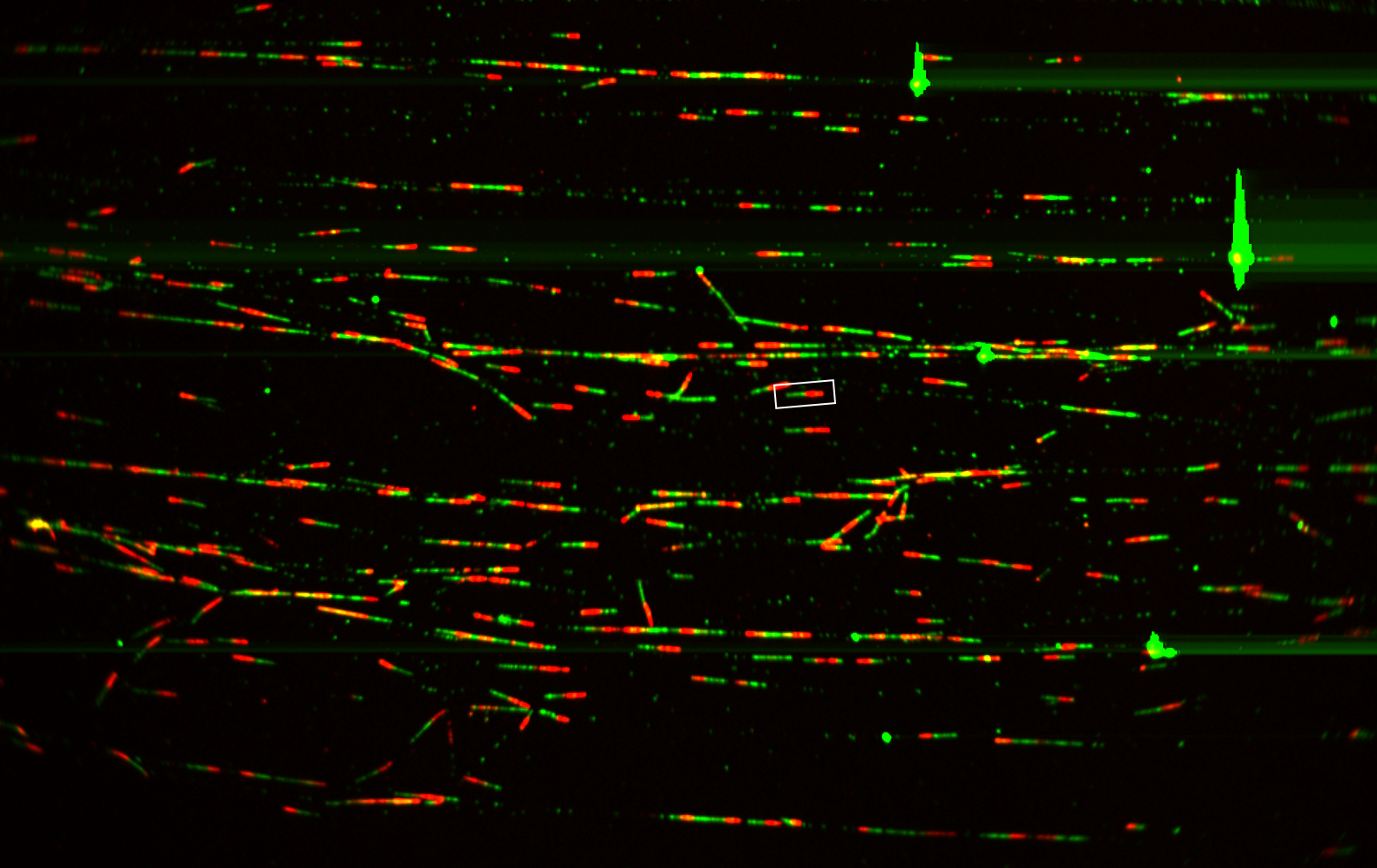

Supplement: Supplementary file 8 — Source data Fig. 6 [file 44318_2025_562_MOESM8_ESM.zip › Figure 6/6C/Fiber siMCM5+DKM.tif]

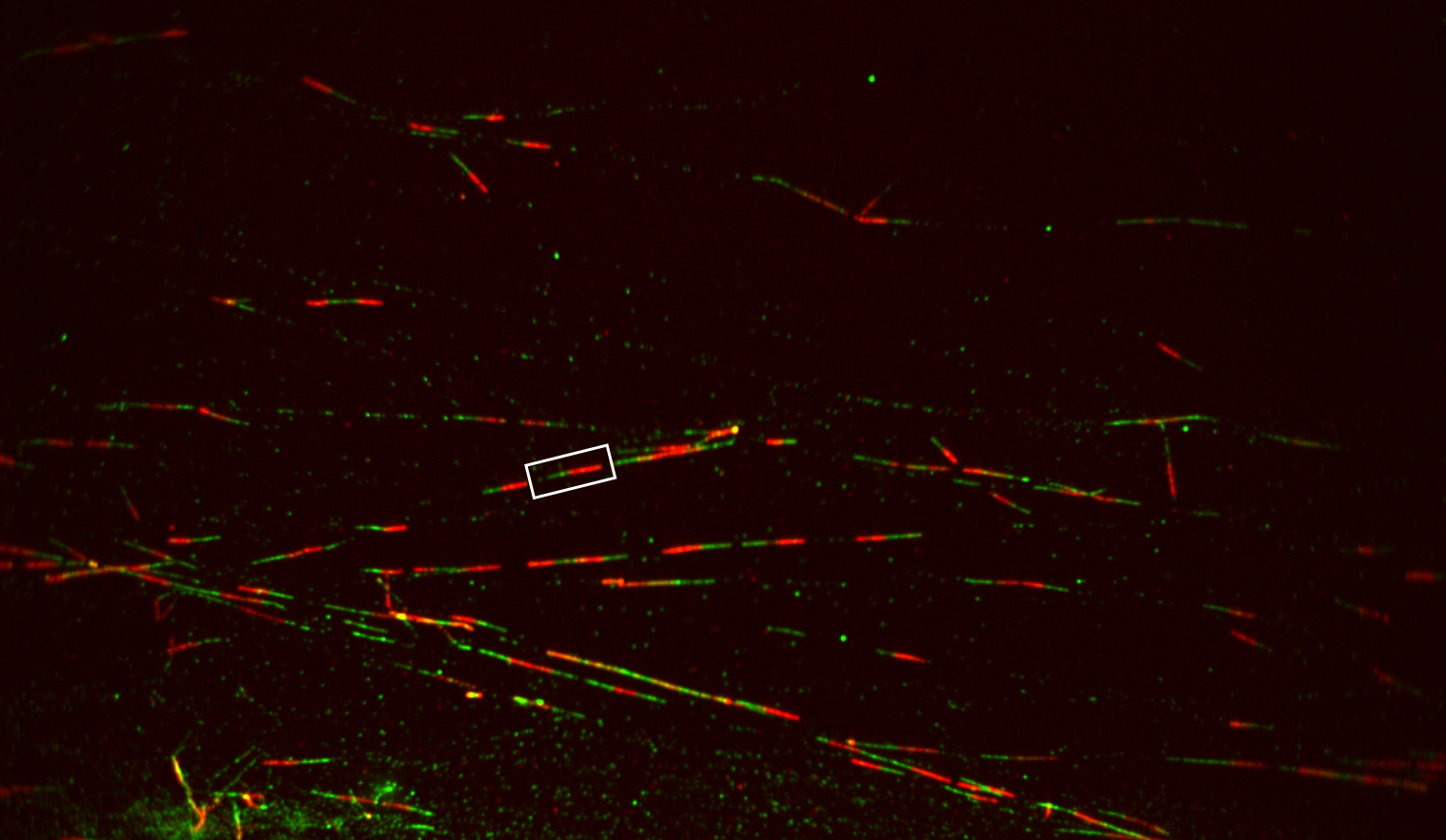

Supplement: Supplementary file 8 — Source data Fig. 6 [file 44318_2025_562_MOESM8_ESM.zip › Figure 6/6C/Fiber siMCM5+WT.tif]
