# Supplementary material for: MCM5 UFMylation regulates replication origin firing and fork progression
Source: EMBO J. 2025 Sep 12;44(21):6019–50. doi: 10.1038/s44318-025-00562-6 (PMC12583452; doi:10.1038/s44318-025-00562-6)
Supplement: Supplementary file 10 — Expanded View Figures [file 44318_2025_562_MOESM10_ESM.pdf]

Expanded View Figures

Figure EV1. UFMylation is required for DNA replication.

(**A**) Western blot analysis of protein expression in HeLa cells transfected with the indicated siRNAs and over-expression constructs. (**B**) Growth curves of the HeLa cells in (**A**). The mean number of cells (biological replicates, $n = 3$) ± SD is shown. *P* values were calculated by Two-way ANOVA (***$P < 0.001$; ****$P < 0.0001$). *P* values: siNC vs siUFL1, day 2/3/4, 5.33e-004/1.86e-009/9.67e-014. (**C**) Western blot analysis of UFL1 expression in *WT* or *UFL1* knockout (UFL1 KO) HeLa cells. (**D**) Growth curves of the indicated HeLa cells as in (**C**). The mean number of cells (biological replicates, $n = 3$) ± SD is shown. *P* values were calculated by Two-way ANOVA (****$P < 0.001$). *P* values: WT vs UFL1 KO, day 2/3/4, 8.97e-007/4.8e-012/2.71e-016. (**E**) Growth curves of the indicated HeLa cells. The mean number of cells (biological replicates, $n = 3$) ± SD is shown. *P* values were calculated by Two-way ANOVA (****$P < 0.001$; ns $P > 0.05$, no significance). *P* values: WT vs WT + DKM2-93, day 4, 1.66e-013; UFL1 KO vs UFL1 KO + DKM 2-93, day 4, 0.713. (**F**, **G**) Quantification of the EdU MFI in U2OS (**F**) and A549 (**G**) cells pre-treated with DMSO or DKM 2-93 (100 μM). Data are presented as mean ± SD of three biological replicates ($n = 3$). *P* value was calculated by unpaired *t* test with Welch's correction (***$P < 0.001$; ****$P < 0.0001$). *P* value: DMSO vs DKM 2-93 in (**E**)/(**F**), 2.77e-007/1.35e-004. (**H**) Quantification of the ratio of red-green-red (RGR) tracks to the total number of red-green (RG) fork tracks. Data are presented as mean ± SD. *P* value was calculated by unpaired *t* test with Welch's correction (****$P < 0.0001$). *P* value, 4.20352928e-005. (**I**) Quantification of the IdU track length in U2OS (Left) and A549 (Right) cells transfected with the indicated siRNAs and plasmids and sequentially labeled with CldU and IdU for 30 min. Data are presented as in Fig. 1E. Data are presented as mean ± SD. *n* DNA fiber number. *P* values were calculated by Ordinary one-way ANOVA (****$P < 0.0001$). *P* values: siNC vs siUFL1 in (**H**)/(**I**), 1.78e-025/1.89e-019; siUFL1 vs siUFL1+HA-UFL1 in (**H**)/(**I**), 4.52e-018/3.11e-013. (**J**) Western blot analysis of protein expression in HeLa cells transfected with the indicated siRNAs. (**K**, **L**) Quantification of the IdU track length in U2OS (**K**) and A549 (**L**) cells pre-treated with DKM 2-93 (100 μM) for 1 h before CldU and IdU labeling in the presence of DKM 2-93 for 30 min. Data are presented as mean ± SD. *n* DNA fiber number. *P* values were calculated by unpaired *t* test with Welch's correction (****$P < 0.0001$). *P* values: DMSO vs DKM 2-93 in (**K**)/(**L**), 9.04e-012/9.31e-016. (**M**) Western blot analysis of protein expression in HeLa cells treated with DMSO, DKM 2-93 (100 μM), hydroxyurea (2 mM), or bleomycin (10 μg/ml) for 1 h. Asterisk represents non-specific band. (**N**–**P**) Western blot analysis of protein expression in indicated HeLa cells used in Fig. 1G.

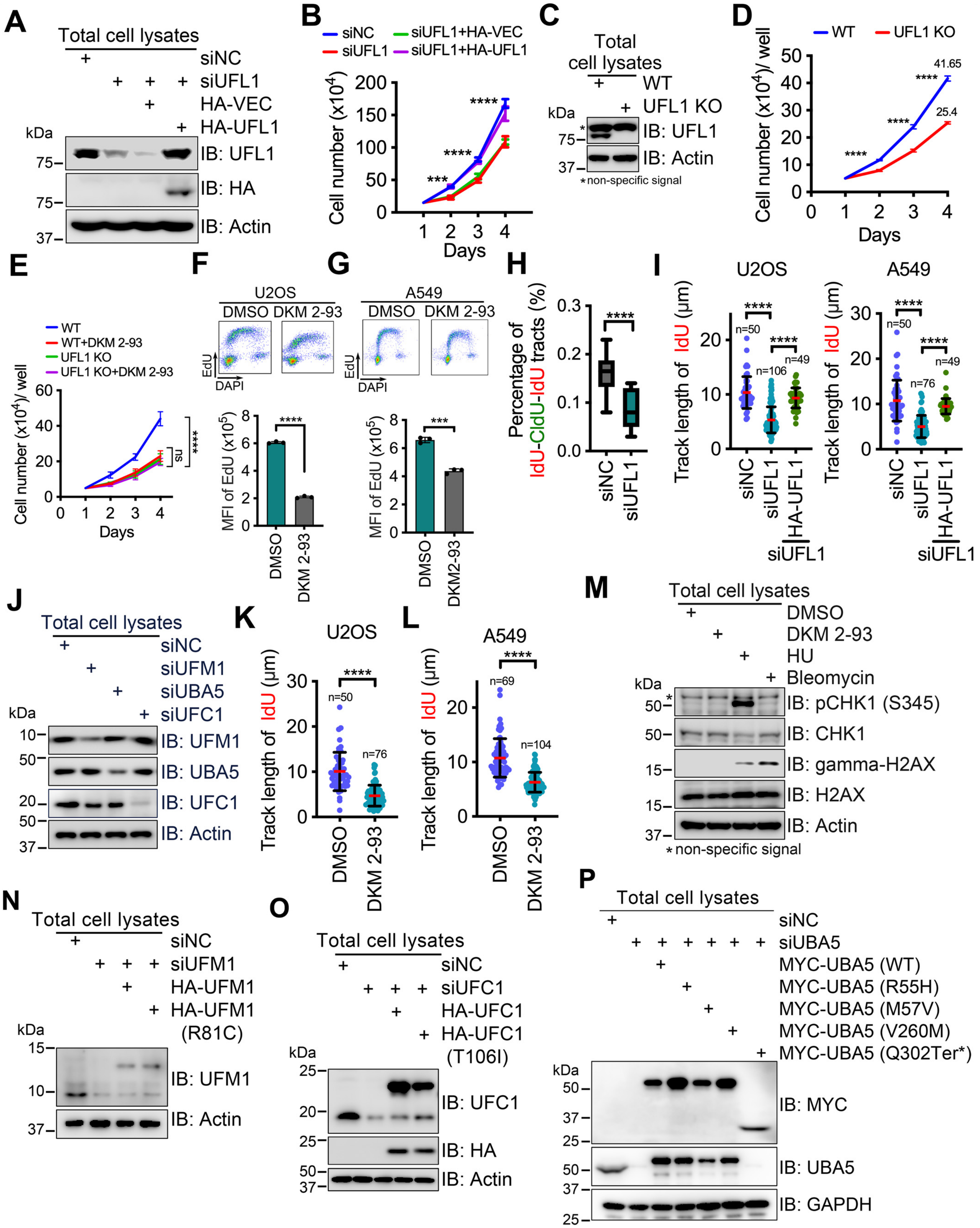
A
Total cell lysates
siNC
siUFL1
HA-VEC
HA-UFL1
IB: UFL1
IB: HA
IB: Actin
B
siNC siUFL1 siUFL1+HA-VEC siUFL1+HA-UFL1
Cell number (x10^4)
Days
C
Total cell lysates
WT
UFL1 KO
IB: UFL1
IB: Actin
*non-specific signal
D
WT UFL1 KO
Cell number (x10^4)/ well
41.65
25.4
Days
E
WT
WT+DKM 2-93
UFL1 KO
UFL1 KO+DKM 2-93
Cell number (x10^4)/ well
Days
F
U2OS
DMSO DKM 2-93
EdU
DAPI
MFI of EdU (x10^5)
G
A549
DMSO DKM 2-93
MFI of EdU (x10^5)
H
Percentage of IdU-CldU-IdU tracts (%)
siNC siUFL1
I
U2OS A549
Track length of IdU (μm)
siNC siUFL1 HA-UFL1 siUFL1
J
Total cell lysates
siNC
siUFM1
siUBA5
siUFC1
IB: UFM1
IB: UBA5
IB: UFC1
IB: Actin
K
U2OS
Track length of IdU (μm)
DMSO DKM 2-93
L
A549
Track length of IdU (μm)
DMSO DKM 2-93
M
Total cell lysates
DMSO
DKM 2-93
HU
Bleomycin
IB: pCHK1 (S345)
IB: CHK1
IB: gamma-H2AX
IB: H2AX
IB: Actin
*non-specific signal
N
Total cell lysates
siNC
siUFM1
HA-UFM1
HA-UFM1 (R81C)
IB: UFM1
IB: Actin
O
Total cell lysates
siNC
siUFC1
HA-UFC1
HA-UFC1 (T106I)
IB: UFC1
IB: HA
IB: Actin
P
Total cell lysates
siNC
siUBA5
MYC-UBA5 (WT)
MYC-UBA5 (R55H)
MYC-UBA5 (M57V)
MYC-UBA5 (V260M)
MYC-UBA5 (Q302Ter*)
IB: MYC
IB: UBA5
IB: GAPDH

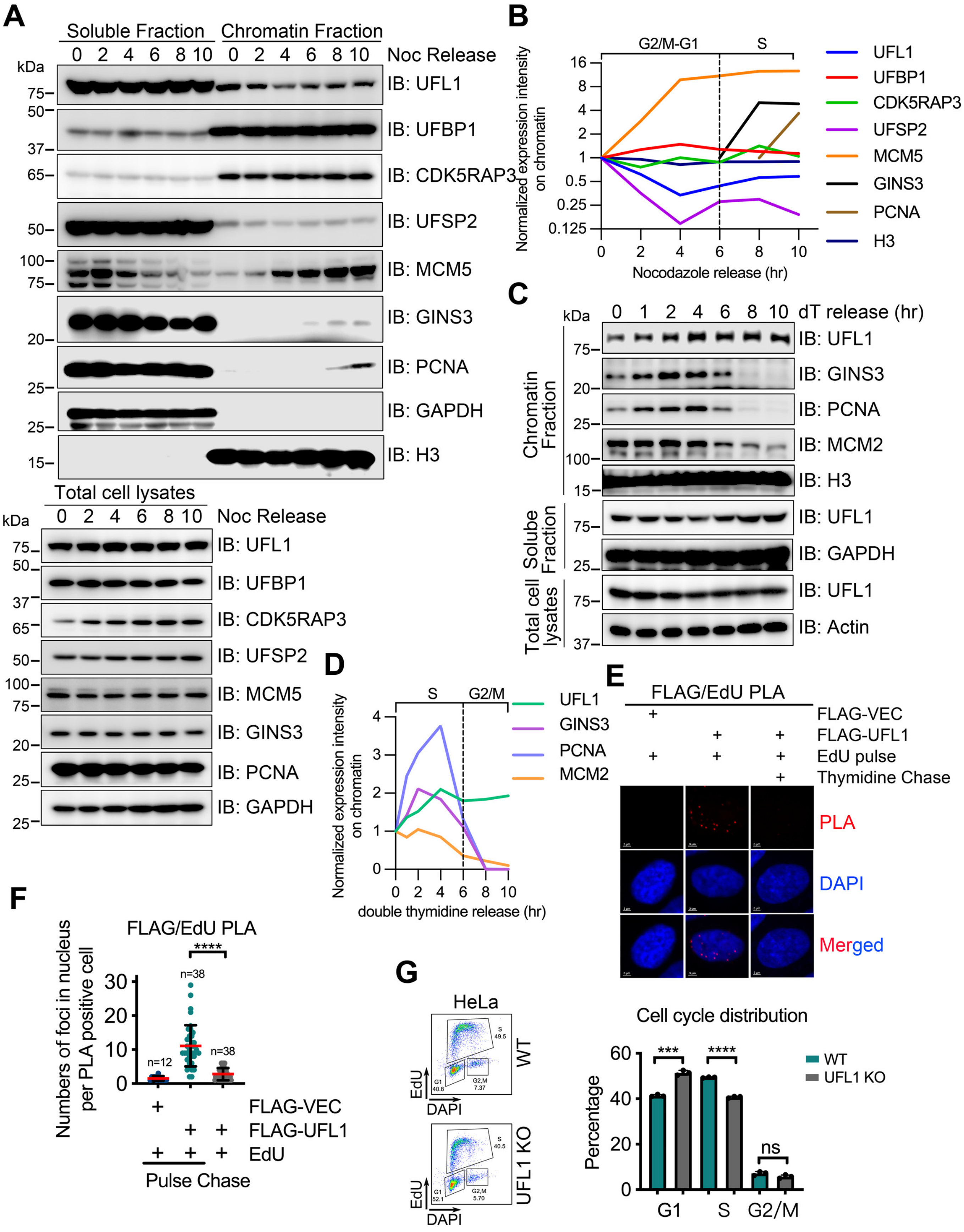
A
Soluble Fraction
Chromatin Fraction
0 2 4 6 8 10 0 2 4 6 8 10 Noc Release
kDa
IB: UFL1
IB: UFBP1
IB: CDK5RAP3
IB: UFSP2
IB: MCM5
IB: GINS3
IB: PCNA
IB: GAPDH
IB: H3
Total cell lysates
0 2 4 6 8 10 Noc Release
IB: UFL1
IB: UFBP1
IB: CDK5RAP3
IB: UFSP2
IB: MCM5
IB: GINS3
IB: PCNA
IB: GAPDH
B
G2/M-G1
S
Normalized expression intensity on chromatin
Nocodazole release (hr)
UFL1
UFBP1
CDK5RAP3
UFSP2
MCM5
GINS3
PCNA
H3
C
kDa
0 1 2 4 6 8 10 dT release (hr)
Chromatin Fraction
Solube Fraction
Total cell lysates
IB: UFL1
IB: GINS3
IB: PCNA
IB: MCM2
IB: H3
IB: UFL1
IB: GAPDH
IB: UFL1
IB: Actin
D
S
G2/M
Normalized expression intensity on chromatin
double thymidine release (hr)
UFL1
GINS3
PCNA
MCM2
E
FLAG/EdU PLA
FLAG-VEC
FLAG-UFL1
EdU pulse
Thymidine Chase
PLA
DAPI
Merged
F
FLAG/EdU PLA
Numbers of foci in nucleus per PLA positive cell
n=12
n=38
n=38
****
FLAG-VEC
FLAG-UFL1
EdU
Pulse Chase
G
HeLa
WT
UFL1 KO
EdU
DAPI
S 49.5
G1 40.8
G2,M 7.37
S 40.5
G1 52.1
G2,M 5.70
Cell cycle distribution
Percentage
***
****
ns
WT
UFL1 KO
G1
S
G2/M

Figure EV2. UFL1 is present at replication origins and affects their timing.

(**A–D**) The protein level of UFL1 and replication factors in the soluble fraction, chromatin fraction and total cell lysates of HeLa cells synchronized with nocodazole (**A**, **B**) and dT (**C**, **D**) block. At the time zero, cells were released to the cell cycle. Western blot analysis of proteins is presented in (**A**, **C**), and the quantification of analyzed proteins normalized to H3 is presented in (**B**, **D**). (**E**, **F**) Quantification of PLA foci in HeLa cells transiently transfected with FLAG-VEC or FLAG-UFL1. Cells were labeled with EdU for 30 min. After labeling, the chase-group cells were additionally labeled with thymidine for 30 min. Representative images of PLA foci (red) (**E**) and the average number of foci per nucleus (**F**) are shown. *n* cell number. *P* values was calculated by unpaired *t* test with Welch's correction (****$P < 0.0001$). *P* value: pulse vs chase in FLAG-UFL1 expressed group, 3.22e-010. Scale bar, 3 μm. (**G**) Cell cycle analysis of *WT* or UFL1 KO HeLa cells. The *X*-axis is the intensity of DAPI staining on a linear scale, and the *Y*-axis is EdU uptake on a log scale. *WT* or UFL1 KO HeLa cells were pulse-labeled with EdU for 30 min. Representative data and quantifications are shown in the left and right panels, respectively. Data are presented as mean ± SD of three biological replicates ($n = 3$). *P* value was calculated by Ordinary one-way ANOVA (***$P < 0.001$; ****$P < 0.0001$; ns $P > 0.05$, no significance). *P* value: WT vs UFL1 KO in G1 phase/S phase/G2/M phase, 1.27e-009/6.93e-009/0.0691.

 © The Author(s)

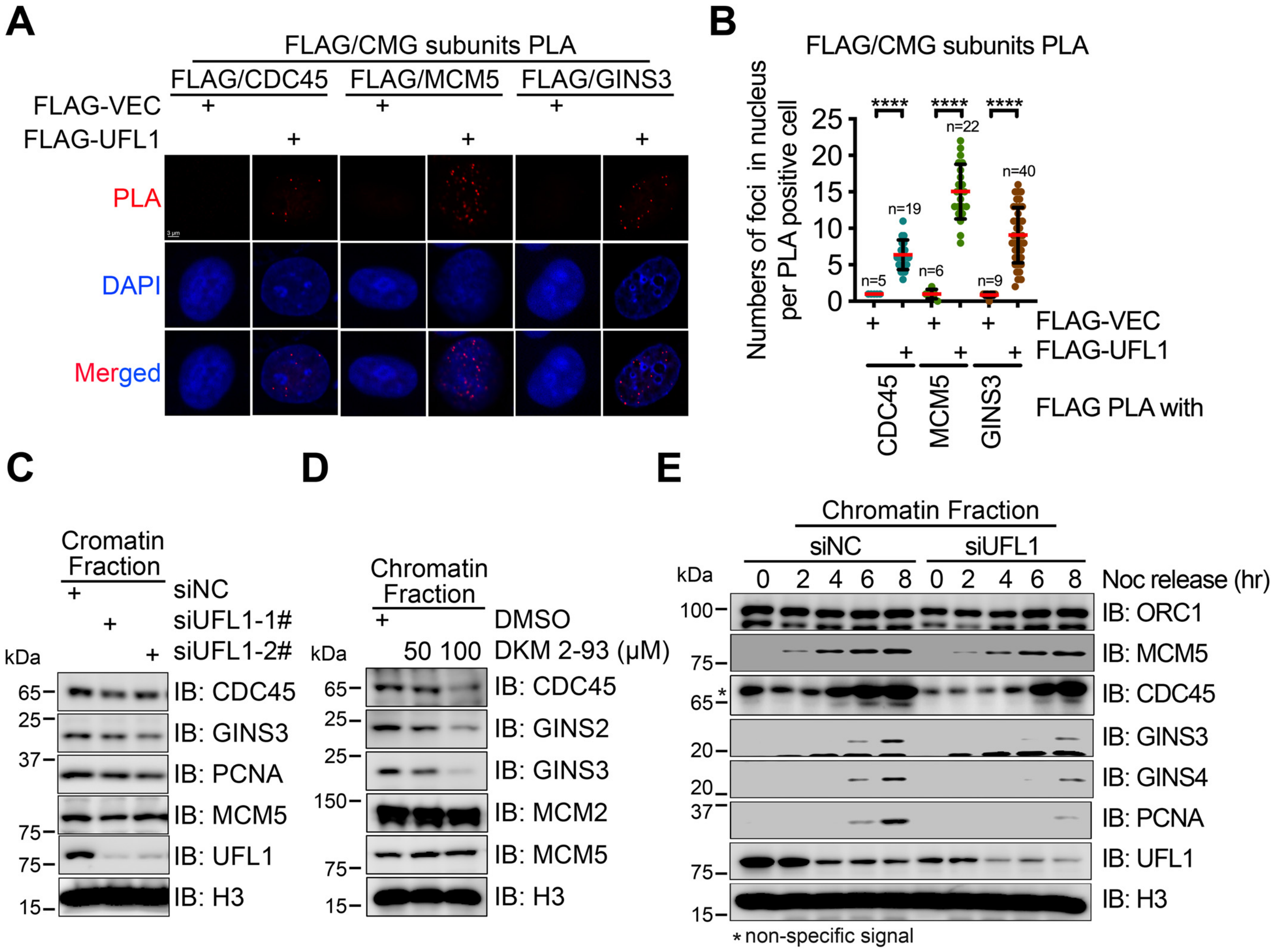

Figure EV3. UFL1 binds to CMG helicase components and affects their loading to replisome.

(**A**, **B**) PLA assays to analyze the proximal localization of UFL1 by CDC45, GINS3, and MCM5 in HeLa cells transfected with FLAG-VEC or FLAG-UFL1. Representative images of PLA foci (red) (**A**) and the average number of foci per nucleus per PLA positive cell (**B**) are shown. The scale bar is 3 μm. Data are presented as mean ± SD. *n* cell number. *P* values was calculated by unpaired *t* test with Welch's correction (*****P* < 0.0001). *P* value: FLAG-VEC vs FLAG-UFL1 in CDC45/MCM5/GINS3 group, 9.87e-010/6.07e-015/1.08e-016. Scale bar, 3 μm. (**C**) Western blot analysis of chromatin fractions of HeLa cells that were transfected with a non-targeting control (siNC) or two siRNA oligos targeting UFL1 (siUFL1-1#, siUFL1-2#). (**D**) Western blot analysis of chromatin fractions of HeLa cells that were treated with DMSO or DKM 2-93 (100 μM, 16 h). (**E**) Western blot analysis of chromatin fractions of HeLa cells that were transfected with siNC or siUFL1 at the indicated time points after release from nocodazole block.

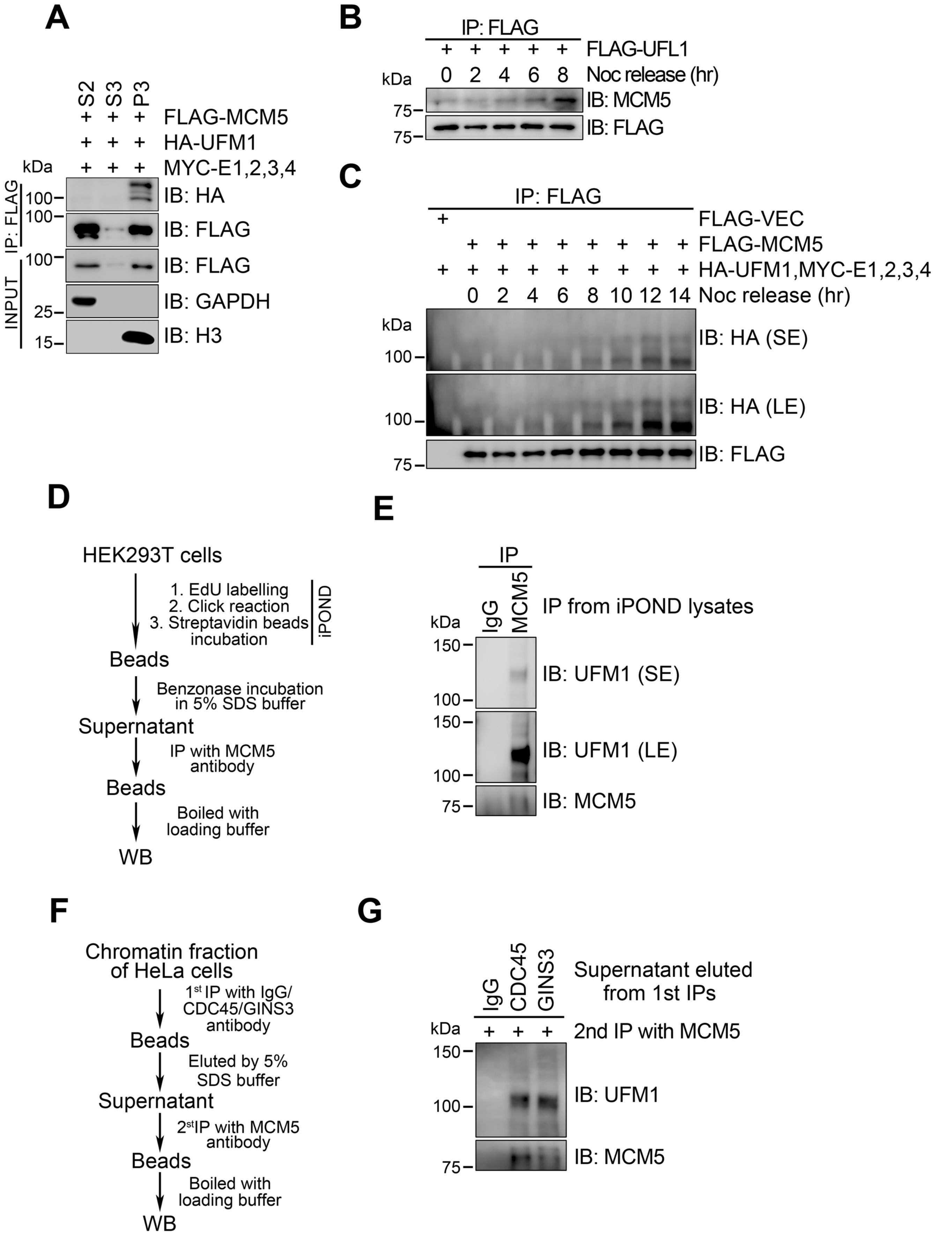
A
S2 S3 P3
FLAG-MCM5
HA-UFM1
MYC-E1,2,3,4
kDa
IP: FLAG
INPUT
100
100
100
25
15
IB: HA
IB: FLAG
IB: FLAG
IB: GAPDH
IB: H3
B
IP: FLAG
FLAG-UFL1
kDa
0 2 4 6 8
Noc release (hr)
75
75
IB: MCM5
IB: FLAG
C
IP: FLAG
FLAG-VEC
FLAG-MCM5
HA-UFM1,MYC-E1,2,3,4
0 2 4 6 8 10 12 14
Noc release (hr)
kDa
100
100
75
IB: HA (SE)
IB: HA (LE)
IB: FLAG
D
HEK293T cells
1. EdU labelling
2. Click reaction
3. Streptavidin beads incubation
iPOND
Beads
Benzonase incubation in 5% SDS buffer
Supernatant
IP with MCM5 antibody
Beads
Boiled with loading buffer
WB
E
IP
IgG
MCM5
IP from iPOND lysates
kDa
150
100
150
100
75
IB: UFM1 (SE)
IB: UFM1 (LE)
IB: MCM5
F
Chromatin fraction of HeLa cells
1st IP with IgG/ CDC45/GINS3 antibody
Beads
Eluted by 5% SDS buffer
Supernatant
2st IP with MCM5 antibody
Beads
Boiled with loading buffer
WB
G
IgG
CDC45
GINS3
Supernatant eluted from 1st IPs
kDa
2nd IP with MCM5
150
100
75
IB: UFM1
IB: MCM5

 © The Author(s)

Figure EV4. The UFMylation of MCM5 occurs at the ongoing replication fork.

(**A**) The cytoplasm (S2), nucleoplasm (S3), and chromatin (P3) fractions of HeLa cells expressing the indicated plasmids were subjected to IP with a FLAG antibody and western blotting with the indicated antibodies. (**B**) HeLa cells expressing FLAG-UFL1 were synchronized with nocodazole (as in Fig. 3F) and then lysed at the indicated times. UFL1 was purified on FLAG M2 beads and analyzed by western blotting. (**C**) HeLa cells expressing UFMylation factors were synchronized as in (**B**). After release from the nocodazole block, the cells were lysed at the indicated times. MCM5 was purified on FLAG M2 beads and subjected to western blotting with the indicated antibodies. SE short exposure, LE long exposure. (**D**) Schematic of the combined IP and iPOND assay. (**E**) HEK293T cell lysates were purified as in (**D**) and subjected to western blotting with the indicated antibodies. (**F**) Schematic of the two-step IP. (**G**) HeLa cell lysates were purified as in (**F**) and subjected to western blotting with the indicated antibodies.

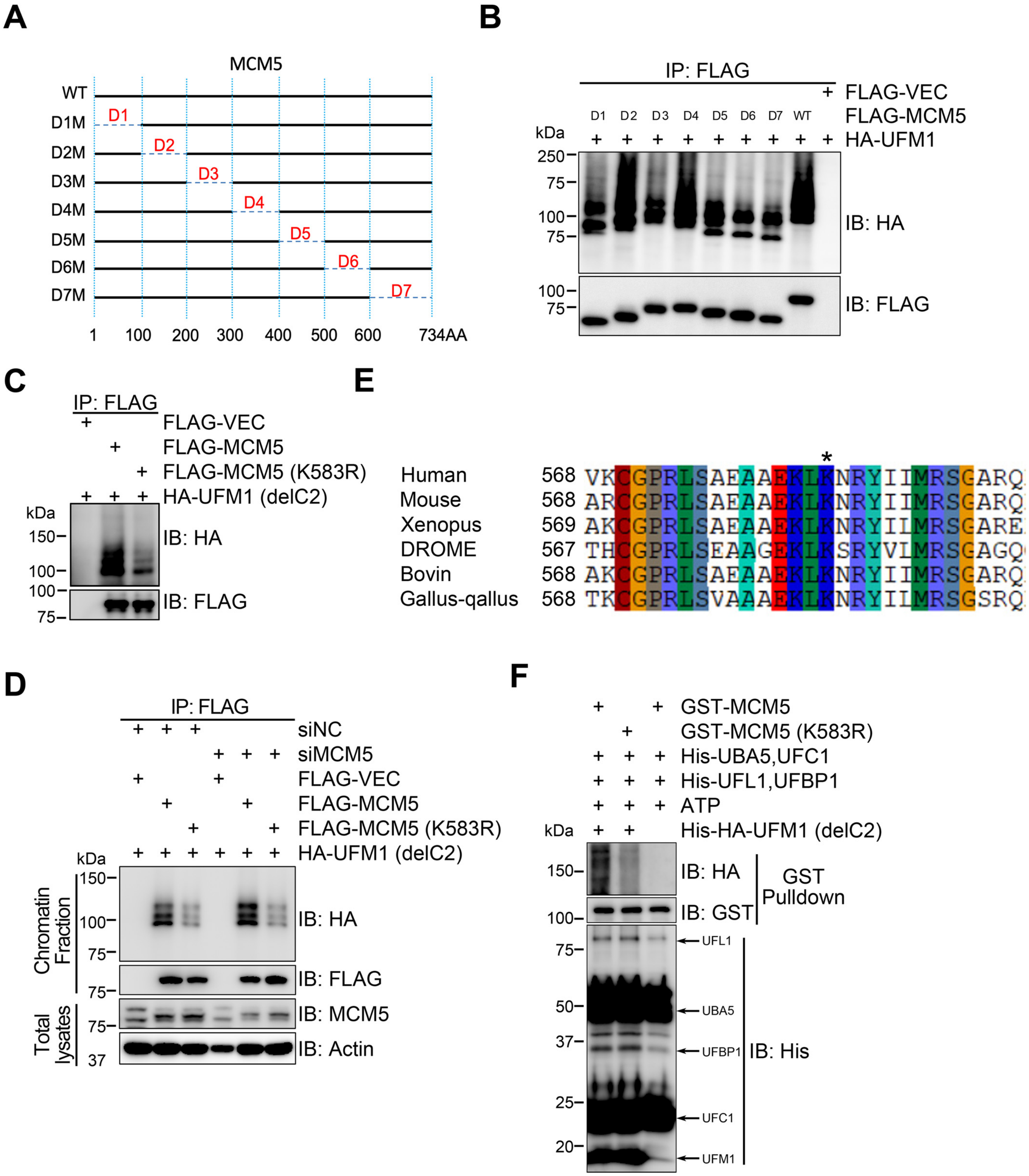
A
MCM5
WT
D1M
D2M
D3M
D4M
D5M
D6M
D7M
D1
D2
D3
D4
D5
D6
D7
1 100 200 300 400 500 600 734AA
B
IP: FLAG
FLAG-VEC
FLAG-MCM5
HA-UFM1
D1 D2 D3 D4 D5 D6 D7 WT
kDa
250
75
100
75
IB: HA
100
75
IB: FLAG
C
IP: FLAG
FLAG-VEC
FLAG-MCM5
FLAG-MCM5 (K583R)
HA-UFM1 (delC2)
kDa
150
100
IB: HA
100
75
IB: FLAG
E
Human 568 VKCGPRLSAEAAEKLKNRYIIMRSGARQ
Mouse 568 ARCGPRLSAEAAEKLKNRYIIMRSGARQ
Xenopus 569 AKCGPRLSAEAAEKLKNRYILMRSGARE
DROME 567 THCGPRLSEAAGEKLKSRYVLMRSGAGQ
Bovin 568 AKCGPRLSAEAAEKLKNRYIIMRSGARQ
Gallus-qallus 568 TKCGPRLSVAAAEKLKNRYIIMRSGSRQ
D
IP: FLAG
siNC
siMCM5
FLAG-VEC
FLAG-MCM5
FLAG-MCM5 (K583R)
HA-UFM1 (delC2)
kDa
150
100
75
75
75
37
Chromatin Fraction
Total lysates
IB: HA
IB: FLAG
IB: MCM5
IB: Actin
F
GST-MCM5
GST-MCM5 (K583R)
His-UBA5,UFC1
His-UFL1,UFBP1
ATP
His-HA-UFM1 (delC2)
kDa
150
100
75
50
37
25
20
IB: HA
IB: GST
GST Pulldown
UFL1
UBA5
UFBP1
UFC1
UFM1
IB: His

 © The Author(s)

Figure EV5. MCM5 is UFMylated on Lys583.

(**A**) The structure of MCM5 and the various deletions D1–D7. (**B**) HEK293T cells were transfected with the indicated plasmids, and the lysates were immunoprecipitated with a FLAG antibody before western blotting with the indicated antibodies. (**C**) HEK293T cells were transfected with the indicated plasmids and treated as in (**B**). (**D**) HeLa cells were transfected with the indicated plasmids, and the chromatin fraction were isolated for subsequent immunoprecipitation and western blotting as in (**B**). (**E**) Sequence alignment of the region encompassing the UFMylation site of MCM5 from indicated species. The asterisk shows the position of the K583. (**F**) Bacterially produced UFMylation factors [His-UBA5, His-UFC1, His-UFL1, and His-HA-UFM (delC2)] and bacterially produced GST-MCM5 or GST-MCM5 (K583R) were incubated with GST beads before analysis by western blotting with the indicated antibodies.

© The Author(s)

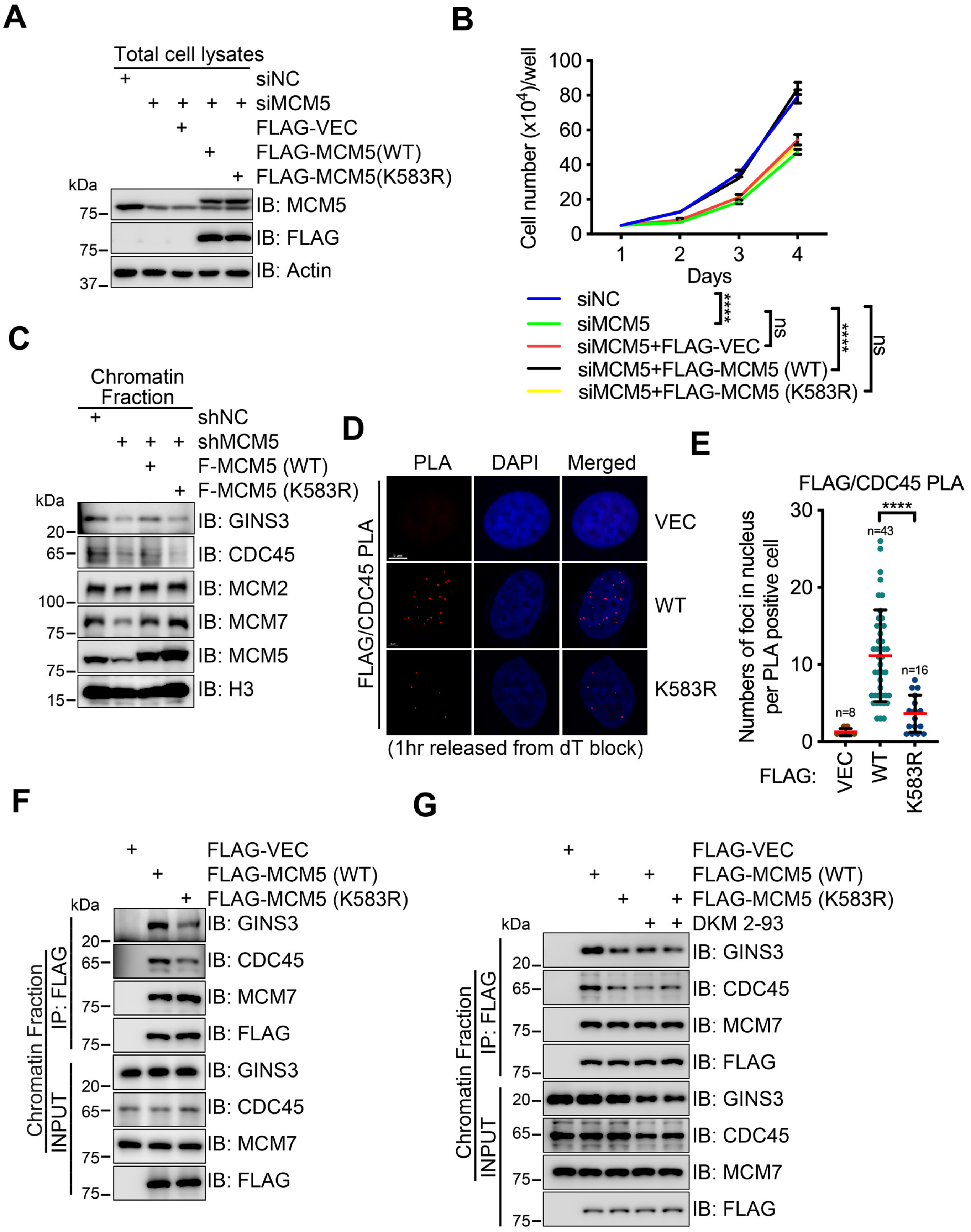
A
Total cell lysates
siNC
siMCM5
FLAG-VEC
FLAG-MCM5(WT)
FLAG-MCM5(K583R)
kDa
IB: MCM5
IB: FLAG
IB: Actin
B
Cell number (x10^4)/well
Days
siNC
siMCM5
siMCM5+FLAG-VEC
siMCM5+FLAG-MCM5 (WT)
siMCM5+FLAG-MCM5 (K583R)
****
ns
C
Chromatin Fraction
shNC
shMCM5
F-MCM5 (WT)
F-MCM5 (K583R)
IB: GINS3
IB: CDC45
IB: MCM2
IB: MCM7
IB: MCM5
IB: H3
D
PLA
DAPI
Merged
FLAG/CDC45 PLA
VEC
WT
K583R
(1hr released from dT block)
E
FLAG/CDC45 PLA
Numbers of foci in nucleus per PLA positive cell
n=8
n=43
n=16
FLAG:
VEC
WT
K583R
F
FLAG-VEC
FLAG-MCM5 (WT)
FLAG-MCM5 (K583R)
Chromatin Fraction
IP: FLAG
INPUT
IB: GINS3
IB: CDC45
IB: MCM7
IB: FLAG
G
DKM 2-93

 © The Author(s)

Figure EV6. MCM5 UFMylation at K583 stabilizes CMG helicase complex formation.

(**A**) Western blot analysis of the indicated proteins in HeLa cells transfected with indicated siRNAs and plasmids. (**B**) Growth curves of the HeLa cells used in (**A**). The mean number of cells (biological replicates, $n = 3$) ± SD is shown. *P* values were calculated by Tukey's multiple comparisons test (****$P < 0.0001$; ns $P > 0.05$, no significance). *P* values: siNC vs siMCM5, 3.17e-005; siMCM5 vs siMCM5+FLAG-VEC, 0.99; siMCM5 vs siMCM5+FLAG-MCM5 (WT), 1.58e-005; siMCM5 vs siMCM5+FLAG-MCM5 (K583R), 0.93. (**C**) Western blot analysis of the proteins in chromatin fractions of HeLa cells transiently transfected with the indicated shRNA and plasmids. (**D**, **E**) Immunostaining of PLA foci in HeLa cells transiently transfected with either FLAG-VEC, FLAG-MCM5 (WT), or FLAG-MCM5 (K583R) at 1 h released from the dT block. Representative PLA foci (red) images are shown in (**D**), and the quantitative data are shown in (**E**). *n* cell number. *P* values was calculated by unpaired *t* test with Welch's correction (****$P < 0.0001$). *P* value: WT vs K583R, 4.62e-009. Scale bar, 5 µm. (**F**, **G**) Western blot analysis of proteins in anti-FLAG immunoprecipitants enriched from the chromatin fraction of HeLa cells transfected with indicated plasmids. The HeLa cells in (**G**) were treated with DKM 2-93 (100 µM) for 16 h before IP of the chromatin fraction.

© The Author(s)

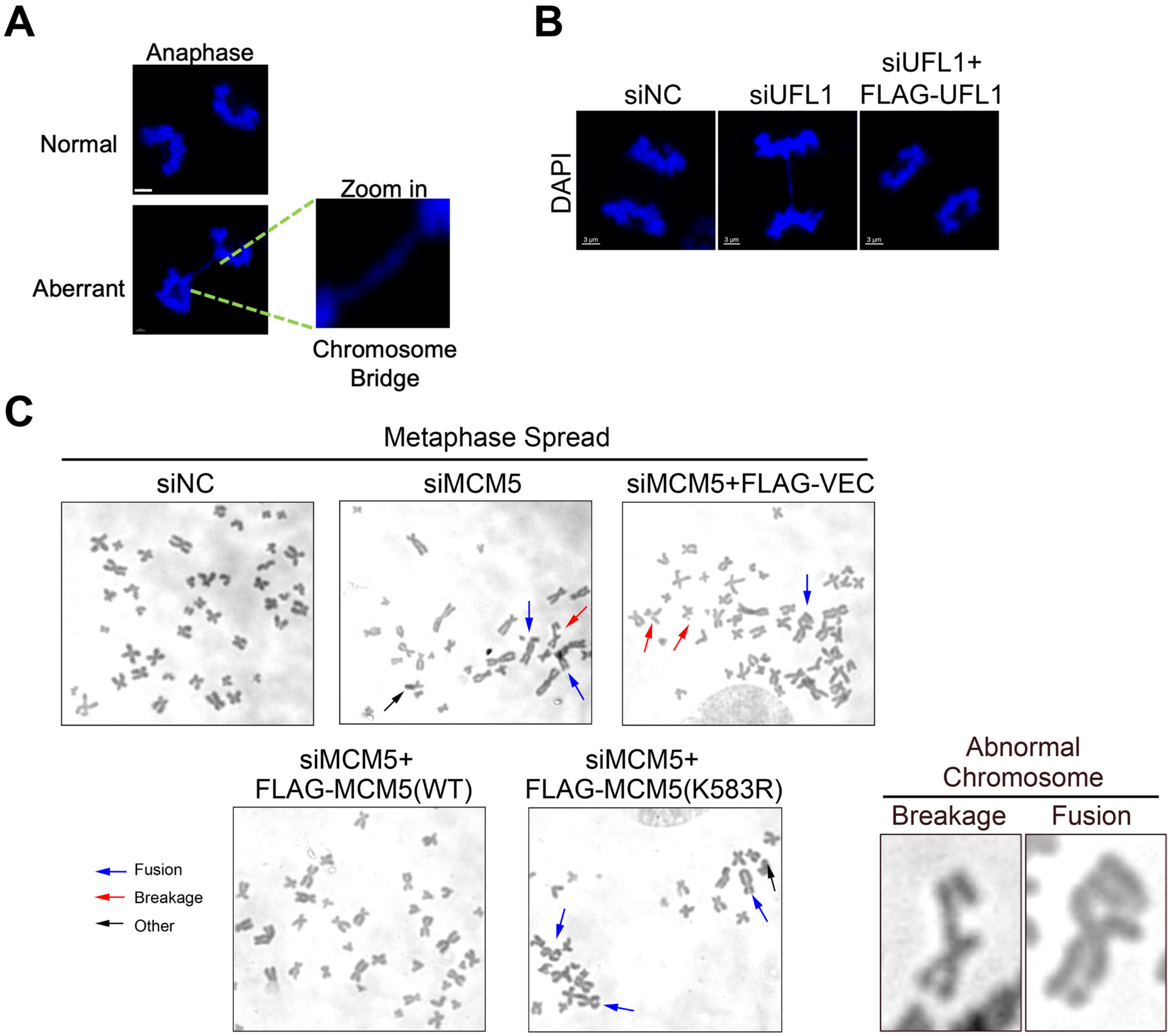

Figure EV7. MCM5 UFMylation promotes cell growth and maintains genome stability.

(**A**, **B**) Representative images of chromosome bridge events in HeLa cells during cell cycle anaphase. The Hela cells in (**A**) were fixed and permeabilized before staining with DAPI (blue). The HeLa cells in (**B**) were transfected with the indicated siRNAs and FLAG-UFL1 and were treated as in (**A**). Scale bar, 3 μm. (**C**) Representative images of chromosomes obtained from HeLa cells transfected with the indicated siRNA oligos and plasmids. The chromosomes were stained with Giemsa before images were captured. The abnormal chromosomes are shown at a higher magnification in the right panel.

 © The Author(s)
